# Supplementary material for: An unusual endo-selective C-H hydroarylationof norbornene by the Rh(I)-catalyzed reactionof benzamides
Source: Nat Commun. 2017 Nov 13;8:1448. doi: 10.1038/s41467-017-01531-2 (PMC5682280; doi:10.1038/s41467-017-01531-2)
Supplement: Supplementary file 1 — Supplementary Information [file 41467_2017_1531_MOESM1_ESM.pdf]

Supplementary methods

General Information.

^{1}H NMR and ^{13}C NMR spectra were recorded on a JEOL ECS-400 spectrometer and Bruker AVANCE III spectrometer in $CDCl_3$ with tetramethylsilane as the internal standard. Data are reported as follows: chemical shift in ppm (δ), multiplicity (s = singlet, d = doublet, t = triplet, q = quartet, brs = broad singlet, and m = multiplet), coupling constant (Hz), and integration. In some cases, some peaks in the ^{13}C NMR spectra cannot be analyzed because of overlapping peaks. Infrared spectra (IR) were obtained using a JASCO FT/IR-4200 spectrometer; absorptions are reported in reciprocal centimeters with the following relative intensities: s (strong), m (medium), or w (weak). Mass spectra and high resolution mass spectra (HRMS) were obtained using a JEOL JMS-700 spectrometer. Melting points were determined using a Yamato melting point apparatus. Column chromatography was performed with SiO_2 (Silicycle SiliaFlash F60 (230-400 mesh)). Some compounds were purified by LC-908 HPLC (GPC).

Materials.

Toluene (Kanto Chemical) was purified by passage through activated alumina using a GlassContour Solvent Dispensing System. Pivalic acid (CAS 75-98-9) was purchased from Nacalai Tesque. 8-Aminoquinoline (CAS 578-66-5), 2-norbornene (CAS 498-66-8), 2,6-dimethylbenzoic acid (CAS 632-46-2) were purchased from Tokyo Chemical Industry Co., Ltd. $[Rh(OAc)(cod)]_2$ was prepared according to literature procedures.[1]

Synthesis of Starting Materials.

All amides bearing an 8-aminoquinoline moiety were prepared by reacting the corresponding acid or the corresponding acid chlorides with 8-aminoquinoline.[2]

General Procedure for the Preparation of Stating Amides.

Synthesis of amides from acid chlorides.

The acid chloride (15 mmol) was dissolved in CH_2Cl_2 (20 mL). After cooling the reaction mixture to 0 °C, a solution of 8-aminoquinoline (15 mmol) and triethylamine (36 mmol) in 10 mL of CH_2Cl_2 was added dropwise. The resulting mixture was allowed to warm to rt and was then stirred overnight. The crude mixture was then washed with saturated aqueous $NaHCO_3$ (20 mL), and CH_2Cl_2 (3x20 mL). The combined organic layers were washed with 1 M HCl aq. (20 mL). The organic phase was dried over anhydrous Na_2SO_4 and the solution taken to dryness.The resulting crude amide was purified by flash chromatography on silica gel (eluent: hexanes/EtOAc = 5/1).

Synthesis of amides from carboxylic acid.

To a stirred solution of carboxylic acid (15 mmol) and DMF (5 drops) in CH_2Cl_2 (10 mL), $(COCl)_2$ (1.5 mL, 18 mmol) was added dropwise. The solution was magnetically stirred at room temperature for 2 h. The solvent was then eliminated under reduced pressure, and the resulting residue was dissolved in CH_2Cl_2 (15 mL). After cooling the reaction mixture to 0 °C, a solution of 8-Aminoquinoline (15 mmol) and triethylamine (36 mmol) in 10 mL of the same solvent were added dropwise. The resulting mixture was allowed to warm to rt and stirred overnight. The crude product was washed with saturated aqueous $NaHCO_3$ (20 mL), and CH_2Cl_2 (3x20 mL). The organic phase was washed with 1 M HCl aq. (20 mL).The organic phase was dried over anhydrous Na_2SO_4 and the solvent removed by evaporation of the solvent. The resulting crude amide was purified by flash chromatography on silica gel (eluent: hexanes/EtOAc = 5/1).

Preparation of 1,4-dihydro-1,4-methanonaphthalene-*d*$_6$

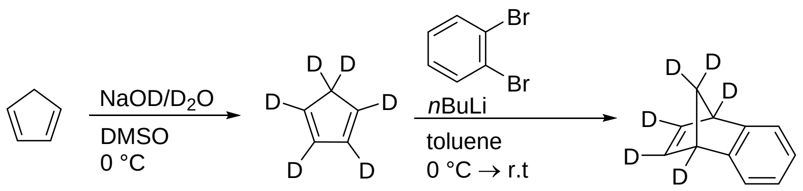

Synthesis of 1-cyclopentadiene-*d*$_6$[3]

To 20 mL of D_2O, cooled to 0°C, was added 1.1 g of Na at a sufficiently slow rate to keep the temperature below 10 °C. After the addition was complete, 10 mL of this $NaOD/D_2O$ solution was syringed into a flask containing 9 mL of freshly distilled cyclopentadiene and 10 mL of DMSO at 0°C. The mixture was then stirred vigorously for 1 h. The resulting layers were separated, and the top layer (cyclopentadiene) was syringed into another flask containing 10 mL of the $NaOD/D_2O$ solution and 10 mL of DMSO at 0°C. The mixture was again stirred for 1 h, and the layers were separated. This entire procedure was repeated 3 times for a total of six exchanges.

Synthesis of 1,4-dihydro-1,4-methanonaphthalene-*d*$_6$[4]

1,2-Dibromobenzene (4.72 g, 20.0 mmol) and cyclopentadiene-*d*$_6$ (1.44 g, 20.0 mmol), in toluene (24.0 mL) was stirred at 0 °C under an atmosphere of nitrogen. To this solution *n*-BuLi (12.5 mL, 1.6 M in hexane, 20.0 mmol) was added dropwise, during which the color of the solution first became yellow then white. The mixture was allowed to warm to room temperature and, after 12h, was treated with H_2O (20.0 mL) and the resulting solution was extracted with hexane (3×10.0 mL). The organic layer was dried over Na_2SO_4, and the solvent removed under reduced pressure. The solid residue was purified by flash column chromatography (SiO_2, hexane) to give **2** as a colorless liquid. (2.1 g, 71%).

General procedure for the Rh(I)-catalyzed hydroarylation of aromatic amides with norbornene. To an oven-dried 5 mL screw-capped vial, 3-fluoro-2-methyl-*N*-(quinolin-8-yl)benzamide (**1n**) (84 mg, 0.3 mmol), 2-norbornene (57 mg, 0.6 mmol), $[Rh(OAc)(cod)]_2$ (4.1 mg, 0.0075 mmol), pivalic acid (92 mg, 0.9 mmol) and toluene (0.5 mL) were added. The mixture was stirred for 12 h at 160 °C and then allowed to cool. The resulting mixture was filtered through a celite pad and the filtrate was washed with saturated aqueous $NaHCO_3$ (10 mL) and the organic phase concentrated in vacuo. The residue was purified by column chromatography on silica gel (eluent: hexane/EtOAc= 50/1) to afford the alkylation product **2n** (104.4 mg, 93%, endo:exo= 16.3:1) as a colorless oil.

Spectroscopic Data.

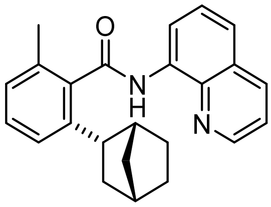

2-(Bicyclo[2.2.1]heptan-2-yl)-6-methyl-*N*-(quinolin-8-yl)benzamide (2a)

59% yield. endo : exo = 15.0 : 1. R_f 0.11 (hexane/EtOAc = 10/1). Colorless oil. ^1H NMR ($CDCl_3$, 400 MHz) (endo) δ 1.19-1.70 (m, 7H), 1.93 (m,1H), 2.26 (brs, 1H), 2.42 (s, 3H), 2.45 (s, 1H), 3.46 (t, J = 5.5 Hz, 1 H), 7.22 (d, J = 4.1 Hz, 1H), 7.32 (m, 1H), 7.44 (dd, J = 4.1 Hz, 1H), 7.59 (m, 2H), 8.18 (dd, J = 8.2, 1.4 Hz, 1H), 8.73 (dd, J = 1.8 Hz, 1H), 8.99 (dd, J = 7.3, 1.4 Hz, 1H), 9.91 (brs, 1H), (exo) 2.22 (brs, 1H), 2.95 (t, J = 7.3 Hz, 1H); ^{13}C NMR ($CDCl_3$, 100 MHz) δ (endo) 19.67, 23.21, 29.95, 35.70, 37.80, 41.59, 43.54, 43.66, 116.98, 121.80, 122.04, 125.03, 127.59, 127.93, 128.18, 128.49, 134.59 (two overlapping peaks), 136.46, 138.70, 139.23, 140.15, 148.42 (exo) 28.56, 30.78, 36.71, 36.94, 40.29, 44.31, 116.87, 123.27, 125.54, 129.09; IR (neat) 3346 w, 2952 w, 2871 w, 1673 m, 1595 w, 1578 w, 1517 s, 1481 s, 1423 m, 1385 m, 1325 m, 1262 w, 1127 w, 898 w, 826 m, 790 m, 754 s, 689 w, 667 w; MS *m/z* (relative intensity, %) 213 (45), 212 (100), 183 (11), 171 (18), 147 (15), 146 (13), 145 (33), 144 (32), 143 (16), 129 (15), 128 (15), 117 (11), 116 (10), 115 (22), 105 (20), 91 (14), 67 (44); HRMS Calcd for $C_{24}H_{24}N_2O$: 356.1189; Found: 356.1889.

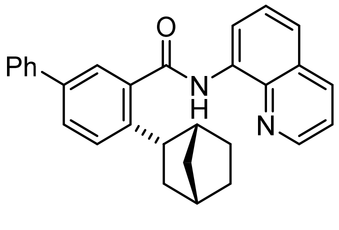

2-(Bicyclo[2.2.1]heptan-2-yl)-5-phenyl-*N*-(quinolin-8-yl)benzamide (2b)

79% yield. endo : exo = 13.6 : 1. R_f 0.11 (hexane/EtOAc = 10/1). Colorless oil. ^1H NMR ($CDCl_3$,

400 MHz) (endo) δ 1.20-1.69 (m, 7H), 2.02 (tt, *J* = 11.9, 3.7 Hz, 1H), 2.30 (brs, 1H), 2.60 (s, 1H), 3.76 (dt, *J* = 11.4, 5.1 Hz, 1 H), 7.33-7.36 (m, 1H), 7.42-7.51 (m, 4H), 7.53-7.70 (m, 5H), 7.81 (d, *J* = 1.8 Hz, 1H), 8.17 (dd, *J* = 8.5, 1.6 Hz, 1H), 8.74 (dd, *J* = 2.0 Hz, 1H), 8.97 (dd, *J* = 7.6, 1.1 Hz, 1H), 10.15 (brs, 1H), (exo) 1.87 (m, 1H), 2.56 (s, 1H), 3.30 (dd, *J* = 9.2, 6.0 Hz, 1H); ^{13}C NMR ($CDCl_3$, 100 MHz) δ (endo) 23.30, 30.12, 35.58, 37.75, 41.33, 42.95, 43.16, 116.83, 121.81, 122.02, 125.90, 127.15, 127.57, 128.06, 128.14, 128.76, 129.00, 134.88, 136.47, 138.70, 138.84, 139.09, 140.32, 140.62, 148.44, 169.25 (exo) 28.74, 30.67, 36.88, 37.13, 40.35, 43.62, 126.86, 128.46, 128.64, 137.88; IR (neat) 3347 w, 2952 w, 2871 w, 1671 m, 1519 s, 1481 m, 1423 w, 1385 w, 1326 m, 1245 w, 1216 m, 906 w, 825 w, 752 s, 697 m, 668 w; MS *m/z* (relative intensity, %) 418 (9), 275 (26), 274 (100), 247 (10), 246 (49), 245 (28), 233 (17), 218 (32), 217 (10), 207 (17), 205 (11), 203 (11), 202 (13), 195 (11), 191 (12), 179 (18), 178 (37), 167 (28), 166 (12), 165 (26), 152 (21), 144 (58), 129 (13), 115 (10), 91 (19), 77 (12), 67 (48); HRMS Calcd for $C_{29}H_{26}N_2O$: 418.1606; Found: 418.2048.

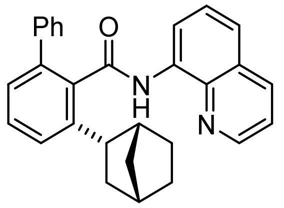

2-(Bicyclo[2.2.1]heptan-2-yl)-6-phenyl-*N*-(quinolin-8-yl)benzamide (2c)

67% yield. endo : exo = 5.6 : 1. R_f 0.11 (hexane/EtOAc = 10/1). Colorless oil. ^{1}H NMR ($CDCl_3$, 400 MHz) (endo) δ 0.83-1.81 (m, 7H), 1.99 (t, *J* =12.1 Hz, 1H), 2.30 (brs, 1H), 2.52 (brs, 1H), 3.63 (s, 1H), 7.07 (dd, *J* = 6.7 Hz, 1 H), 7.19 (dd, *J* = 6.7 Hz, 2H), 7.25 (d, *J* =1.4 Hz, 1H), 7.30 (d, *J* = 7.3 Hz, 1H), 7.35 (dd, *J* = 4.1 Hz, 1H), 7.46 (m, 6H), 8.07 (d, *J* = 8.2 Hz, 1H), 8.61 (d, *J* = 4.1 Hz, 1H), 8.72 (d, *J* = 7.3 Hz, 1H), 9.60 (brs, 1H), (exo) 3.16 (t, *J* = 7.1 Hz, 1H); ^{13}C NMR ($CDCl_3$, 100 MHz) δ (endo) 23.34, 30.02, 35.73, 37.85, 41.64, 43.47, 116.64, 121.57, 121.72, 126.69, 127.25, 127.41, 127.84, 127.91, 128.64, 128.87, 134.55, 136.20, 138.14, 138.53, 139.90, 140.74, 141.11, 148.10, 168.69; IR (neat) 3345 w, 2952 w, 2871 w, 2363 w, 1673 m, 1578 w, 1520 s, 1482 m, 1424 w, 1386 w, 1326 m, 1265 w, 1174 w, 1130 w, 896 w, 826 w, 791 w, 759 w, 700 w; MS *m/z* (relative intensity, %) 418 (17), 276 (11), 275 (62), 274 (100), 207 (12), 179 (10), 178 (15), 165 (11), 144 (13); HRMS Calcd for $C_{29}H_{26}N_2O$: 418.1606; Found: 418.2045.

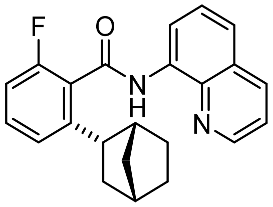

2-(Bicyclo[2.2.1]heptan-2-yl)-6-fluoro-*N*-(quinolin-8-yl)benzamide (2d)

66% yield. endo : exo = 19.0 : 1. R_f 0.17 (hexane/EtOAc = 20/1). Colorless oil. ^{1}H NMR ($CDCl_3$, 400 MHz) (endo) δ 1.22-1.69 (m, 7H), 1.97 (tt, *J* = 12.0, 3.6 Hz, 1H), 2.29 (brs, 1H), 2.51 (brs, 1H), 3.56 (dt, *J* = 11.6, 5.0 Hz, 1 H), 7.01 (t, *J* = 8.8 Hz, 1H), 7.18 (d, *J* = 8.2 Hz, 1H), 7.40 (dd, *J* = 14.2, 7.8 Hz, 1H), 7.46 (dd, *J* = 4.1 Hz, 1H), 7.60 (m, 2H), 8.19 (dd, *J* = 8.2 Hz, 1H), 8.77 (dd, *J* = 4.1 Hz, 1H), 8.97 (d, *J* = 7.3 Hz, 1H), 10.06 (brs, 1H), (exo) 3.08 (dd, *J* = 7.1 Hz, 1H); ^{13}C NMR ($CDCl_3$, 100 MHz) δ (endo) 21.21, 29.96, 35.46, 37.04, 37.69, 41.36, 42.98, 43.24, 43.44, 113.33 (d, *J* = 22 Hz), 117.07, 121.18, 122.27, 123.45 (d, *J* = 2 Hz), 126.78 (d, *J* = 17 Hz), 127.56, 128.15, 130.13 (d, *J* = 9 Hz), 134.51, 136.48, 138.60, 144.00, 148.48, 159.51 (d, *J* = 245 Hz); IR (neat) 3345 w, 2954 w, 2872 w, 1722 m, 1680 m, 1611 w, 1573 w, 1523 s, 1483 w, 1523 s, 1483 m, 1425 m, 1384 m, 1327 m, 1303 m, 1258 m, 1207 m, 1126 m, 1062 w, 1016 w, 900 w, 855 w, 826 w, 793 w, 768 m, 683 w; MS *m/z* (relative intensity, %) 361 (15), 360 (59), 217 (18), 216 (100), 188 (49), 187 (17), 175 (25), 149 (16), 144 (37); HRMS Calcd for $C_{23}H_{21}FN_2O$: 360.1638; Found: 360.1636.

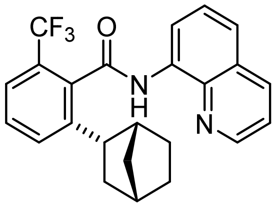

2-(Bicyclo[2.2.1]heptan-2-yl)-6-trifluoromethyl-*N*-(quinolin-8-yl)benzamide (2e)

85% yield. endo : exo = 8.2 :1. R_f 0.11 (hexane/EtOAc = 20/1). Colorless oil. ^{1}H NMR ($CDCl_3$, 400 MHz) (endo) δ 0.92- 1.80 (m, 7H), 1.97 (m,1H), 2.29 (brs, 1H), 2.48 (m, 1H), [3.45, 3.61 (two triplet peaks, *J* = 5.5 Hz, 1 H)], 7.44 (dd, *J* = 4.1 Hz, 1H), 7.56 (m, 5H), 8.17 (dd, *J* = 8.2 Hz, 1H), 8.73 (dd, *J* = 3.7 Hz, 1H), 8.95 (dd, *J* = 6.9 Hz, 1H), 9.98 (brs, 1H), (exo) [3.00, 3.06 (two broad singlet, 1H)]; ^{13}C NMR ($CDCl_3$, 150 MHz) δ 23.15, 23.25, 28.42, 29.84, 29.88, 35.87, 36.18, 36.88, 37.72, 37.91, 41.68, 41.80, 42.93, 43.41, 43.87, 44.23, 117.04, 117.12, 117.19, 121.35, 121.83, 122.31, 122.43, 123.17, 123.86, 124.99, 126.80, 127.39, 127.57, 127.67, 127.78, 128.00, 128.16, 128.58, 128.74, 128.80, 128.90, 128.43, 131.36, 131.66, 134.19, 134.35, 134.53, 136.44, 136.55, 137.68, 138.63, 142.22, 142.56, 148.45, 148.51, 165.96, 166.27; IR (neat) 3341 w, 2954 w, 2873 w, 1678 m, 1597 w, 1578 w, 1521 s, 1483 m, 1424 m, 1386 m, 1319 w, 1260 w, 1211 w, 1169 m, 1127 s, 1088 w, 899 w, 825 m, 791 m, 755 s, 668 w; MS *m/z* (relative intensity, %) 410 (10), 267 (10), 266 (45), 238 (14),

237 (10), 225 (28), 205 (13), 199 (23), 197 (11), 171 (13), 151 (20), 145 (19), 144 (100), 130 (11), 129 (12), 116 (11), 67 (46); HRMS Calcd for $C_{24}H_{21}F_3N_2O$:410.1606; Found: 410.1602.

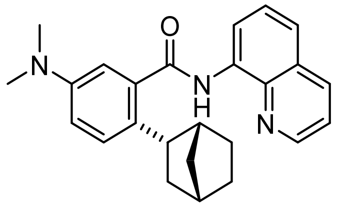

2-(Bicyclo[2.2.1]heptan-2-yl)-5-dimethylamino -*N*-(quinolin-8-yl)benzamide (2f)

94% yield. endo : exo = 9.8 : 1. R_f 0.16 (hexane/EtOAc = 5/1). Yellow oil. ^{1}H NMR ($CDCl_3$, 400 MHz) (endo) δ 1.15-1.69 (m, 7H), 1.95 (tt, *J* = 11.9, 3.8 Hz, 1H), 2.25 (brs, 1H), 2.50 (brs, 1H), 2.96 (s, 6H), 3.61 (dt, *J* = 11.9, 5.0 Hz, 1 H), 6.84 (dd, *J* = 8.7, 2.7 Hz, 1H), 6.95 (d, *J* = 2.8 Hz, 1H), 7.27 (d, *J* = 8.7 Hz, 1H), 7.43 (dd, *J* = 4.3 Hz, 1H), 7.54 (dd, *J* = 8.2, 1.4 Hz, 1H), 7.60 (t, *J* = 7.8 Hz, 1H), 8.16 (dd, *J* = 8.2, 1.8 Hz, 1H), 8.75 (dd, *J* = 4.1, 1.4 Hz, 1H), 8.96 (dd, *J* = 7.6, 1.1 Hz, 1H), 10.10 (brs, 1H), (exo) 2.29 (brs, 1H), 2.47 (brs, 1H), 3.14 (dd, *J* = 7.3 Hz); ^{13}C NMR ($CDCl_3$, 100 MHz) δ (endo) 23.23, 30.16, 35.60, 37.70, 40.80, 41.16, 42.17, 43.11, 111.57, 113.71, 116.71, 121.73, 121.76, 127.56, 128.11, 128.66, 128.94, 135.03, 136.39, 138.73, 139.15, 148.32, 148.66, 169.98 (exo) 28.77, 30.66, 36.69, 37.03, 40.29, 42.97, 111.69, 114.23, 127.06; IR (neat) 3349 w, 2950 w, 2871 w, 2804 w, 1719 w, 1674 m, 1607 w, 1519 s, 1481 s, 1423 w, 1384 m, 1356 w, 1325 m, 1257 w, 1229 w, 1211 m, 1158 w, 1130 w, 1096 w, 1063 w, 973 w, 907 w, 824 m, 790 m, 753 s, 695 w; MS *m/z* (relative intensity, %) 386 (15), 385 (53), 242 (25), 241 (100), 214 (33), 213 (94), 212 (28), 186 (12), 185 (58), 174 (21), 172 (10), 169 (13), 162 (22), 153 (12), 146 (12), 134 (15); HRMS Calcd for $C_{29}H_{26}N_2O$: 385.2154; Found: 385.2149.

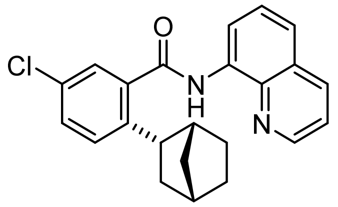

2-(Bicyclo[2.2.1]heptan-2-yl)-5-chloro -*N*-(quinolin-8-yl)benzamide (2g)

65% yield. endo : exo = 9.2 : 1. R_f 0.14 (hexane/EtOAc = 20/1). Colorless oil. ^{1}H NMR ($CDCl_3$, 400 MHz) (endo) δ 1.14-1.38 (m, 5H), 1.49-1.63 (m, 2H), 1.98 (tt, *J* = 12.2, 3.6 Hz, 1H), 2.28 (brs, 1H), 2.53 (brs, 1H), 3.68 (dt, *J* = 11.5, 5.3 Hz, 1 H), 7.33 (d, *J* = 8.7 Hz, 1H), 7.40 (td, *J* = 9.0, 2.4 Hz, 1H), 7.47 (dd, *J* = 4.0 Hz, 1H), 7.57 (m, 1H), 8.18 (dd, *J* = 8.2, 1.8 Hz, 1H), 8.78 (dd, *J* = 2.0 Hz, 1H), 8.91 (dd, *J* = 6.9, 1.8 Hz, 1H), 10.06 (brs, 1H), (exo) 1.82 (tt, *J* = 9.8, 2.6 Hz, 1H), 2.30 (brs,

1H), 2.48 (brs, 1H), 3.21 (dd, *J* = 9.2, 6.0 Hz); ^{13}C NMR ($CDCl_3$, 100 MHz) δ (endo) 23.16, 30.02, 35.56, 37.66, 41.24, 42.72, 43.00, 116.92, 121.90, 122.26, 127.26, 127.51, 128.13, 129.50, 129.72, 131.60, 134.58, 136.51, 138.64, 139.91, 140.15, 148.51, 167.69 (exo) 28.63, 30.58, 36.78, 37.10, 40.36, 42.82, 43.43, 127.86, 130.05; IR (neat) 3341 w, 2953 w, 2872 w, 1721 w, 1675 m, 1521 s, 1481 m, 1424 m, 1385 m, 1326 w, 1304 w, 1257 w, 1209 w, 1128 w, 1060 w, 908 w, 824 w, 790 m, 754 s, 687 w, 668 w; MS *m/z* (relative intensity, %) 376 (14), 234 (31), 233 (17), 232 (93), 206 (20), 205 (15), 204 (61), 203 (23), 193 (11), 191 (33), 179 (12), 176 (21), 169 (13), 167 (11), 165 (27), 163 (10), 149 (11), 145 (19), 144 (100), 141 (13), 137 (10), 130 (22), 129 (19), 128 (21), 127 (13), 125 (18), 117 (12), 116 (16), 115 (22), 102 (24), 101 (20), 89 (11), 67 (42); HRMS Calcd for $C_{23}H_{21}ClN_2O$: 376.1342; Found: 376.1345.

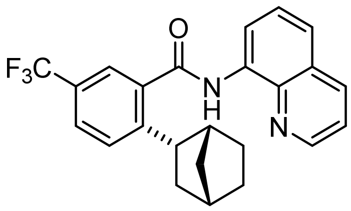

2-(Bicyclo[2.2.1]heptan-2-yl)-5-trifluoromethyl-*N*-(quinolin-8-yl)benzamide (2h)

88% yield. endo : exo = 11.8 : 1. R_f 0.14 (hexane/EtOAc = 20/1). Colorless oil. ^{1}H NMR ($CDCl_3$, 400 MHz) (endo) δ 1.16-1.69 (m, 7H), 2.02 (tt, *J* = 12.1, 3.6 Hz, 1H), 2.30 (brs, 1H), 2.59 (brs, 1H), 3.76 (dt, *J* = 11.4, 4.9 Hz, 1 H), 7.33 (d, *J* = 8.7 Hz, 1H), 7.46 (dd, *J* = 4.1 Hz, 1H), 7.52 (d, *J* = 8.2 Hz, 1H), 7.59 (m, 2H), 7.70 (d, *J* = 9.6 Hz, 1H), 7.81 (d, *J* = 14.2 Hz, 1H), 8.18 (dd, *J* = 8.2, 1.4 Hz, 1H), 8.77 (dd, *J* = 1.8 Hz, 1H), 8.93 (dd, *J* = 6.9, 1.8 Hz, 1H), 10.11 (brs, 1H), (exo) 1.86 (tt, *J* = 10.8, 2.6 Hz, 1H), 2.53 (brs, 1H), 3.29 (dd, *J* = 8.7, 6.4 Hz); ^{13}C NMR ($CDCl_3$, 150 MHz) δ (endo) 23.22, 29.93, 35.58, 37.68, 41.32, 43.09, 43.35, 117.02, 121.93, 122.39, 124.23 (d, *J* = 3.5 Hz), 124.08 (q, *J* = 270.3 Hz), 126.18 (d, *J* = 3.5 Hz), 126.89, 127.51, 128.15, 128.33 (q, *J* = 32.7 Hz), 128.80, 134.51, 136.54, 138.65, 139.06, 145.94, 148.56, 167.87 (exo) 28.60, 29.84, 30.61, 35.47, 36.88, 37.14, 40.35, 42.78, 43.98, 116.95, 137.87; IR (neat) 3345 w, 2953 w, 2873 w, 2360 w, 1722 w, 1677 m, 1522 s, 1483 m, 1386 w, 1328 s, 1256 m, 1207 w, 1169 m, 1209 w, 1125 s, 1081 w, 907 w, 850 w, 825 m, 791 w, 755 m, 683 w; MS *m/z* (relative intensity, %) 410 (16), 266 (49), 238 (62), 237 (20), 225 (26), 210 (13), 199 (15), 171 (13), 151 (15), 145 (20), 144 (100), 130 (20), 129 (14), 128 (10), 116 (10), 67 (27); HRMS Calcd for $C_{24}H_{21}F_3N_2O$: 410.1606; Found: 410.1605.

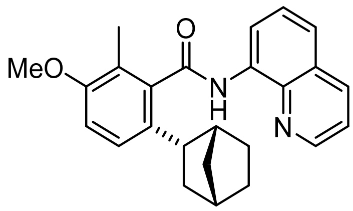

2-(Bicyclo[2.2.1]heptan-2-yl)-5-methoxy-6-methyl-*N*-(quinolin-8-yl)benzamide (2i)

80% yield. endo : exo = 24.7 : 1. R_f 0.26 (hexane/EtOAc = 5/1). Colorless oil. ^{1}H NMR ($CDCl_3$, 400 MHz) (endo) δ 1.30-1.65 (m, 7H), 1.92 (brs, 1H), 2.35 (m, 5H), 3.39 (s, 1 H), 3.85 (s, 3H), 6.91 (d, *J* = 8.7 Hz, 1H), 7.18 (d, *J* = 8.7 Hz, 1H), 7.43 (dd, *J* = 4.1 Hz, 1H), 7.59 (m, 2H), 8.17 (dd, *J* = 8.2, 1.4 Hz, 1H), 8.72 (dd, *J* = 7.3, 1.4 Hz, 1H), 8.99 (dd, *J* = 7.3, 1.4 Hz, 1H), 9.90 (brs, 1H), (exo) 2.89 (t, *J* = 7.8 Hz, 1H); ^{13}C NMR ($CDCl_3$, 100 MHz) δ (endo) 13.12, 23.14, 30.01, 35.85, 37.74, 41.48, 43.35, 55.77, 110.26, 166.92, 121.78, 122.01, 123.31, 125.88, 127.55, 128.15, 131.55, 134.55, 136.42, 138.66, 140.29, 148.39, 155.93, 169.18; IR (neat) 3345 w, 2952 w, 2871 w, 1722 w, 1676 m, 1580 w, 1520 s, 1481 s, 1425 m, 1385 m, 1325 m, 1304 m, 1267 m, 1209 m, 1156 w, 1129 m, 1096 m, 1044 w, 911 w, 853 w, 826 m, 791 m, 756 s, 687 w; MS *m/z* (relative intensity, %) 386 (17), 243 (35), 242 (100), 214 (12), 175 (9), 144 (9); HRMS Calcd for $C_{25}H_{26}N_2O_2$:386.1994; Found: 389.1992.

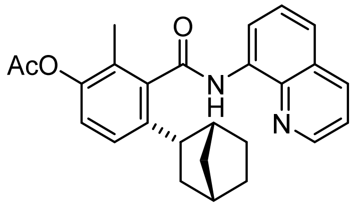

2-(Bicyclo[2.2.1]heptan-2-yl)-5-acetoxy-6-methyl-*N*-(quinolin-8-yl)benzamide(2j)

65% yield. endo : exo = 16.3 : 1. R_f 0.11 (hexane/EtOAc = 10/1). Colorless oil. ^{1}H NMR ($CDCl_3$, 400 MHz) (endo) δ 1.26-1.67 (m, 7H), 1.93 (s, 1H), 2.23 (brs, 3H), 2.25 (t, *J* = 4.1 Hz, 1H), 2.34 (s, 3H), 2.43 (m, 1H), 3.41 (brs, 1H), 7.09 (d, *J* = 8.2 Hz, 1H), 7.23 (d, *J* = 8.2 Hz, 1H), 7.44 (dd, *J* = 4.1 Hz, 1H), 7.59 (m, 2H), 8.17 (dd, *J* = 8.2, 1.4 Hz, 1H), 8.75 (dd, *J* = 2.0 Hz, 1H), 8.97 (dd, *J* = 7.3, 1.8 Hz, 1H), 9.94 (brs, 1H), (exo) 1.82 (tt, *J* = 9.8, 2.6 Hz, 1H), 2.30 (brs, 1H), 2.48 (brs, 1H), 3.21 (dd, *J* = 9.2, 6.0 Hz); ^{13}C NMR ($CDCl_3$, 100 MHz) δ (endo) 13.40, 20.99, 23.18, 29.94, 35.82, 37.74, 41.54, 43.52, 117.00, 121.86, 122.13, 122.21, 126.18, 126.87, 127.47, 128.13, 134.42, 136.39, 137.97, 138.64, 140.53, 147.48, 148.51, 168.30, 169.52; IR (neat) 3342 w, 2953 w, 2872 w, 1762 m, 1722 w, 1676 m, 1520 s, 1481 m, 1425 m, 1383 m, 1371 m, 1325 m, 1304 m, 1261 w, 1203 s, 1167 m, 1145 m, 1132 m, 1084 w, 1063 w, 1015 w, 899 w, 854 w, 827 m, 792 m, 754 s, 668 w; MS *m/z* (relative intensity, %) 414 (20), 271 (34), 270 (100), 229 (28), 228 (33), 161 (10), 144 (17); HRMS Calcd for $C_{26}H_{26}N_2O_2$: 414.1943; Found: 414.1943.

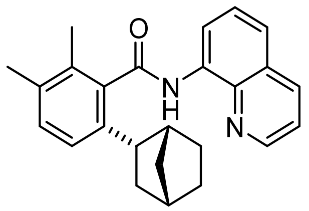

2-(Bicyclo[2.2.1]heptan-2-yl)-5,6-dimethyl-*N*-(quinolin-8-yl)benzamide (2k)

63% yield. endo : exo = 14.3 : 1. R_f 0.14 (hexane/EtOAc = 20/1). Colorless oil. ^{1}H NMR ($CDCl_3$, 400 MHz) (endo) δ 1.26-1.64 (m, 7H), 1.91 (s, 1H), 2.25-2.26 (m, 1H), 2.30 (s, 6H), 2.34-2.49 (m, 1H), [3.36, 3.44 (two broad singlet, 1 H)], 7.13 (d, *J* = 7.8 Hz, 1H), 7.21 (d, *J* = 7.8 Hz, 1H), 7.44 (dd, *J* = 4.1 Hz, 1H), 7.60 (m, 2H), 8.17 (dd, *J* = 8.2, 1.4 Hz, 1H), 8.72 (dd, *J* = 2.0 Hz, 1H), 9.01 (dd, *J* = 7.3, 1.4 Hz, 1H), [9.89, 9.91 (two broad singlet, 1H)], (exo) 2.90 (t, *J* = 6.2 Hz, 1H); ^{13}C NMR ($CDCl_3$, 100 MHz) δ (endo) 16.79, 20.01, 23.23, 29.99, [35.43, 35.97 (two broad peaks)], 37.76, 41.60, [43.60, 43.80 (two broad peaks)], 116.94, 121.78, 121.96, [124.66, 125.14 (two broad peaks)], 127.60, 128.18, 129.92, 132.88, 134.53, 134.83, 136.44, 137.39, 138.69, 139.47, 148.38, 169.96; IR (neat) 3349 w, 2979 w, 2952 w, 2872 w, 1738 s, 1677 m, 1519 m, 1481 m, 1424 w, 1374 m, 1323 m, 1238 w, 1125 w, 1045 w, 938 w, 912 w, 826 w, 791 w, 759 w, 684 w; MS *m/z* (relative intensity, %) 227 (63), 226 (100), 185 (12), 161 (16), 160 (11), 159 (25), 145 (11), 144 (29), 129 (13), 128 (14), 119 (26), 116 (14), 115 (14), 91 (16), 67 (39); HRMS Calcd for $C_{25}H_{26}N_2O$: 370.2045; Found:370.2048.

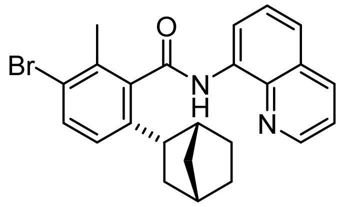

2-(Bicyclo[2.2.1]heptan-2-yl)-5-bromo-6-methyl-*N*-(quinolin-8-yl)benzamide (2l)

84% yield. endo : exo = 13.8 : 1. R_f 0.26 (hexane/EtOAc = 20/1). Colorless oil. ^{1}H NMR ($CDCl_3$, 400 MHz) (endo) δ 1.25-1.60 (m, 7H), 1.93 (t, *J* = 11.2 Hz, 1H), 2.26 (brs, 1H), 2.34-2.48 (m, 4H), [3.33, 3.41 (two broad singlet, 1H)], 7.16 (d, *J* = 8.2 Hz, 1H), 7.41 (d, *J* = 8.7 Hz, 1H), 7.45 (dd, *J* = 4.1 Hz, 1H), 7.59 (dd, *J* = 8.5, 1.6 Hz, 1H), 7.62 (t, *J* =7.8 Hz, 1H), 8.19 (dd, *J* = 8.2, 1.4 Hz, 1H), 8.74 (dd, *J* = 1.8 Hz, 1H), 8.97 (dd, *J* = 6.9, 1.8 Hz, 1H), [9.87, 9.91 (two broad singlet, 1H)], (exo) 2.89 (brs, 1H); ^{13}C NMR ($CDCl_3$, 100 MHz) δ (endo) 20.28, 23.01, 29.73, [35.33, 35.88 (two broad peaks)], 37.53, 37.66, 41.43, [43.07, 43.59 (two broad peaks)], 116.91, 121.76, 122.17, 123.03, [126.37, 126.80 (two broad peaks)], 127.38, 128.02, 132.39, 134.01, 136.36, 138.46, [139.36, 139.54 (two broad peaks)], 140.49, 148.39, 168.11; IR (neat) 3341 w, 2953 m, 2872 w, 1675 m, 1521 s, 1483 m, 1424 m, 1385 m, 1325 m, 1262 w, 1176 w, 1115 w, 903 w, 844 m, 827 m, 791 m, 757 m,

698 w; MS *m/z* (relative intensity, %) 436 (21), 434 (22), 293 (28), 292 (100), 291 (29), 290 (100), 249 (12), 225 (15), 223 (13), 194 (13), 144 (31), 116 (13), 115 (11); HRMS Calcd for $C_{24}H_{23}BrN_2O$: 434.0994; Found: 434.0995.

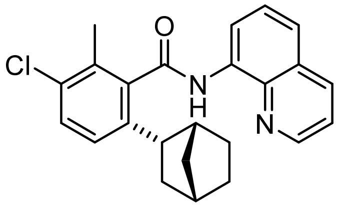

2-(Bicyclo[2.2.1]heptan-2-yl)-5-chloro-6-methyl-*N*-(quinolin-8-yl)benzamide (2m)

77% yield. endo : exo = 15.1 : 1. R_f 0.23 (hexane/EtOAc = 20/1). Colorless oil. ^{1}H NMR ($CDCl_3$, 400 MHz) (endo) δ 1.26-1.61 (m, 7H), 1.93 (brs, 1H), 2.26 (brs, 1H), 2.36-2.49 (m, 4H), [3.35,3.44 (two broad singlet, 1H)], 7.07 (d, *J* = 8.7 Hz, 1H), 7.45 (dd, *J* = 4.3 Hz, 1H), 7.59 (m, 3H), 8.18 (dd, *J* = 8.2, 1.4 Hz, 1H), 8.74 (dd, *J* = 2.0 Hz, 1H), 8.96 (dd, *J* = 7.3, 1.8 Hz, 1H), [9.88, 9.91 (two broad singlet, 1H)], (exo) 2.87 (brs, 1H); ^{13}C NMR ($CDCl_3$, 100 MHz) δ (endo) 17.39, 23.10, 29.84, [35.41, 35.50 (two broad peaks)], 37.72, 41.54, [43.21, 43.73 (two broad peaks)], 117.01, 121.85, 122.26, [126.13, 126.56 (two broad peaks)], 127.50, 128.14, 129.71, 132.57, [134.13, 134.25 (two broad peaks)], 136.46, 138.58, 138.73, 140.62, 148.48, 168.24; IR (neat) 3341 w, 2953 m, 2872 w, 2363 w, 1676 m, 1522 s, 1424 w, 1386 w, 1325 m, 1263 w, 1117 w, 904 w, 826 m, 792 w, 758 m, 688 w; MS *m/z* (relative intensity, %) 390 (22), 248 (34), 247 (28), 246 (100), 218 (10), 205 (13), 179 (15), 144 (21), 67 (11); HRMS Calcd for $C_{24}H_{23}ClN_2O$: 390.1499; Found: 390.1501.

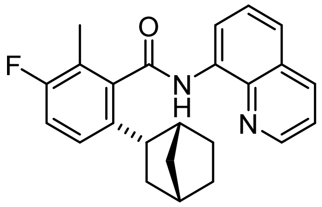

2-(Bicyclo[2.2.1]heptan-2-yl)-5-fluoro-6-methyl-*N*-(quinolin-8-yl)benzamide (2n)

93% yield. endo : exo = 15.6 : 1. R_f 0.14 (hexane/EtOAc = 20/1). Colorless oil. ^{1}H NMR ($CDCl_3$, 400 MHz) (endo) δ 1.19-1.61 (m, 7H), 1.93 (t, *J* = 12.1 Hz, 1H), 2.26 (brs, 1H), 2.31-2.35 (m, 3H), 2.42 (brs, 1H), 3.41 (brs, 1H), 7.08 (t, *J* = 8.9 Hz, 1H), 7.17 (dd, *J* = 8.5, 5.3 Hz, 1H), 7.45 (dd, *J* = 4.1 Hz, 1H), 7.58 (dd, *J* = 8.2, 1.8 Hz, 1H), 7.62 (t, *J* = 7.8 Hz, 1H), 8.19 (dd, *J* = 8.2, 1.8 Hz, 1H), 8.75 (dd, *J* = 1.8 Hz, 1H), 8.97 (dd, *J* = 7.3, 1.8 Hz, 1H), 9.90 (brs, 1H), (exo) 2.90 (dd, *J* = 8.0, 6.6, 1H); ^{13}C NMR ($CDCl_3$, 100 MHz) δ (endo) 11.77 (d, *J* = 4 Hz), 23.12, 29.94, 35.83, 37.74, 41.51, [43.19, 43.46 (two broad peaks)], 115.08 (d, *J* = 22 Hz), 117.06, 121.86 (d, *J* = 18 Hz), 121.89, 122.30, 126.41, 127.55, 128.19, 134.33, 135.58, 136.51, 138.65, 140.76, 159.56 (d, *J* = 243 Hz),

167.99 (exo) 28.51, 30.73, 36.61, 36.93, 40.37, 116.79; IR (neat) 3343 w, 2952 w, 2872 w, 2361 w, 1675 m, 1520 s, 1481 s, 1424 m, 1385 m, 1325 m, 1268 m, 1250 w, 1085 w, 915 w, 824 m, 753 s, 693 m, 667 w; MS *m/z* (relative intensity, %) 418 (19), 276 (11), 275 (62), 274 (100), 207 (12), 179 (10), 178 (15), 165 (11), 144 (13); HRMS Calcd for $C_{24}H_{23}FN_2O$: 374.1794; Found: 374.1790.

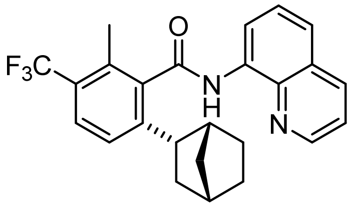

2-(Bicyclo[2.2.1]heptan-2-yl)-5-trifluoromethyl-6-methyl-*N*-(quinolin-8-yl)benzamide (2o)

87% yield. endo : exo = 9.5 : 1. R_f 0.21 (hexane/EtOAc = 20/1). Colorless oil. ^{1}H NMR ($CDCl_3$, 400 MHz) (endo) δ 1.03-1.61 (m, 7H), 1.95 (m, 1H), 2.29 (brs, 1H), 2.36-2.52 (m, 4H), 3.46 (two triplet peaks, *J* = 5.4 Hz, 1H), 7.31 (d, *J* = 8.2 Hz, 1H), 7.46 (dd, *J* = 4.3 Hz, 1H), 7.64 (m, 1H), 8.20 (d, *J* = 8.2 Hz, 1H), 8.75 (dd, *J* = 1.8 Hz, 1H), 8.98 (dd, *J* = 6.9, 1.8 Hz, 1H), 8.98 (dd, *J* = 1.8 Hz, 1H), 9.92 (two singlet peaks, 1H), (exo) 2.95 (brs, 1H); ^{13}C NMR ($CDCl_3$, 150 MHz) δ 16.24, 23.25, 23.23, 28.45, 28.85, 35.39, 36.02, 37.70, 37.86, 41.64, 41.74, 43.33, 43.54, 43.85, 44.22, 117.06, 117.17, 121.91, 122.40, 122.51, 123.71, 124.94, 125.39, 125.53, 126.00, 127.24, 127.34, 127.44, 127.52, 127.67, 127.83, 127.99, 128.21, 128.57, 128.72, 133.47, 133.64, 134.10, 134.22, 134.37, 136.53, 137.68, 138.64, 141.28, 144.38, 144.56, 148.55, 168.11, 168.29; IR (neat) 3341 w, 2954 w, 2873 w, 1676 m, 1597 w, 1521 s, 1483 m, 1424 m, 1386 m, 1320 s, 1261 w, 1212 m, 1173 m, 1121 s, 1009 w, 907 w, 825 m, 791 m, 754 s, 667 w; MS *m/z* (relative intensity, %) 425 (10), 424 (35), 281 (29), 280 (100), 252 (13), 239 (14), 213 (18), 144 (22), 67 (10); HRMS Calcd for $C_{25}H_{23}F_3N_2O$: 424.1762; Found: 424.1761.

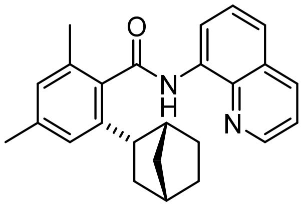

2-(Bicyclo[2.2.1]heptan-2-yl)-4,6-dimethyl-*N*-(quinolin-8-yl)benzamide (2p)

67% yield. endo : exo = 13.7 : 1. R_f 0.19 (hexane/EtOAc = 20/1). Colorless oil. ^{1}H NMR ($CDCl_3$, 400 MHz) (endo) δ 1.24-1.63 (m, 7H), 1.91 (tt, *J* = 12.1, 3.7 Hz, 1H), 2.25 (brs, 1H), 2.36-2.45 (m, 7H), 3.44 (t, *J* = 5.7 Hz, 1H), 6.93 (d, *J* = 11.9 Hz, 1H), 7.02 (d, *J* = 10.1 Hz, 1H), 7.43 (dd, *J* = 4.1 Hz, 1H), 7.55 (dd, *J* = 8.2, 1.4 Hz, 1H), 7.61 (t, *J* = 7.8 HZ, 1H), 8.17 (dd, *J* = 8.2, 1.4 Hz, 1H), 8.72 (dd, *J* = 2.0 Hz, 1H), 8.98 (dd, *J* = 7.3, 1.4 Hz, 1H), 9.89 (brs, 1H), (exo) 2.94 (t, *J* = 7.3 Hz, 1H);

^{13}C NMR ($CDCl_3$, 100 MHz) δ (endo) 19.60, 21.76, 23.21, 30.03, 35.56, 37.77, 41.55, 43.42, 43.58, 116.88, 121.77, 121.93, 125.64, 127.59, 128.17, 128.65, 134.51, 134.70, 136.42, 136.65, 138.09, 138.70, 140.09, 148.38, 169.75; IR (neat) 3345 w, 2953 m, 2871 w, 1674 m, 1521 s, 1482 m, 1424 m, 1384 m, 1325 m, 1252 w, 1177 w, 1110 w, 1076 w, 916 w, 890 w, 844 m, 828 m, 791 m, 758 m, 699 w; MS *m/z* (relative intensity, %) 370 (24), 227 (86), 226 (100), 159 (13), 144 (14); HRMS Calcd for $C_{25}H_{26}N_2O$: 370.2045; Found:370.2049.

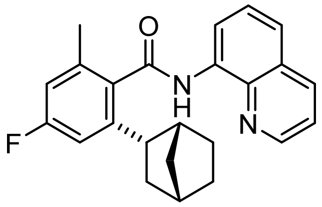

2-(Bicyclo[2.2.1]heptan-2-yl)-6-dimethyl-4-fluoro-*N*-(quinolin-8-yl)benzamide (2q)

61% yield. endo : exo = 11.7 : 1. R_f 0.17 (hexane/EtOAc = 20/1). Colorless oil. ^1H NMR ($CDCl_3$, 400 MHz) (endo) δ 1.01-1.55 (m, 5H), 1.49-1.55 (m, 1H), 1.86 (t, *J* = 11.9 Hz, 1H), 2.19 (brs, 1H), 2.34-2.39 (m, 4H), 3.38 (d, *J* = 10.5 Hz, 1H), 6.76 (d, *J* = 9.2 Hz, 1H), 6.83 (d, *J* = 10.5 Hz, 1H), 7.38 (dd, *J* = 4.1 Hz, 1H), 7.53 (m, 1H), 8.11 (d, *J* = 8.2 HZ, 1H), 8.67 (t, *J* = 2.1 Hz, 1H), 8.89 (d, *J* = 7.3 Hz, 1H), 9.81 (brs, 1H), (exo) 2.88 (t, *J* = 7.6 Hz, 1H); ^{13}C NMR ($CDCl_3$, 100 MHz) δ (endo) 19.78, 23.17, 29.88, 35.79, 37.69, 41.5, [43.50, 43.69 (two broad peaks)], 112.02 (d, *J* = 22 Hz), 144.59 (d, *J* = 21 Hz), 117.00, 121.87, 122.21, 127.57, 128.18, 134.43, 135.31 (d, *J* = 3 Hz), 136.51, 137.32 (d, *J* = 9 Hz), 138.64, 143.49 (d, *J* = 8 Hz), 148.51, 162.65 (d, *J* =245 Hz), 168.73 (exo) 25.65, 26.12, 28.43, 30.69, 36.71, 36.89, 40.36, 44.27; IR (neat) 3347 w, 2953 w, 2872 w, 1598 m, 1521 s, 1483 m, 1424 w, 1385 w, 1325 m, 1301 w, 1261 w, 1141 w, 983 w, 849 w, 826 w, 792 w, 759 w, 668 m; MS *m/z* (relative intensity, %) 374 (21), 231 (53), 230 (100), 189 (10), 163 (17), 146 (10), 144 (14), 67 (10); HRMS Calcd for $C_{24}H_{23}FN_2O$: 374.1794; Found:374.1792.

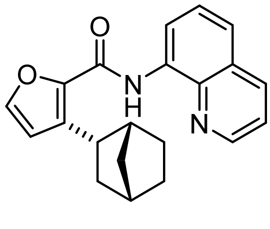

3-(Bicyclo[2.2.1]heptan-2-yl)-*N*-(quinolin-8-yl)furan-2-carboxamide (2r)

64% yield. endo : exo = 0 : 1. R_f 0.11 (hexane/EtOAc = 20/1). Colorless oil. ^1H NMR ($CDCl_3$, 400 MHz) (exo) δ 1.26-1.39 (m, 2H), 1.49-1.66 (m, 5H), 1.97 (m, 1H), 2.33 (brs, 1H), 2.36 (brs, 1H), 3.59 (dd, *J* = 8.7, 5.5 Hz, 1H), 6.53 (s, 1H), 7.51 (m, 4H), 8.16 (d, *J* = 8.2 Hz, 1H), 8.88 (m, 2H), 10.72 (brs, 1H); ^{13}C NMR ($CDCl_3$, 100 MHz) δ (exo) 28.79, 30.42, 36.83, 36.99, 37.74, 39.58,

43.40, 112.30, 116.54, 121.54, 121.73, 127.52, 128.19, 134.70, 136.44, 138.89, 139.53, 141.63, 143.05, 148.42, 157.82; IR (neat) 3341 w, 2952 w, 2872 w, 1668 m, 1591 w, 1576 w, 1525 s, 1483 m, 1456 w, 1425 w, 1384 w, 1328 m, 1261 w, 1252 w, 1175 w, 1071 w, 873 m, 844 m, 826 w, 750 m, 697 w; MS *m/z* (relative intensity, %) 333 (11), 332 (43), 287 (14), 265 (13), 209 (41), 188 (14), 172 (13), 171 (100), 160 (45), 159 (23), 145 (16), 144 (75), 130 (11), 129 (12), 128 (11), 121 (34), 117 (13), 116 (16), 109 (12), 95 (11), 91 (23), 81 (12), 79 (18), 77 (19), 67 (20), 65 (19); HRMS Calcd for $C_{21}H_{20}N_2O_2$: 332.1525; Found:332.1527.

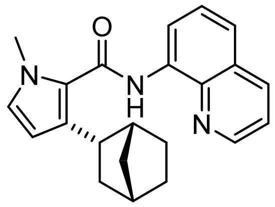

3-(Bicyclo[2.2.1]heptan-2-yl)-1-methyl-*N*-(quinolin-8-yl)-1H-pyrrole-2-carboxamide (2s)
57% yield. endo : exo = 1 : 1.1. R_f 0.26 (hexane/EtOAc = 5/1). Colorless oil. ^{1}H NMR ($CDCl_3$, 400 MHz) (endo) δ 1.17-1.71 (m, 7H), 2.02 (m, 1H), 2.30 (d, *J* = 14.2 Hz, 1H), 2.79 (brs, 1H), 3.75 (m, 1H), 3.93 (d, *J* = 10.1 Hz, 1H), 6.11 (dd, *J* = 11.2, 2.5 Hz, 1H), 6.70 (dd, *J* = 17.2, 2.5 Hz, 1H), 7.43 (dd, *J* = 4.1 Hz, 1H), 7.49 (dd, *J* = 8.1, 1.5 Hz, 1H), 7.56 (t, *J* = 7.8 Hz, 1H), 8.15 (dd, *J* = 8.2, 1.8 Hz, 1H), 8.79 (td, *J* = 3.0, 1.2 Hz, 1H), 8.88 (m, 1H), 10.25 (brs, 1H), (exo) 2.53 (d, *J* =3.2 Hz, 1H), 3.26 (dd, *J* = 8.7, 5.5 Hz, 1H); ^{13}C NMR ($CDCl_3$, 100 MHz) δ 23.40, 28.94, 30.40, 36.89, 37.13, 37.30, 37.56, 38.86, 40.11, 40.84, 40.90, 43.13, 43.89, 10 6.26, 108.01, 116.30, 116.39, 121.18, 121.68, 121.70, 123.73, 125.07, 126.78 126.93, 127.60, 127.6 2, 128.15, 130.77, 135.24, 135.45, 136.33, 138.87, 148.02, 148.06, 161.08, 161.14; IR (neat) 3359 w, 2951 w, 2871 w, 2361 w, 1722 w, 1652 m, 1521 s, 1482 m, 1423 m, 1382 m, 1326 m, 1303 m, 1259 m, 1207 m, 1123 m, 1062 w, 1016 w, 884 w, 855 w, 824 w, 790 w, 753 m, 665 w; MS *m/z* (relative intensity, %) 354 (21), 202 (13), 201 (10), 174 (100), 171 (12), 122 (18), 94 (14); HRMS Calcd for $C_{22}H_{23}N_3O$: 345.1845; Found:345.1843.

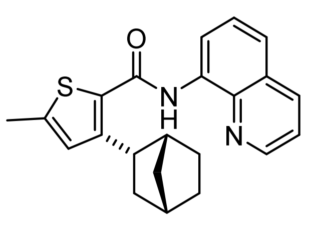

3-(Bicyclo[2.2.1]heptan-2-yl)-5-methyl-*N*-(quinolin-8-yl)thiophene-2-carboxamide (2t)
72% yield. endo : exo = 1.2 : 1. R_f 0.19 (hexane/EtOAc = 20/1). Colorless oil. ^{1}H NMR ($CDCl_3$, 400 MHz) (endo) δ 1.20-1.72 (m, 7H), 2.04 (tt, *J* = 12.1, 3.9 Hz, 1H), 2.35 (brs, 1H), 2.47 (brs, 1H), 2.53

(s, 3H), 3.53 (dd, *J* = 9.2, 5.5 Hz, 1H), 6.81 (d, *J* = 11.9 Hz, 1H), 7.45 (dd, *J* = 4.3 Hz, 1H), 7,50 (d, *J* = 8.2 Hz, 1H), 8.16 (dd, *J* = 8.2, 1.4 Hz, 1H), 8.82 (d, *J* = 4.1 Hz, 1H), 8.84 (d, *J* =7.3 Hz, 1H), 10.37 (brs, 1H), (exo) 2.17 (m, 1H), 2.51 (s, 3H), 2.77 (brs, 1H), 3.53 (dd, *J* = 9.2, 5.5 Hz, 1H); ^{13}C NMR ($CDCl_3$, 100 MHz) δ 15.88, 23.82, 28.85, 30.24, 30.68, 36.77, 36.93, 37.39, 37.81, 40.52, 40.86, 41.00, 42.12, 43.24, 43.29, 116.57, 116.65, 121.46, 121.73, 127.20, 127.62, 128.11, 128.51, 129.53, 131.05, 135.08, 135.14, 136.45, 138.77, 142.01, 142.90, 148.19, 148.26, 149.09, 152.40, 161.47; IR (neat) 3356 w, 2952 w, 2871 w, 1643 w, 1521 s, 1483 m, 1445 w, 1424 m, 1383 m, 1327 m, 1259 w, 1221 w, 1176 w, 1107 w, 876 w, 825 m, 790 m, 756 m, 698 w, 664 w; MS *m/z* (relative intensity, %) 362 (22), 219 (32), 218 (100), 190 (22), 177 (12), 171 (30), 151 (10), 144 (10), 111 (14); HRMS Calcd for $C_{22}H_{22}N_2OS$: 362.1453; Found:362.1454.

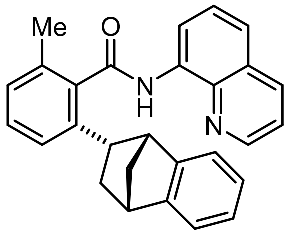

2-Methyl-N-(quinolin-8-yl)-6-(1,2,3,4-tetrahydro-1,4-methanonaphthalen-2-yl)benzamide (8)
84% yield. endo : exo = 5.0 : 1. R_f 0.17 (hexane/EtOAc = 10/1). Colorless oil. ^{1}H NMR ($CDCl_3$, 400 MHz) δ 1.19, 1.21, 1.22, 1.23, 1.25, 1.26, 1.27, 1.32, 1.33, 1.34, 1.35, 1.36, 1.37, 1.39, 1.61, 1.72, 1.74, 1.77, 1.79, 1.82, 2.01, 2.02, 2.06, 2.07, 2.08, 2.09, 2.10. 2.11, 2.12, 2.24, 2.40, 2.43, 2.44, 2.45, 2.93, 2.95, [(exo) 3.08, (endo) 3.79, 1H], 3.34, 3.37, 3.39, 3.40, 3.52, 4.00, 4.01, 4.83, 4.84, 4.85, 5.26, 5.28, 6.81, 6.83, 6.84, 6.86, 6.87, 6.92, 6.94, 6.96, 6.97, 6.99, 7.01, 7.05, 7.07, 7.10, 7.11, 7.12, 7.13, 7.14, 7.15, 7.16, 7.18, 7.22, 7.23, 7.24, 7.27, 7.28, 7.33, 7.35, 7.36, 7.37, 7.38, 7.43, 7.44, 7.45, 7.46, 7.47, 7.48, 7.55, 7.56, 7.57, 7.58, 7.59, 7.60, 7.61, 7.63, 7.65, 7.67, 8.17, 8.19, 8.21, 8.73, 8.74, 8.76, 8.77, 8.89, 8.90, 8.91, 9.03, 9.05, [(exo) 9.95, (endo) 10.02, 1H]; ^{13}C NMR ($CDCl_3$, 100 MHz) δ 19.62, 27.26, 33.67, 36.41, 38.89, 42.12, 42.68, 44.42, 44.86, 46.75, 48.43, 51.55, 52.12, 52.70, 76.51, 76.95, 77.16, 77.37, 85.11, 117.00, 120.50, 120.76, 121.79, 121.88, 121.97, 122.03, 122.13, 123.12, 123.48, 124.41, 124.77, 125.19, 125.65, 126.33, 126.69, 127.38, 127.61, 127.81, 128.00, 128.10, 128.16, 128.24, 133.62, 134.46, 134.62, 136.51, 138.66, 138.73, 144.27, 145.37, 148.42, 148.53, 148.61, 169.54, 177.56; IR (neat) 3345 w, 2968 w, 2872 2, 1719 w, 1675 m, 1595 w, 1580 w, 1519 s, 1481 s, 1424 m, 1385 m, 1325 m, 1264 w, 1156 w, 1132 w, 899 w, 826 w, 791 m, 752 s, 698 w; MS *m/z* (relative intensity, %) 406 (M^++2, 17), 405 (M^++1, 52), 404 (M^+, 13), 307 (19), 289 (20), 287 (11), 262 (10), 261 (48), 260 (13), 159 (12), 155 (25), 154 (100), 145 (54), 144 (16), 139 (12), 138 (27), 137 (49), 136 (69), 129 (36), 128 (13), 120 (10), 117 (10), 116 (18), 115 (25), 107 (20), 91 (17), 90 (14), 89 (21), 78 (10), 77 (22), 57 (22); HRMS Calcd for $C_{28}H_{24}N_2O$: 404.1889; Found(CI^+):405.1967.

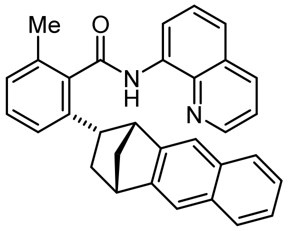

2-Methyl-N-(quinolin-8-yl)-6-(1,2,3,4-tetrahydro-1,4-methanoanthracen-2-yl)benzamide (9)

92% yield. endo : exo = 2.6 : 1. R_f 0.11 (hexane/EtOAc = 3/1). Colorless oil. ^{1}H NMR ($CDCl_3$, 400 MHz δ 0.87, 0.88, 0.89, 0.90, 0.92, 1.25, 1.26, 1.47, 1.48, 1.49, 1.50, 1.82, 1.84, 1.85, 1.87, 1.89, 2.16, 2.18, 2.19, 2.20, 2.21, 2.22, 2.41, 2.45, 2.48, 2.50, 2.51, 2.52, [(exo) 3.17, (endo) 3.89, 1H], 3.51, 3.52, 3.63, 3.66, 5.35, 5.37, 6.65, 6.66, 6.68, 6.93, 6.94, 7.17, 7.18, 7.25, 7.27, 7.28, 7.35, 7.37, 7.38, 7.40, 7.41, 7.42, 7.43, 7.44, 7.45, 7.46, 7.54, 7.55, 7.78, 7.60, 7.62, 7.64, 7.65, 7.66, 7.67, 7.80, 7.82, 8.17, 8.18, 8.19, 8.74, 8.79, 8.89, 8.90, 9.06, 9.07, [(exo) 9.98, (endo) 10.04, 1H]; ^{13}C NMR ($CDCl_3$, 100 MHz) δ 19.63, 36.71, 36.88, 42.58, 43.24, 44.16, 44.56, 45.63, 50.66, 51.85, 76.95, 77.16, 77.37, 117.02, 117.06, 118.08, 118.36, 118.52, 121.81, 121.88, 122.03, 122.15, 122.35, 123.48, 124.76, 124.85, 125.02, 127.60, 127.63, 127.77, 127.88, 127.99, 128.05, 128.20, 128.25, 129.21, 132.60, 132.79, 132.82, 133.20, 133.67, 134.44, 134.62, 134.84, 136.49, 136.51, 138.62, 138.66, 138.73, 138.86, 139.90, 142.95, 147.04, 147.33, 147.63, 148.46, 148.55, 169.02, 169.50; IR (neat) 3344 w, 3052 w, 3012 w, 2966 w, 2871 w, 1673 m, 1595 w, 1579 w, 1517 s, 1579 w, 1517 s, 1481 s, 1423 m, 1385 m, 1325 m, 1263 w, 1217 w, 1127 w, 899 w, 881 w, 826 w, 790 m, 750 w, 693 w; MS *m/z* (relative intensity, %) 455 (M^++1, 11), 454 (M^+, 31), 311 (17), 310 (57), 289 (27), 288 (12), 287 (51), 167 (12), 166 (82), 165 (75), 146 (11), 145 (100), 144 (71), 132 (21), 115 (10); HRMS Calcd for $C_{32}H_{26}N_2O$: 454.2045; Found:454.2049.

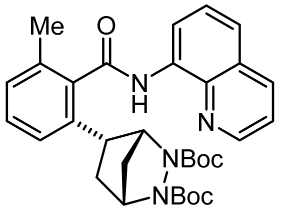

Di-tert-butyl 5-(3-methyl-2-(quinolin-8-ylcarbamoyl)phenyl)-2,3-diazabicyclo[2.2.1]heptane-2,3-dicarboxylate (10a)

84% yield. endo : exo = 6.2 : 1. R_f 0.14 (hexane/EtOAc = 3/1). Colorless oil. ^{1}H NMR ($CDCl_3$, 400 MHz) δ (endo) 1.10-1.49 (m, 18H), 1.71 (d, J = 10.1 Hz, 1H), 1.80 (d, J = 10.1 Hz, 1H), 2.03 (brs, 1H), 2.15 (brs, 1H), 2.44 (s, 3H), 3.59 (brs, 1H), 4.47 (brs, 1H), 4.89 (brs, 1H), 7.17 (d, J = 7.3 Hz, 1H), 7.32 (s, 1H), 7.47 (dd, J = 4.1 Hz, 1H), 7.64-7.53 (m, 3H), 8.21-8.17 (m, 1H), 8.76 (dd, J = 1.8 Hz, 1H), 8.96 (d, J = 6.0 Hz, 1H), 9.97 (brs, 1H), (exo) 7.08 (d, J = 7.8 Hz, 1H), 9.90 (brs, 1H); ^{13}C

NMR ($CDCl_3$, 100 MHz) δ 28.07, 28.20, 76.80, 77.02, 77.22, 81.08, 116.36, 116.82, 121.49, 121.66, 121.76, 127.10, 127.15, 127.39, 127.73, 128.08, 128.14, 128.29, 128.45, 128.64, 128.67, 134.38, 136.01, 138.32, 140.50, 148.00, 168.41; IR (neat) 3314 w, 3002 w, 2978 w, 2931 w, 1691 m, 1673 m, 1595 w, 1578 w, 1520 s, 1482 m, 1459 m, 1424 w, 1367 s, 1326 s, 1255 m, 1137 s, 910 w, 860 w, 827 w, 756 s, 701 m; MS *m/z* (relative intensity, %) 560 (M^++2, 36), 559 (M^++1, 100), 503 (29), 460 (11), 459 (37), 458 (12), 359 (15), 358 (10), 357 (27), 289 (13), 215 (15), 145 (19); HRMS Calcd for $C_{32}H_{38}N_4O_5$: 558.2842; Found(CI^+): 559.2917.

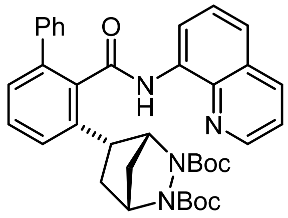

Di-tert-butyl 5-(2-(quinolin-8-ylcarbamoyl)-[1,1'-biphenyl]-3-yl)-2,3-diazabicyclo[2.2.1]heptane-2,3-dicarboxylate (10b)

59% yield. R_f 0.14 (hexane/EtOAc = 3/1). Colorless oil. ^1H NMR ($CDCl_3$, 400 MHz) (endo) δ 1.26-1.64 (m, 18H), 1.77-1.83 (m, 2H), 2.03 (brs, 1H), 2.21-2.56 (c, 1H), 3.72 (m, 1H), 4.50 (m, 1H), 4.92 (m, 1H), 7.07 (dd, J = 7.7 Hz, 1H), 7.19 (t, J = 7.5 Hz, 2H), 7.37-7.34 (m, 2H), 7.44-7.50 (m, 5H), 7.72 (m, 1H), 8.07 (d, J = 8.2 Hz, 1H), 8.59 (brs, 1H), 8.67 (d, J = 5.4 Hz, 1H), 9.62 (brs, 1H); ^{13}C NMR ($CDCl_3$, 100 MHz) δ 15.88, 23.82, 28.85, 30.24, 30.68, 36.77, 36.93, 37.39, 37.81, 40.52, 40.86, 41.00, 42.12, 43.24, 43.29, 116.57, 116.65, 121.46, 121.73, 127.20, 127.62, 128.11, 128.51, 129.53, 131.05, 135.08, 135.14, 136.45, 138.77, 142.01, 142.90, 148.19, 148.26, 149.09, 152.40, 161.47; IR (neat) 3356 w, 2952 w, 2871 w, 1643 w, 1521 s, 1483 m, 1445 w, 1424 m, 1383 m, 1327 m, 1259 w, 1221 w, 1176 w, 1107 w, 876 w, 825 m, 790 m, 756 m, 698 w, 664 w; MS *m/z* (relative intensity, %) 623 (M^++3, 11), 622 (M^++2, 42), 621 (M^++1, 100), 565 (15), 522 (11), 521 (30), 421 (26), 420 (22), 419 (63), 352 (10), 351 (36), 145 (14); HRMS Calcd for $C_{37}H_{40}N_4O_5$: 620.2999; Found(CI^+):621. 3073.

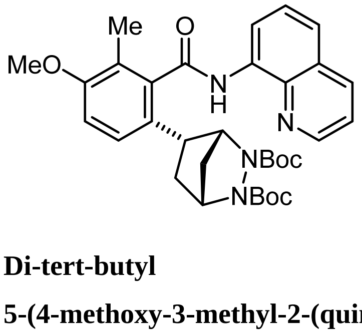

Di-tert-butyl 5-(4-methoxy-3-methyl-2-(quinolin-8-ylcarbamoyl)phenyl)-2,3-diazabicyclo[2.2.1]heptane-2,3-

dicarboxylate (10c)

93% yield. endo : exo = 2.9 : 1. R_f 0.11 (hexane/EtOAc = 3/1). Colorless oil. ^{1}H NMR ($CDCl_3$, 400 MHz) δ 0.89-2.17 (c, 22H), 2.28 (s, 3H), 3.51 (brs, 1H), 3.86 (s, 3H), 4.47 (brs, 1H), 4.82 (brs, 1H), 6.88 (m, 1H), 7.02, 7.03, 7.45 (m, 1H), 7.72-7.52 (m, 3H), 8.16-8.19 (m, 1H), 8.75 (brs, 1H), 8.95 (d, J = 4.4 Hz, 1H), [(exo) 9.89, (emdo) 9.95 (brs, 1H)]; ^{13}C NMR ($CDCl_3$, 100 MHz) δ 13.11, 28.19, 28.32, 28.37, 55.72, 55.76, 76.95, 77.16, 77.37, 81.23, 110.77, 111.20, 116.94, 121.65, 121.88, 122.10, 122.22, 122.86, 126.72, 127.44, 128.16, 134.41, 136.40, 138.69, 139.72, 148.30, 148.51, 156.43, 156.53; IR (neat) 3343 w, 3006 w, 2978 w, 2931 w, 1681 m, 1582 w, 1519 m, 1481 m, 1366 m, 1325 m, 1269 m, 1136 m, 1098 m, 1044 w, 911 w, 826 w, 793 w, 752 s, 665 w; MS *m/z* (relative intensity, %) 590 (M^++2, 36), 589 (M^++1, 100), 534 (12), 533 (35), 490 (26), 489 (81), 488 (15), 433 (14), 390 (12), 389 (45), 388 (16), 387 (39), 319 (32), 245 (13), 145 (12), 144 (10); HRMS Calcd for $C_{33}H_{40}N_4O_6$: 588.2948; Found(CI^+):589.3023.

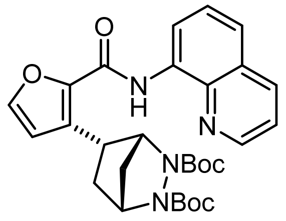

Di-tert-butyl 5-(2-(quinolin-8-ylcarbamoyl)furan-3-yl)-2,3-diazabicyclo[2.2.1]heptane-2,3-dicarboxylate (10d)

64% yield. R_f 0.09 (hexane/EtOAc = 3/1). Colorless oil. ^{1}H NMR ($CDCl_3$, 400 MHz) δ 1.24-1.72 (m, 18H), 1.82 (brs, 2H), 2.63 (brs, 1H), 4.07 (brs, 1H), 4.62 (m, 2H), 6.45 (d, J = 1.6 Hz, 1H), 7.48 (dd, J = 4.1 Hz, 1H), 7.52-7.59 (m, 3H), 8.18 (dd, J = 8.2, 1.4 Hz, 1H), 8.86-8.89 (m, 2H), 10.73 (brs, 1H); ^{13}C NMR ($CDCl_3$, 100 MHz) δ 28.27, 28.39, 36.01, 36.48, 36.93, 37.31, 59.66, 60.29, 62.73, 64.61, 76.85, 77.16, 77.48, 81.45, 81.66, 111.54, 116.50, 121.70, 121.80, 127.53, 128.13, 133.80, 134.48, 136.48, 138.74, 142.53, 143.33, 148.49, 156.81; IR (neat) 3341 w, 3004 w, 2978 w, 2031 w, 1719 m, 1674 m, 1482 m, 1425 w, 1367 m, 1327 m, 1257 m, 1142 m, 1110 m, 874 w, 752 s, 700 w; MS *m/z* (relative intensity, %) 535 (M^++1, 11), 479 (10), 434 (12), 380 (11), 379 (45), 335 (23), 334 (15), 266 (23), 265 (100), 171 (26), 159 (13), 145 (15), 144 (14), 137 (13), 136 (23), 121 (18), 89 (10), 77 (11), 57 (64), 41 (12); HRMS Calcd for $C_{29}H_{34}N_4O_5$: 534.2478; Found(FAB^+):535.2548.

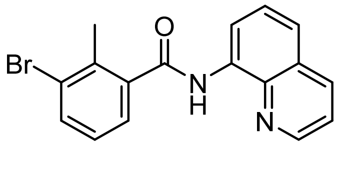

3-Bromo-2-methyl-*N*-(quinolin-8-yl)benzamide (1l)

white solid. mp = 184 °C. Rf 0.19 (Hexane/EtOAc = 20/1). ^{1}H NMR (CDCl$_3$, 400 MHz) δ 2.62 (s, 3H), 7.19 (t, *J* = 7.8 Hz, 1H), 7.47 (dd, *J* = 4.1 Hz, 1H), 7.60 (m, 3H), 7.70 (d, *J* =7.8 Hz, 1H), 8.20 (dd, *J* = 8.2, 1.8 Hz, 1H), 8.93 (dd, *J* = 7.3, 1.4 Hz, 1H), 10.13 (brs, 1H); ^{13}C NMR (CDCl$_3$, 100 MHz) δ 20.44, 116.85, 121.85, 122.28, 126.27, 127.06, 127.46, 127.53, 128.14, 134.46, 134.55, 136.10, 136.56, 138.67, 139.23, 148.52, 167.62; IR (neat) 3353 w, 1676 m, 1523 s, 1484 m, 1424 m, 1260 w, 1208 w, 1176 w, 1073 w, 1006 w, 904 w, 824 w, 790 m, 754 m, 709 w, 680 w, 663 w; MS *m/z* (relative intensity, %) 342 (37), 340 (36), 325 (26), 323 (28), 298 (17), 296 (17), 262 (15), 261 (78), 199 (99), 198 (33), 197 (100), 196 (25), 171 (42), 169 (41), 144 (58), 116 (22), 90 (48), 89 (34); HRMS Calcd for $C_{17}H_{13}BrN_2O$:340.0211; Found: 340.0207.

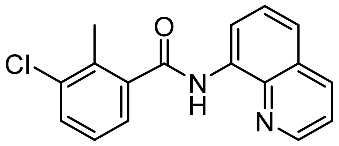

3-Chloro-2-methyl-*N*-(quinolin-8-yl)benzamide (1m)

white solid. mp = 176 °C. Rf 0.19 (Hexane/EtOAc = 20/1). ^{1}H NMR (CDCl$_3$, 400 MHz) δ 2.59 (s, 3H), 7.26 (t, *J* = 7.8 Hz, 1H), 7.46 (m, 1H), 7.52 (dd, *J* = 13.3, 7.8 Hz, 2H), 7.59 (dd, *J* =10.8, 8.5 Hz, 2H), 8.19 (d, *J* = 8.2 Hz, 1H), 8.77 (t, *J* = 2.1 Hz, 1H), 8.93 (d, *J* = 6.9 Hz, 1H), 10.14 (brs, 1H); ^{13}C NMR (CDCl$_3$, 100 MHz) δ 17.29, 116.77, 121.84, 122.19, 125.56, 127.09, 127.48, 128.09, 131.06, 134.47, 134.51, 136.13, 136.49, 138.62, 139.18, 148.46, 167.52; IR (neat) 3357 w, 1678 m, 1528 s, 1485 m, 1425 m, 1387 m, 1328 m, 1260 w, 1211 w, 1011 w, 906 w, 823 w, 789 m, 757 m, 707 w, 666 w; MS *m/z* (relative intensity, %) 298 (12), 296 (36), 279 (25), 261 (39), 252 (14), 171 (13), 155 (32), 154 (16), 153 (100), 152 (23), 144 (40), 127 (16), 125 (49), 116 (10), 89 (21); HRMS Calcd for $C_{17}H_{13}ClN_2O$:296.0716; Found:296.0719.

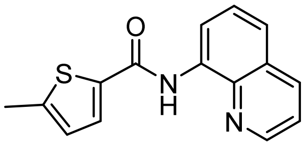

2-methyl-*N*-(quinolin-8-yl)thiophene-5-carboxamide (1t)

white solid. mp = 129 °C. Rf 0.11 (Hexane/EtOAc = 10/1). ^{1}H NMR (CDCl$_3$, 400 MHz) δ 2.58 (s, 3H), 6.84 (dd, *J* = 3.7, 0.9 Hz, 1H), 7.48 (dd, *J* = 8.2, 1.4 Hz, 1H), 7.57 (t, *J* = 8.0 Hz, 2H), 7.66 (d, *J* =3.7, Hz, 1H), 8.18 (dd, *J* = 8.2, 1.4 Hz, 1H), 8.84 (m, 2H), 10.50 (brs, 1H); ^{13}C NMR (CDCl$_3$, 100 MHz) δ 15.98, 166.51, 121.57, 121.81, 126.48, 127.64, 128.12, 129.03, 134.63, 136.51, 137.44, 138.66, 146.52, 148.39, 160.23; MS *m/z* (relative intensity, %) 268 (40), 125 (100); HRMS Calcd for $C_{15}H_{12}N_2OS$:268.0670; Found:268.0671.

Supplementary Table 1: Competition experiments

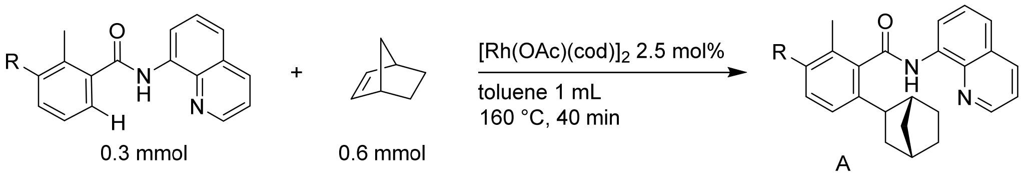

entry	R =	acid	NMR yields SM(amide)	NMR yields product	note
1	OMe	-	52%	41%	50 min
2	CF_3	-	84%	15%	50 min
3	OMe	PivOH 3 equiv.	70%	30%	toluene 0.5 mL
4	CF_3	PivOH 3 equiv.	77%	25%	toluene 0.5 mL
5	OMe	PivOH 1 equiv.	69%	31%	
6	CF_3	PivOH 1 equiv.	68%	32%	

Deuterium Labeling Experiments

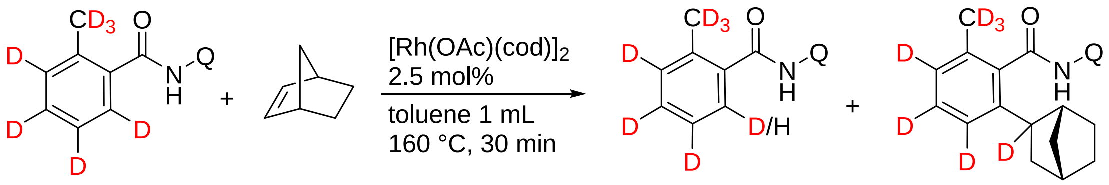

1a-*d*$_7$

To an oven-dried 5 mL screw-capped vial, 2-methyl-N-(8-quinolinyl)benzamide **1a-*d*$_7$** (79 mg, 0.3 mmol), 2-nobbornene (57 mg, 0.6 mmol), $[Rh(OAc)(cod)]_2$ (4.1 mg, 0.0075 mmol), toluene (1.0 mL) were added. The mixture was stirred for 30 min at 160 °C followed by cooling. The mixture was filtered through a celite pad and concentrated in vacuo. The residue was purified by column chromatography on silica gel (eluent: hexane/EtOAc= 50/1) and GPC to afford the desired alkylated product and starting amide.

Experiments without 2-norbornene were also conducted in a similar way.

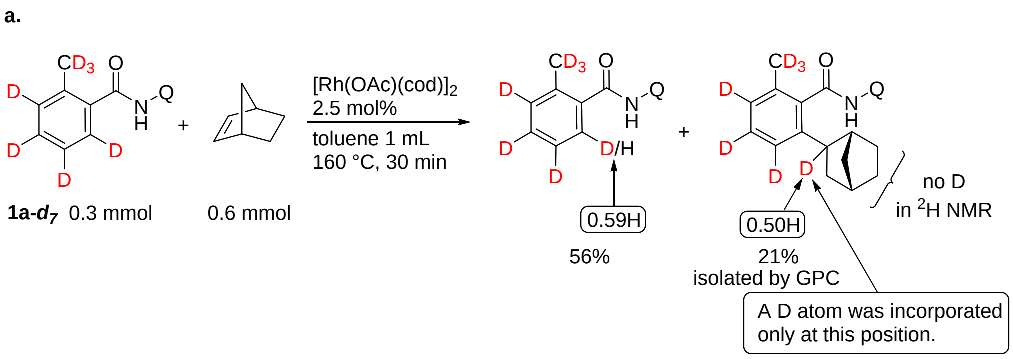

Supplementary Figure 1: Estimation of deuterium and hydrogen incorporation. (a)

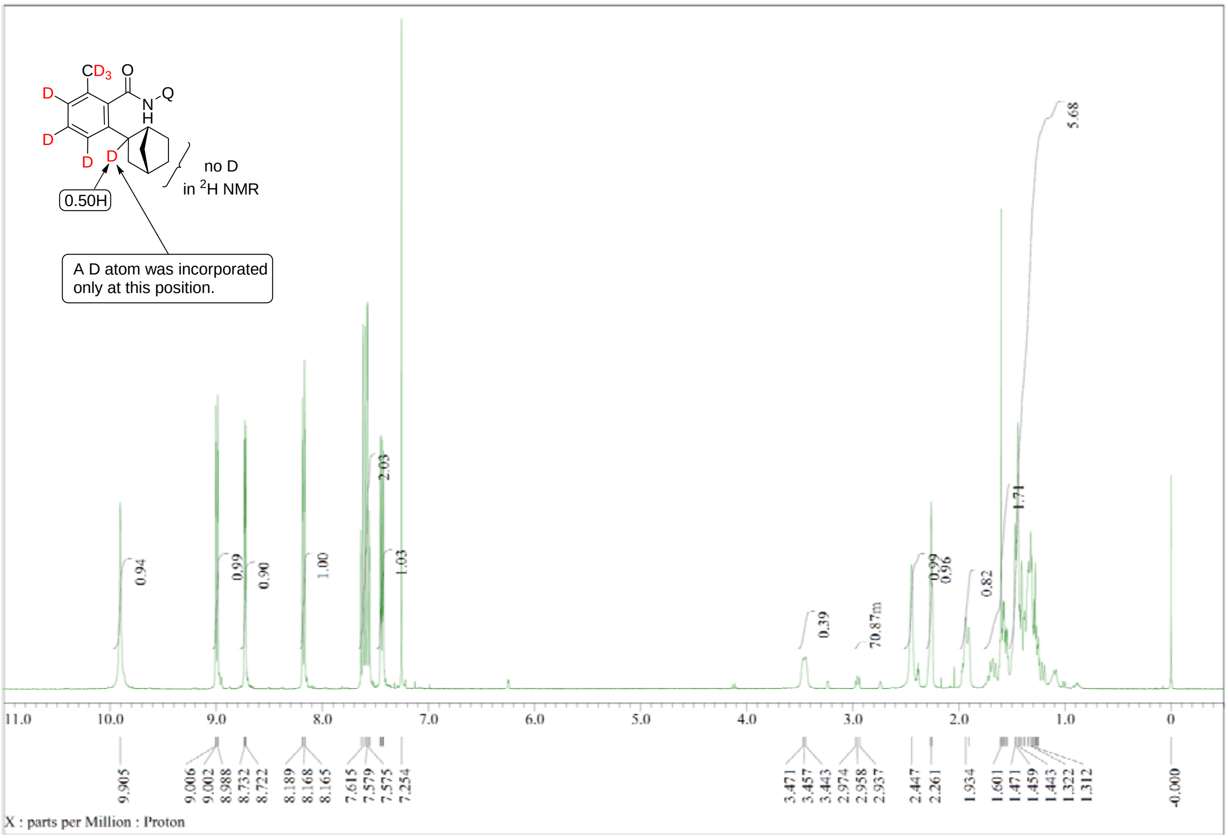

Supplementary Figure 2: ^{1}H NMR spectra for product of supplementary figure 1.

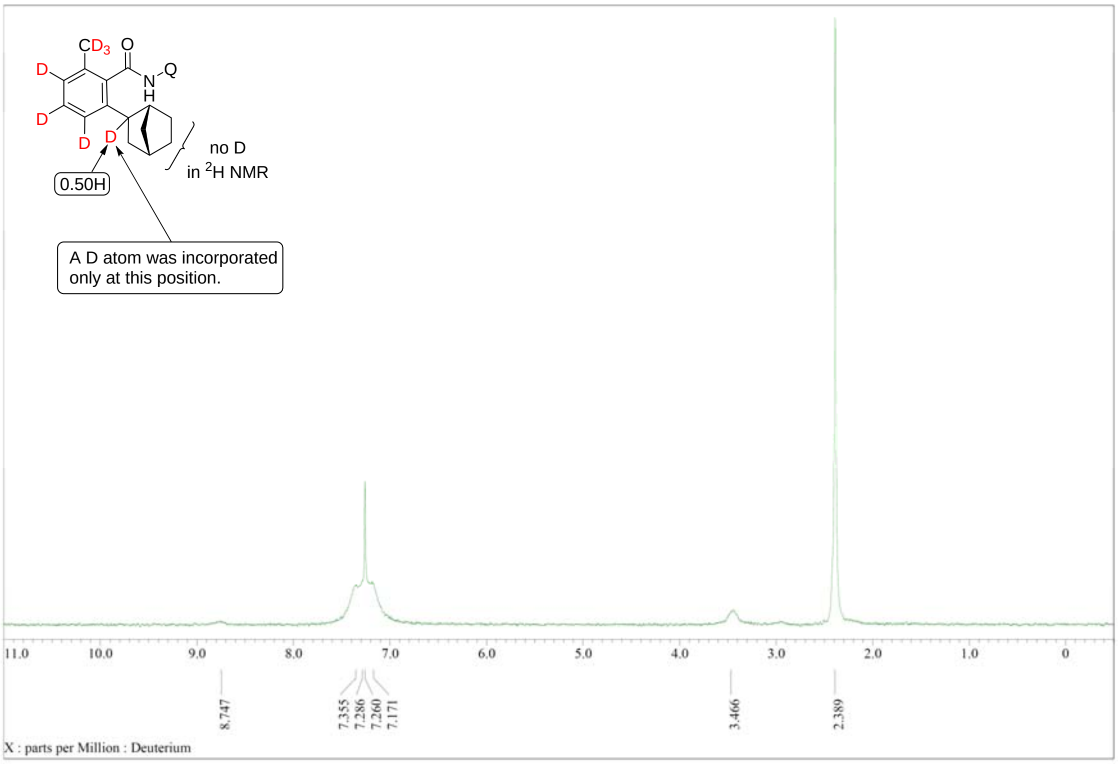

Supplementary Figure 3: ^{2}H NMR spectra for product of supplementary figure 1.

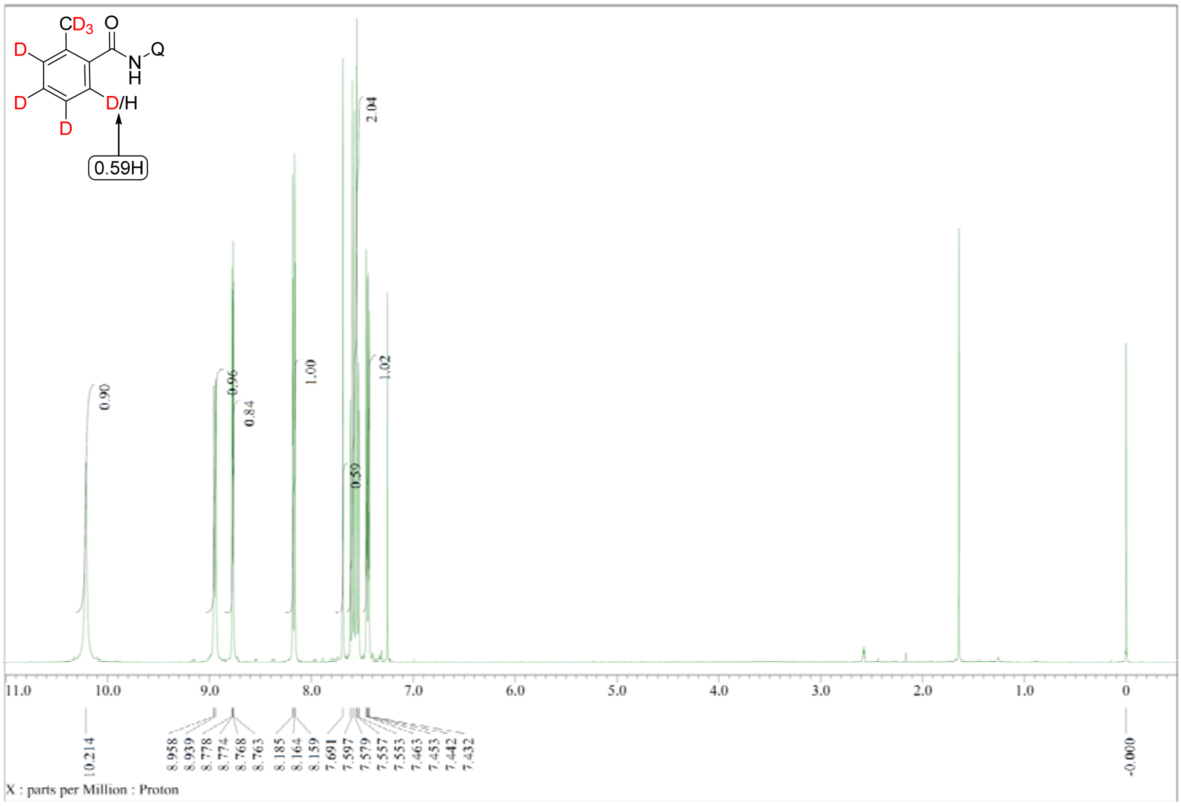

Supplementary Figure 4: ^{1}H NMR spectra for starting material of supplementary figure 1.

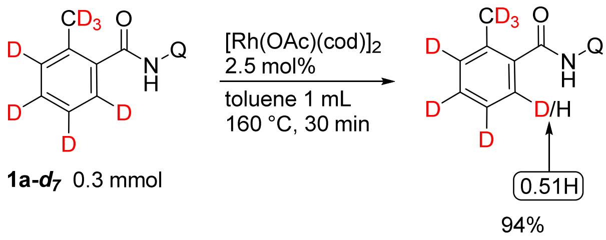

Supplementary Figure 5: Estimation of deuterium and hydrogen incorporation. (b)

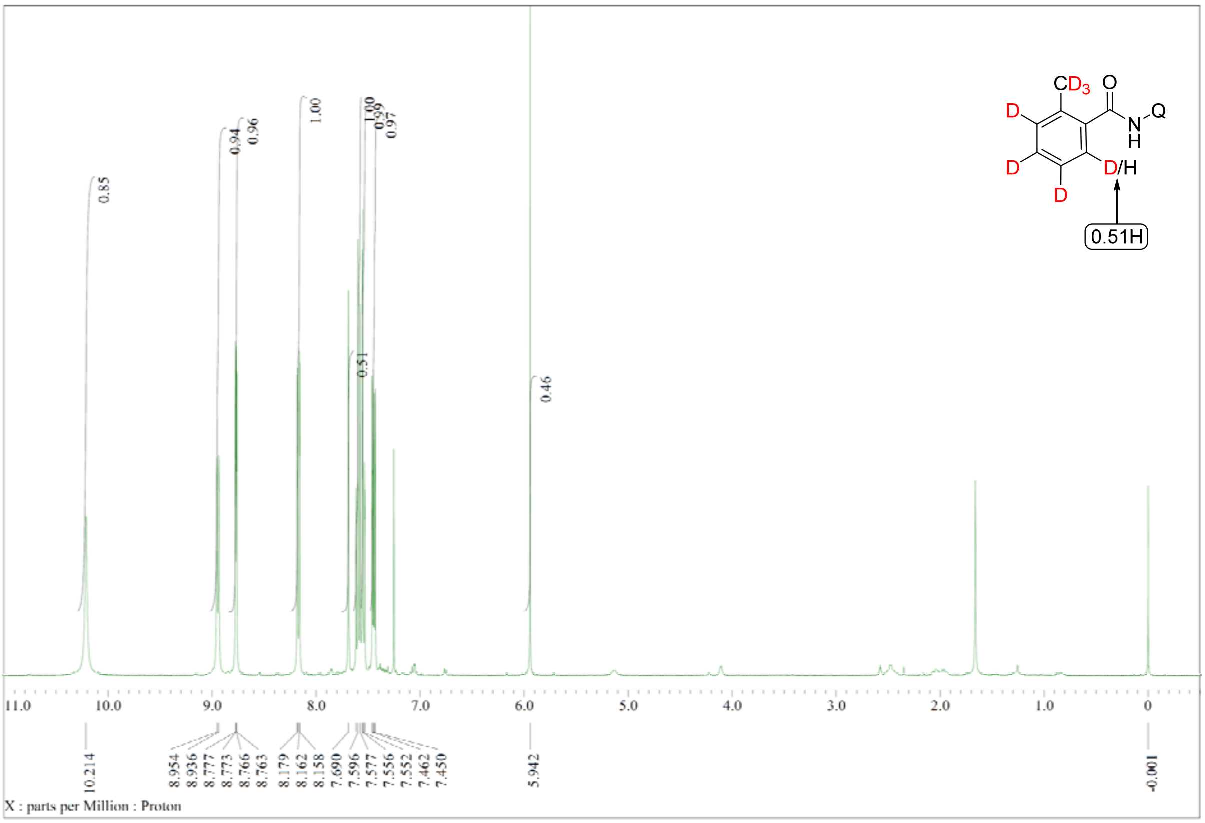

Supplementary Figure 6: ^{1}H NMR spectra for starting material of supplementary figure 5.

c.

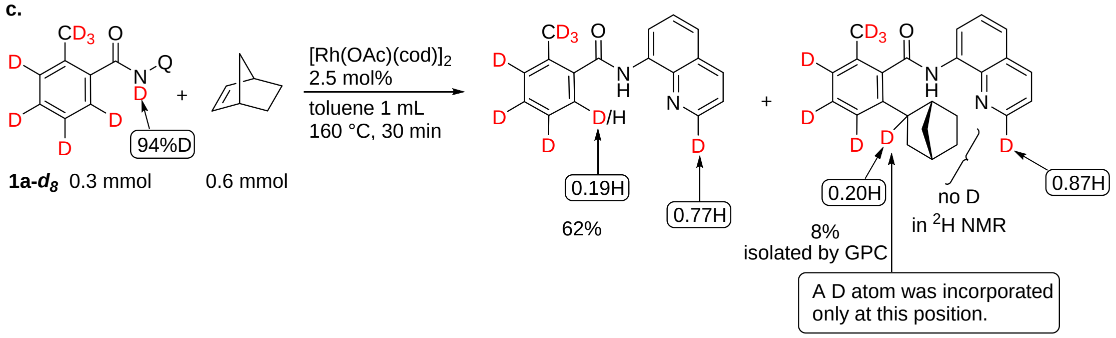

Supplementary Figure 7: Estimation of deuterium and hydrogen incorporation. (c)

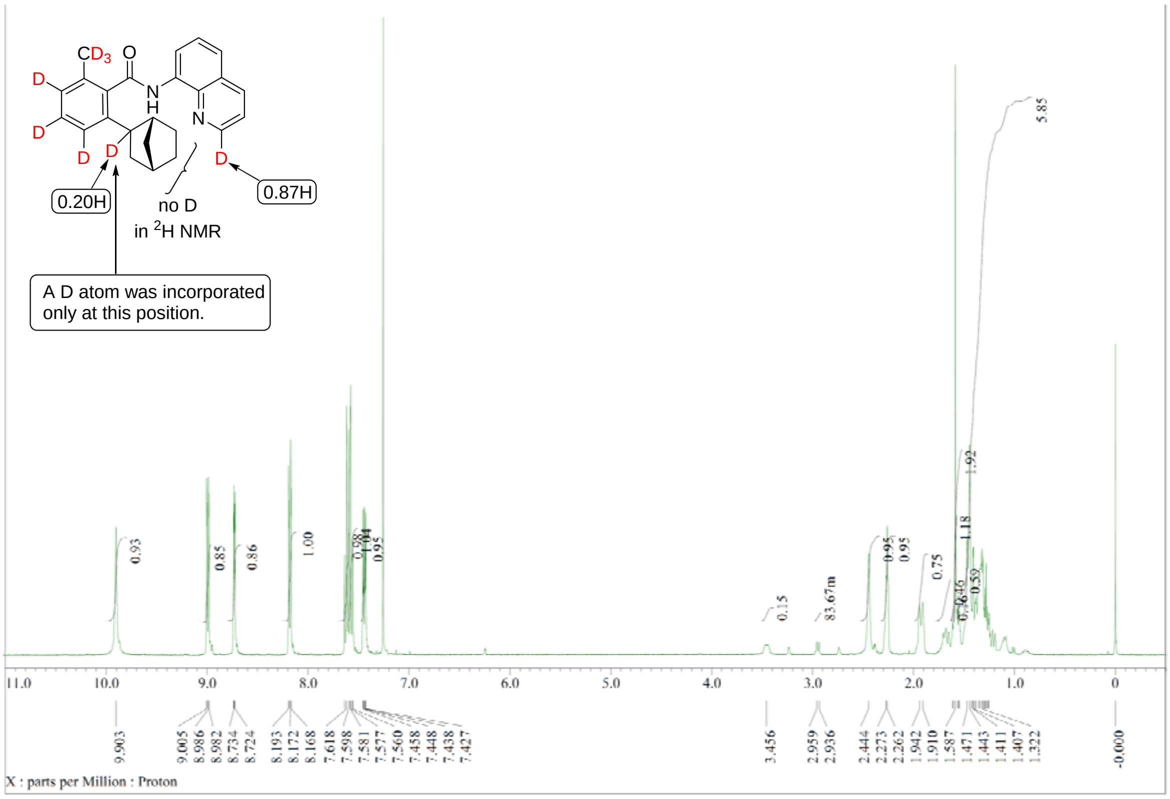

Supplementary Figure 8: ^{1}H NMR spectra for product of supplementary figure 7.

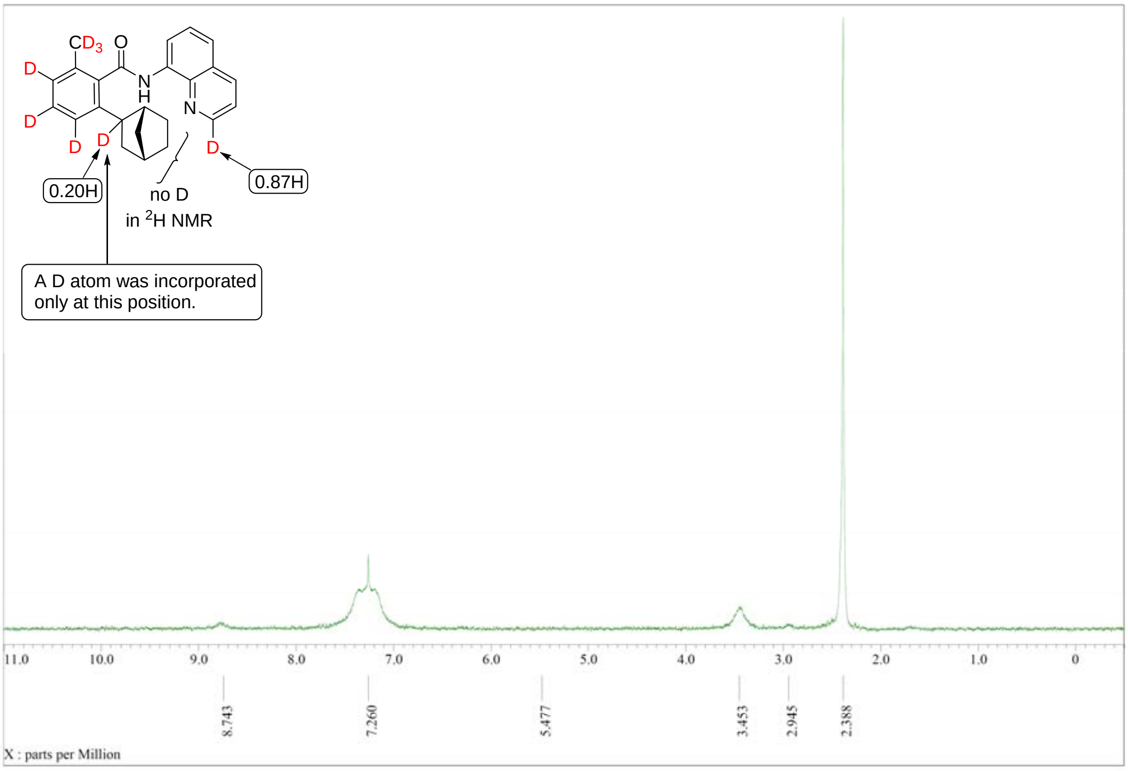

Supplementary Figure 9: ^{2}H NMR spectra for product of supplementary figure 7.

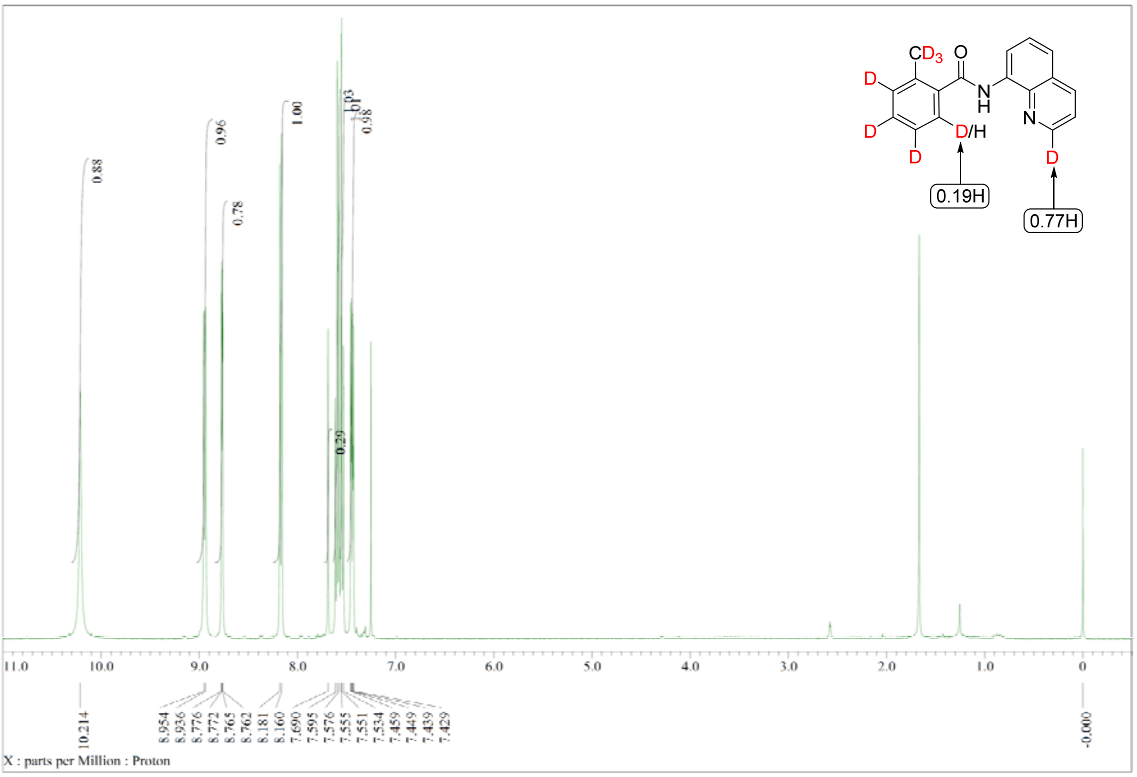

Supplementary Figure 10: ^{1}H NMR spectra for starting material of supplementary figure 7.

d.

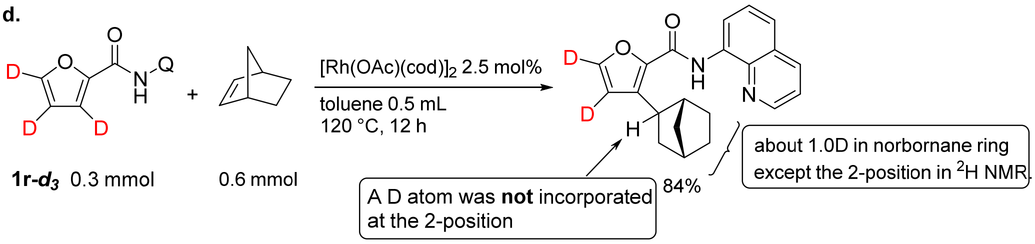

Supplementary Figure 11: Estimation of deuterium and hydrogen incorporation. (d)

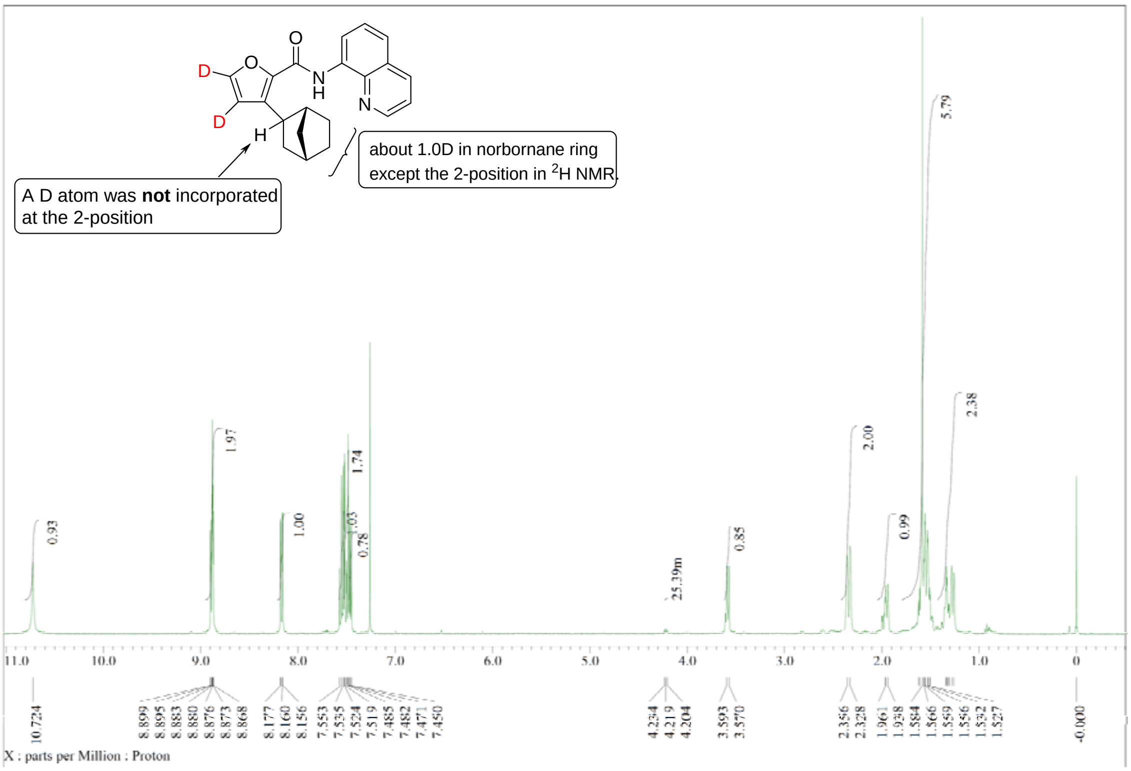

Supplementary Figure 12: ^{1}H NMR spectra for product of supplementary figure 11.

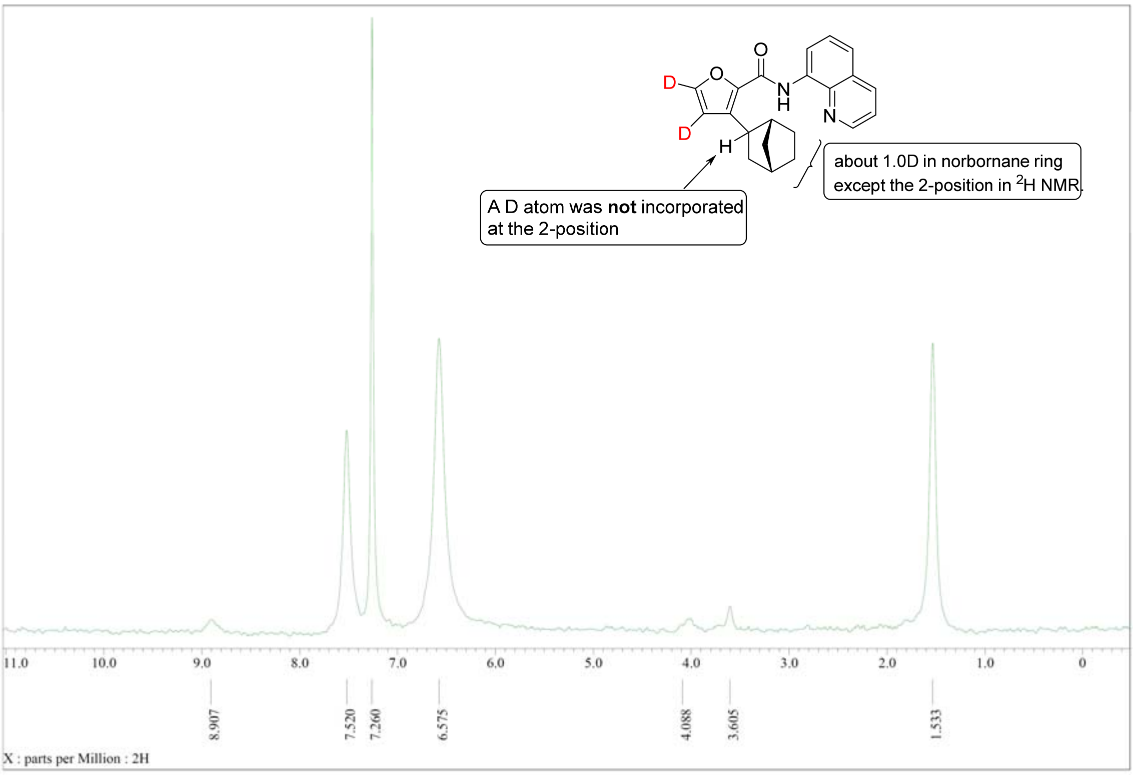

Supplementary Figure 13: ^{2}H NMR spectra for product of supplementary figure 11.

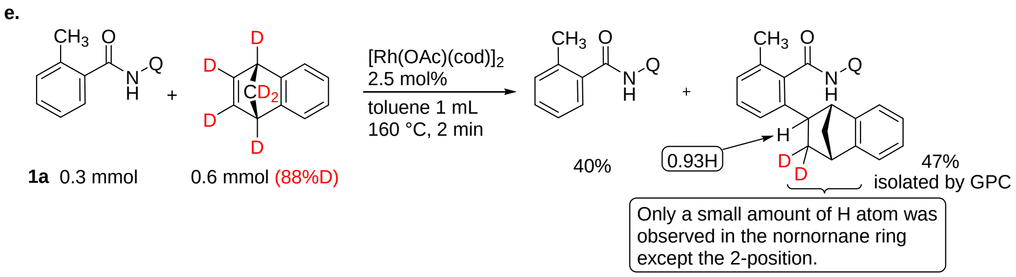

Supplementary Figure 14: Estimation of deuterium and hydrogen incorporation. (e)

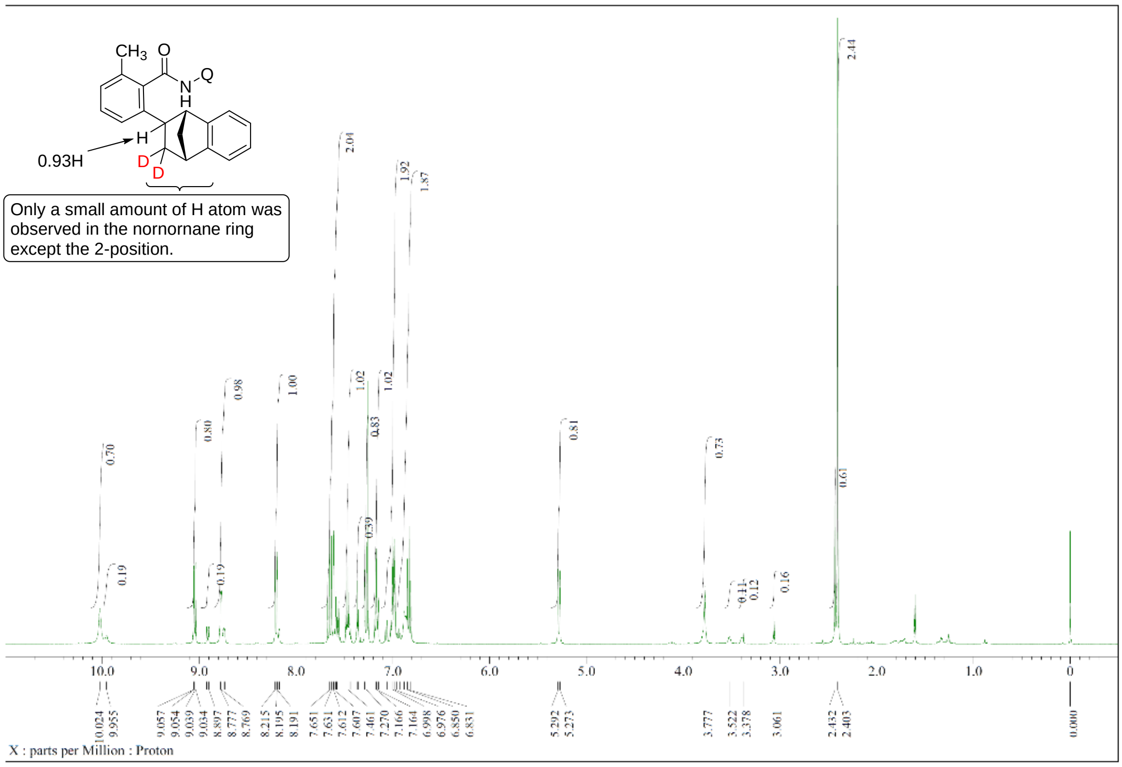

Supplementary Figure 15: ^{1}H NMR spectra for product of supplementary figure 14.

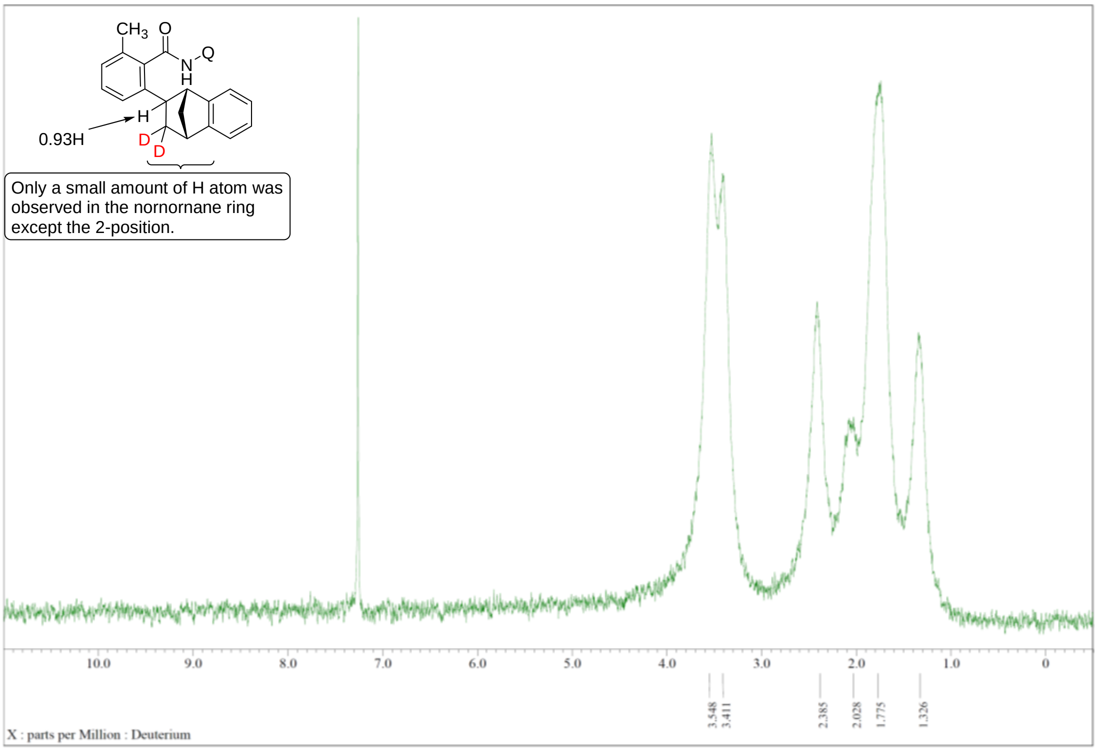

Supplementary Figure 16: ^{2}H NMR spectra for product of supplementary figure 14.

X-Ray Crystallographic Structure Analysis of 2a, 2r and 10a.

CCDC-1509925 contains the supplementary crystallographic data. X-Ray crystallographic structure analysis of 2-(Bicyclo[2.2.1]heptan-2-yl)-6-methyl-N-(quinolin-8-yl)benzamide (**2a**) was performed on Rigaku R-AXIS RAPID imaging plate diffractometer with graphite monochromated Mo Kα radiation (λ = 0.71075 Å). The data was collected at 150 K±1 K using ω scan in the 2θ range of 130.0-190.0 deg. A total of 21993 reflection were measured, of which 5543 were independent reflections (R_{int} = 0.0986). The structure was solved by direct methods (SIR92) and refined by the full matrix least-squares on F^2 (Crystals). All non-hydrogen atoms were refined anisotropically and all hydrogen atoms were placed using riding model. The crystal data are as follows: (**2a**_CCDC 1509925): $C_{24}H_{24}N_2O_1$, FW = 356.47, crystal size 0.30 × 0.15 × 0.05 mm^3, monoclinic, space group P21/c, a = 9.7758 (5)Å, b = 7.8985 (4)Å, c = 23.7398 (10)Å, β = 92.7633 (15)°, V = 1830.91 (15) Å^3, Z = 4, Dc = 1.293 g/cm^3. The refinement converged to $R1$ = 0.1119 for I > 2σ (I), $wR2$ = 0.3806 for all data, GOF = 1.101 for all data.

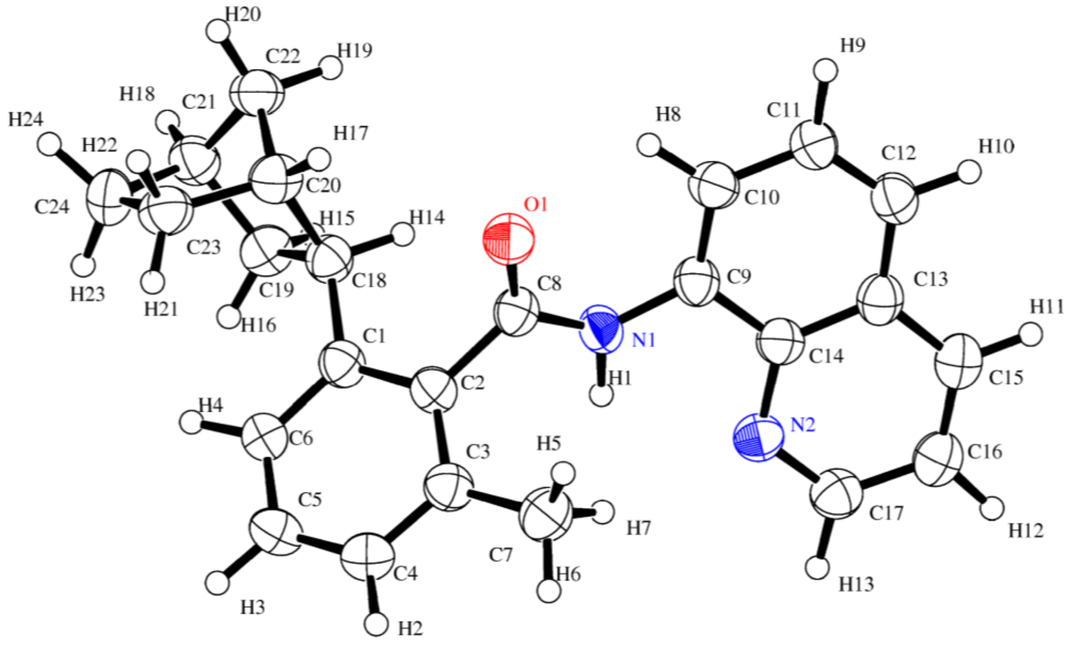

Supplementary Figure 17: ORTEP of 2a.

CCDC-1509926 contains the supplementary crystallographic data. X-Ray crystallographic structure analysis of (Bicyclo[2.2.1]heptan-2-yl)-N-(quinolin-8-yl)furan-2-carboxamide (**2r**) was performed on Rigaku R-AXIS RAPID imaging plate diffractometer with graphite monochromated Mo Kα radiation (λ = 0.71075 Å). The data was collected at 150 K±1 K using ω scan in the 2θ range of 130.0-190.0 deg. A total of 24240 reflection were measured, of which 10365 were independent reflections (R_{int} = 0.1129). The structure was solved by direct methods (SIR92) and refined by the full matrix least-squares on F^2 (Crystals). All non-hydrogen atoms were refined anisotropically and all hydrogen atoms were placed using riding model. The crystal data are as follows: (**2r**_CCDC 1509925): $C_{21}H_{20}N_2O_2$, FW = 332.40, crystal size 0.50 × 0.15 × 0.10 mm^3, monoclinic, space group Cc, a = 26.249 (3)Å, b = 16.6557 (16) Å, c = 12.9819 (13)Å, β = 117.836 (3)°, V = 5018.9 (3)Å^3, Z = 12, Dc = 1.320 g/cm^3. The refinement converged to $R1$ = 0.1250 for $I > 2\sigma(I)$, $wR2$ = 0.3949 for all data, GOF = 1.122 for all data.

An X-Ray crystallographic structure analysis that **2r** is composed of three molecules with slightly different bond lengths and angles in each unit. However, only one structure is shown below because all have the same configuration.

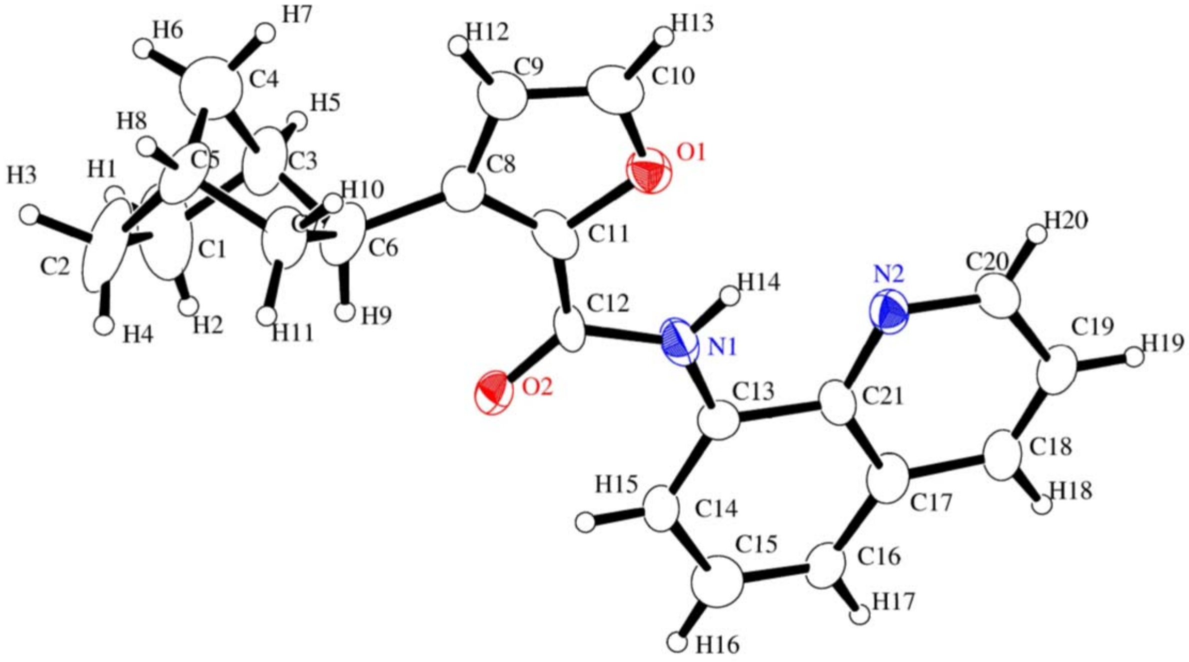

Supplementary Figure 18: ORTEP of 2r.

CCDC-1535668 contains the supplementary crystallographic data. X-Ray crystallographic structure analysis of Di-tert-butyl 5-(3-methyl-2-(quinolin-8-ylcarbamoyl)phenyl)-2,3-diazabicyclo[2.2.1]heptane-2,3-dicarboxylate (**10a**) was performed on Rigaku R-AXIS RAPID imaging plate diffractometer with graphite monochromated Cu Kα radiation (λ = 1.54187 Å). The data was collected at 296 K$\pm$1 K using ω scan in the 2θ range of 80.0-260.0 deg. A total of 49354 reflection were measured, of which 5308 were independent reflections (R_{int} = 0.0509). The structure was solved by direct methods (SIR92) and refined by the full matrix least-squares on F^2 (Crystals). All non-hydrogen atoms were refined anisotropically and all hydrogen atoms were placed using riding model. The crystal data are as follows: (**10a**_CCDC 1535668): $C_{32}H_{38}N_5O_4$, FW = 556.68, crystal size 0.40 × 0.40 × 0.20 mm^3, monoclinic, space group P21/c, a = 13.5238(3)Å, b = 12.1666(2)Å, c = 17.8630(3)Å, β = 97.2914(7)°, V = 2915.39(9) Å^3, Z = 4, Dc = 1.268 g/cm^3. The refinement converged to $R1$ = 0.0408 for $I > 2\sigma(I)$, $wR2$ = 0.1003 for all data, GOF = 1.051 for all data.

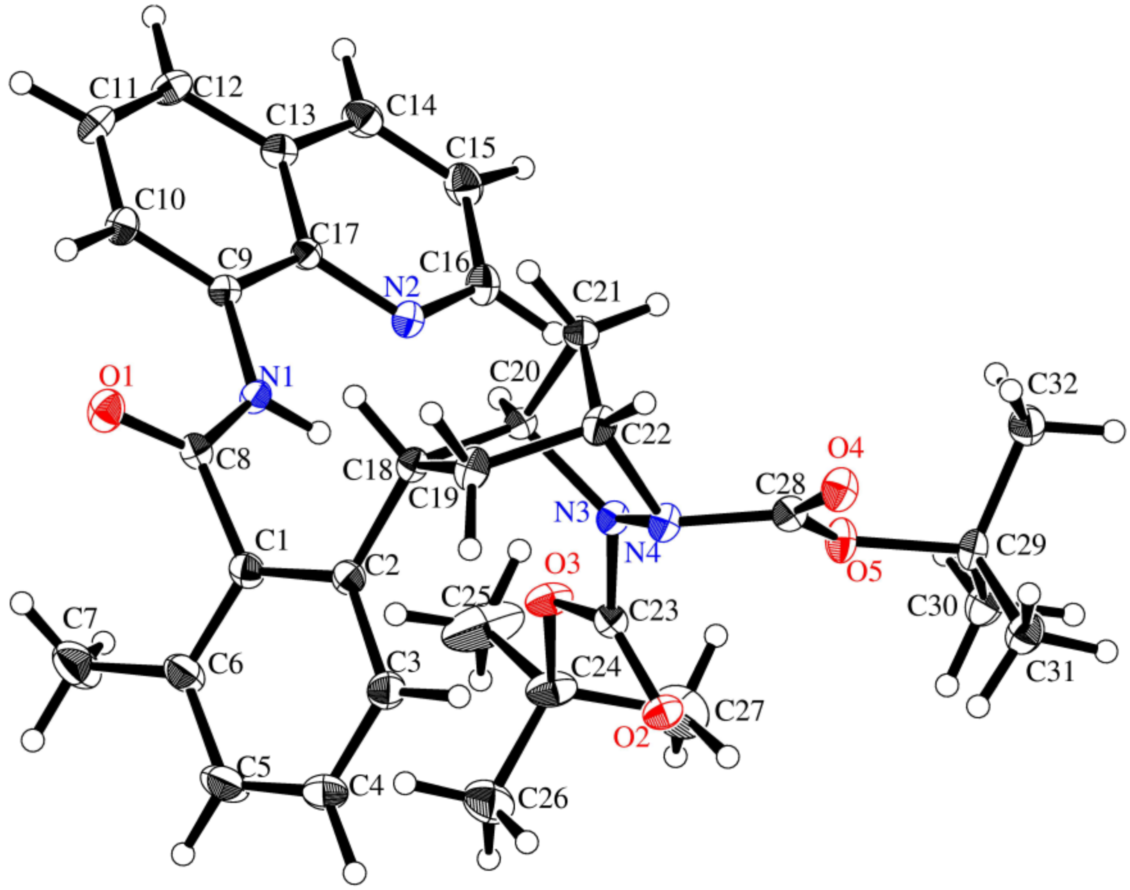

Supplementary Figure 19: ORTEP of 10a.

Copies of ^{1}H and ^{13}C NMR Spectra

Supplementary Figure 20: 1H NMR, 13C NMR spectra for 2a.

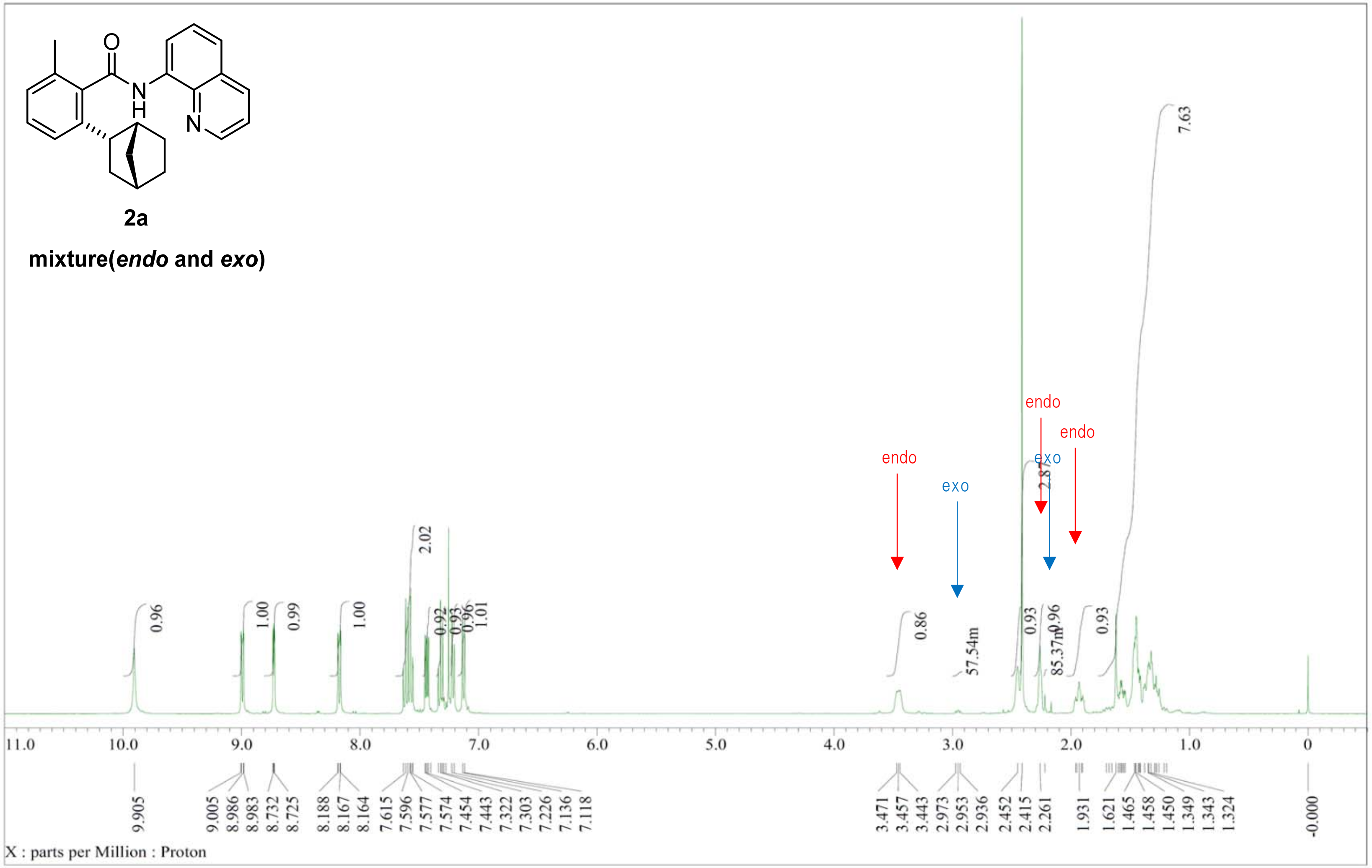

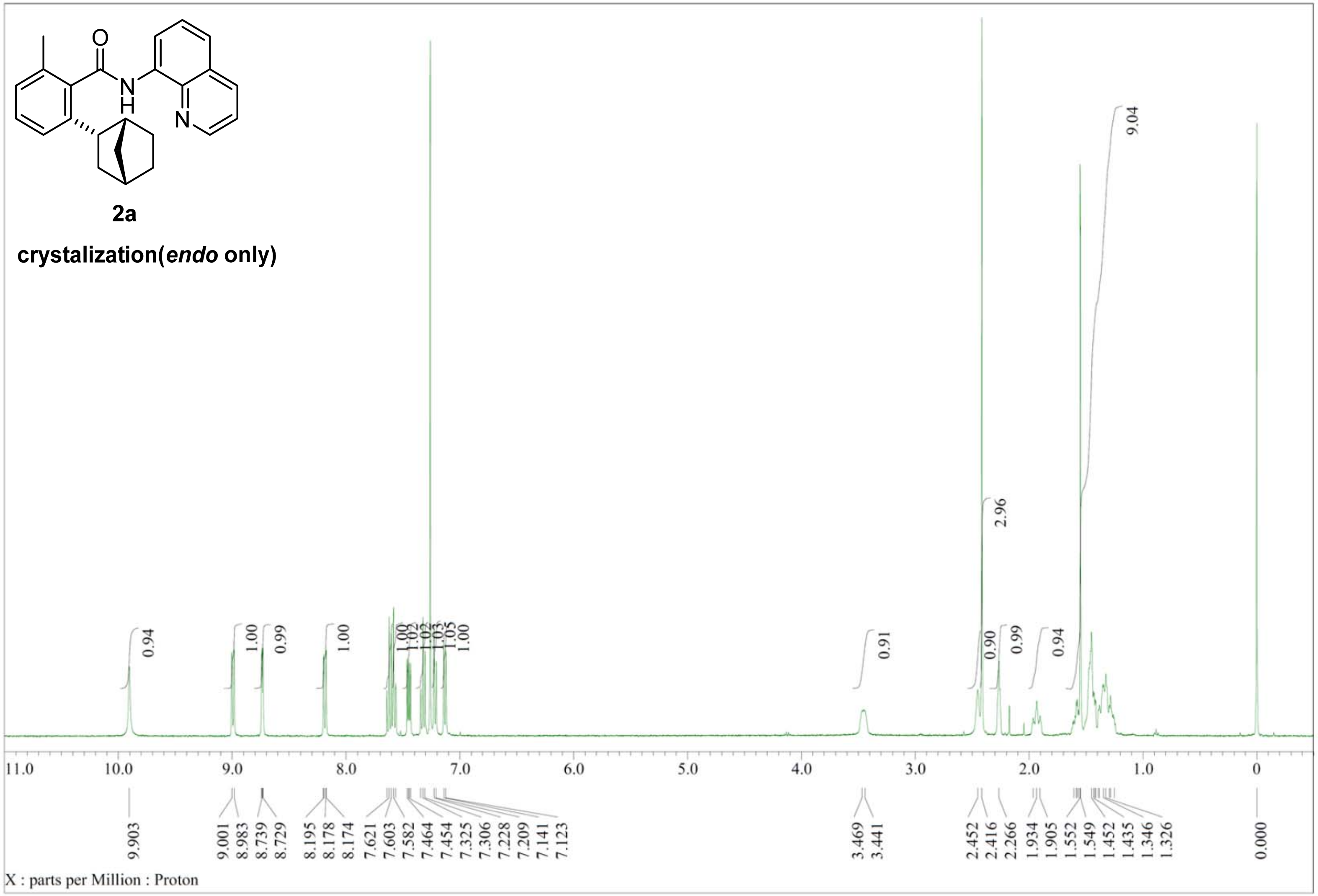
O
N
H
N
2a
crystalization(endo only)
0.94
1.00
0.99
1.00
1.00
1.02
1.02
1.03
1.05
1.00
0.91
2.96
0.90
0.99
0.94
9.04
11.0
10.0
9.0
8.0
7.0
6.0
5.0
4.0
3.0
2.0
1.0
0
9.903
9.001
8.983
8.739
8.729
8.195
8.178
8.174
7.621
7.603
7.582
7.464
7.454
7.325
7.306
7.228
7.209
7.141
7.123
3.469
3.441
2.452
2.416
2.266
1.934
1.905
1.552
1.549
1.452
1.435
1.346
1.326
0.000
X : parts per Million : Proton

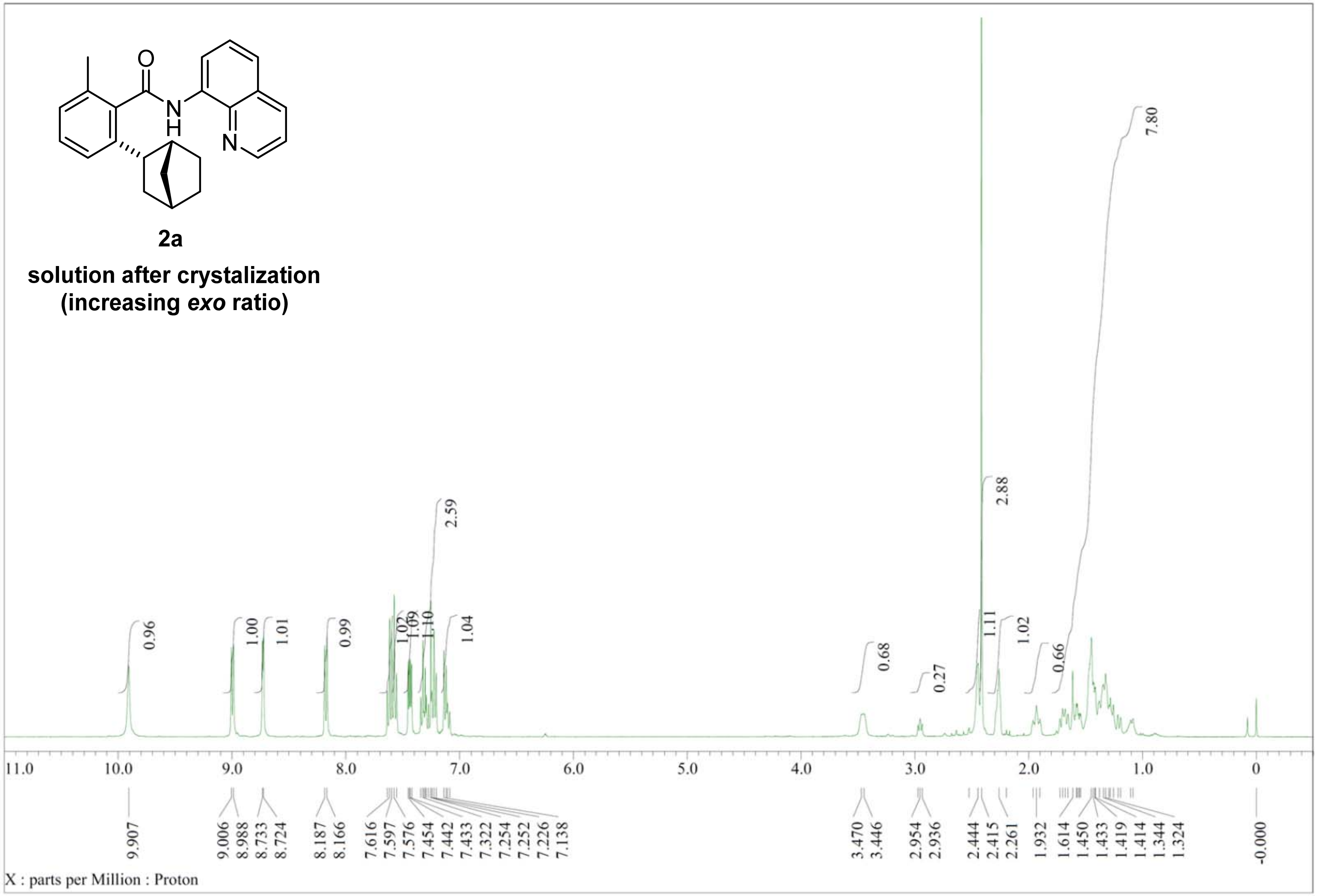

2a
solution after crystalization
(increasing exo ratio)
X : parts per Million : Proton

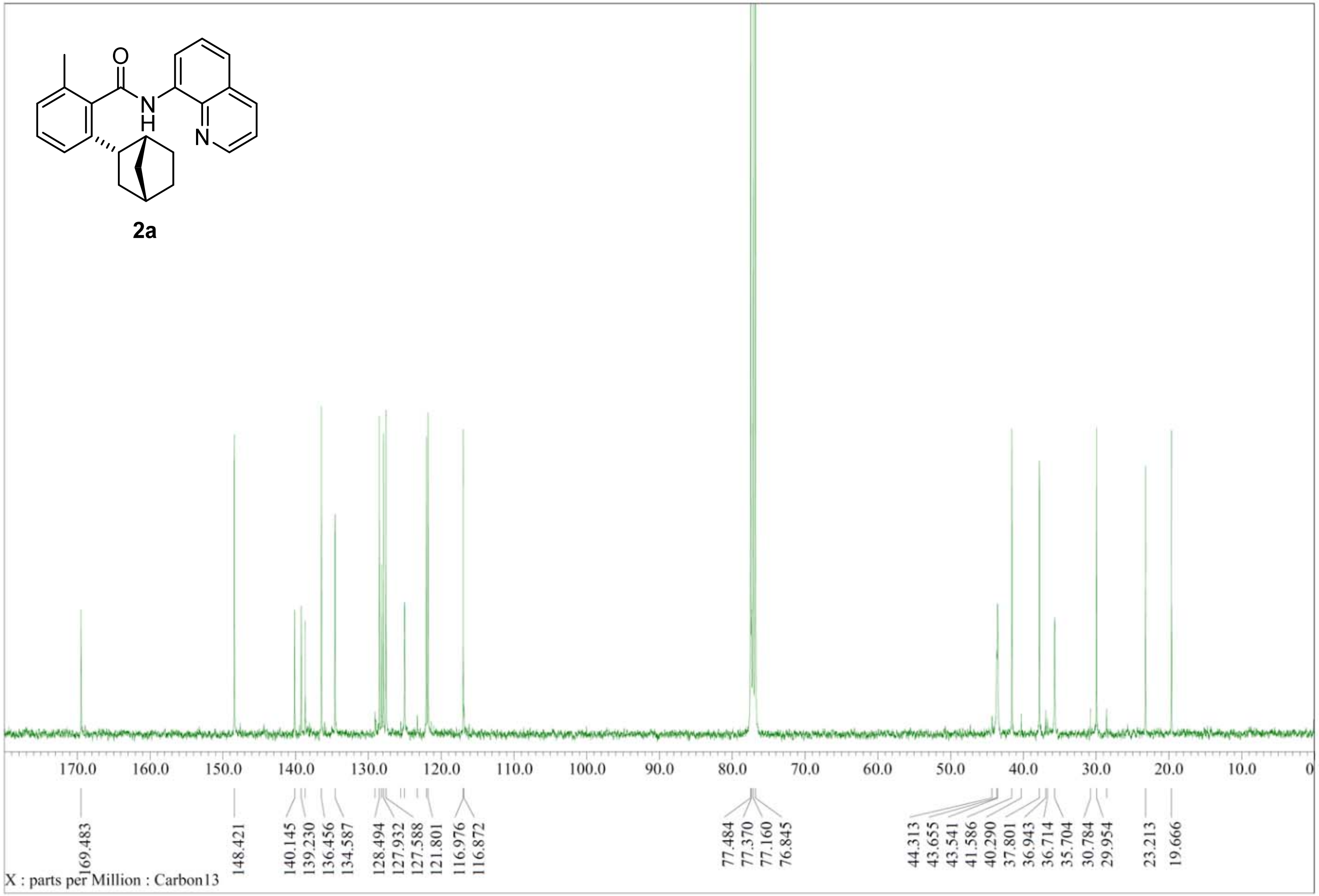
O
N
H
N
2a
170.0
160.0
150.0
140.0
130.0
120.0
110.0
100.0
90.0
80.0
70.0
60.0
50.0
40.0
30.0
20.0
10.0
0
169.483
148.421
140.145
139.230
136.456
134.587
128.494
127.932
127.588
121.801
116.976
116.872
77.484
77.370
77.160
76.845
44.313
43.655
43.541
41.586
40.290
37.801
36.943
36.714
35.704
30.784
29.954
23.213
19.666
X : parts per Million : Carbon13

Supplementary Figure 21: ^{1}H NMR, ^{13}C NMR spectra for 2b.

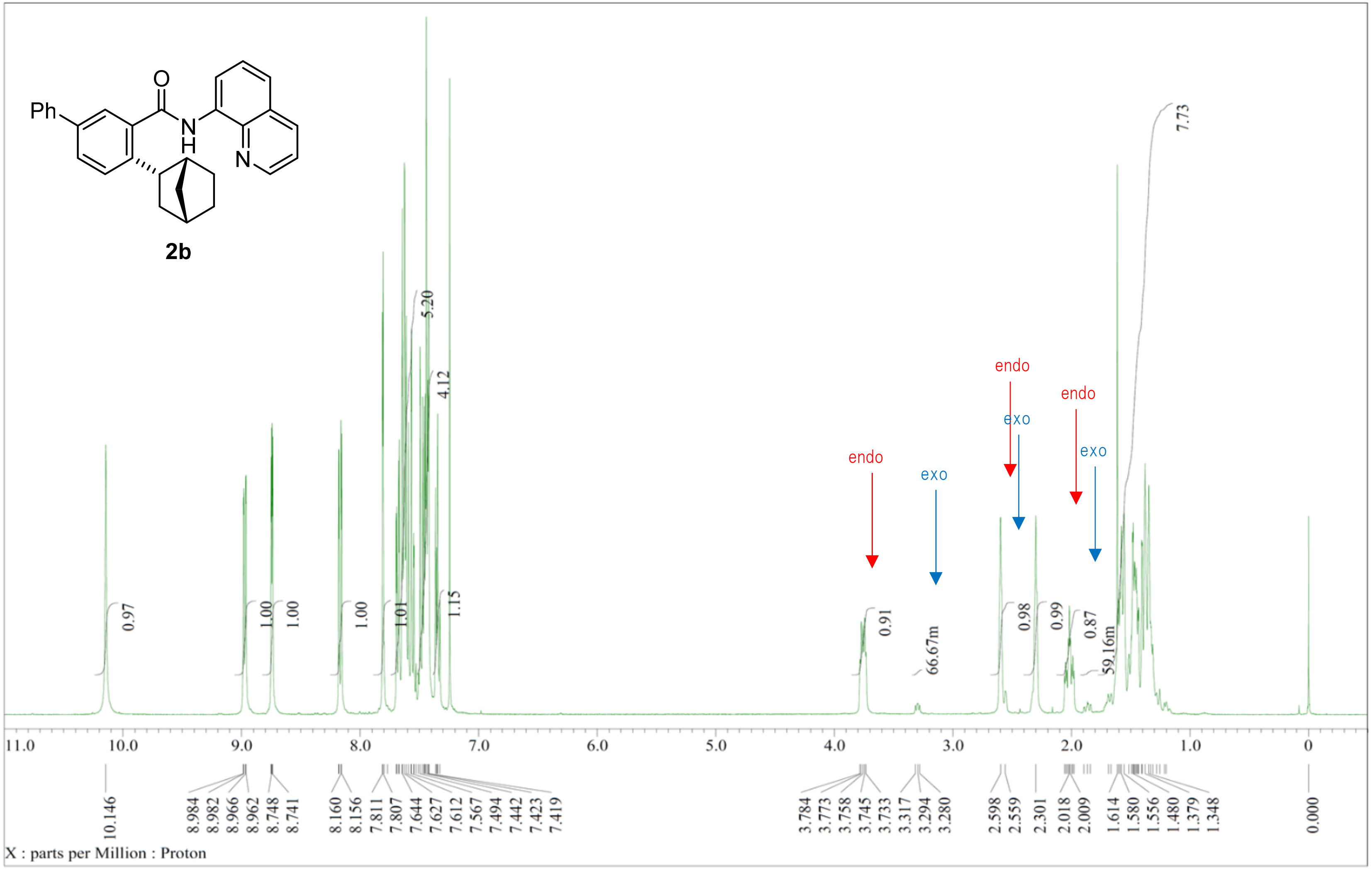

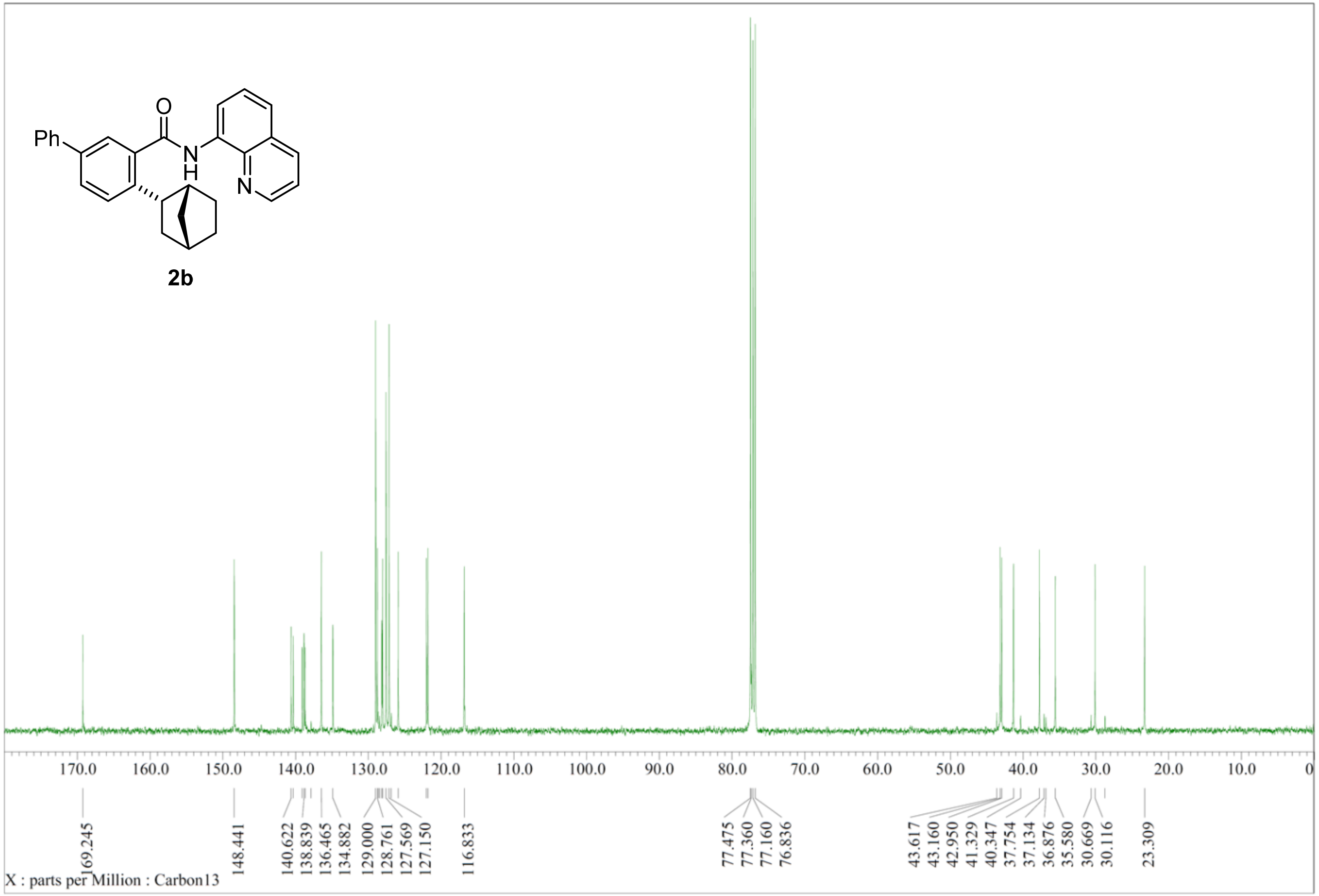
Ph
O
N
H
N
2b
170.0
160.0
150.0
140.0
130.0
120.0
110.0
100.0
90.0
80.0
70.0
60.0
50.0
40.0
30.0
20.0
10.0
0
169.245
148.441
140.622
138.839
136.465
134.882
129.000
128.761
127.569
127.150
116.833
77.475
77.360
77.160
76.836
43.617
43.160
42.950
41.329
40.347
37.754
37.134
36.876
35.580
30.669
30.116
23.309
X : parts per Million : Carbon13

Supplementary Figure 22: ^{1}H NMR, ^{13}C NMR spectra for 2c.

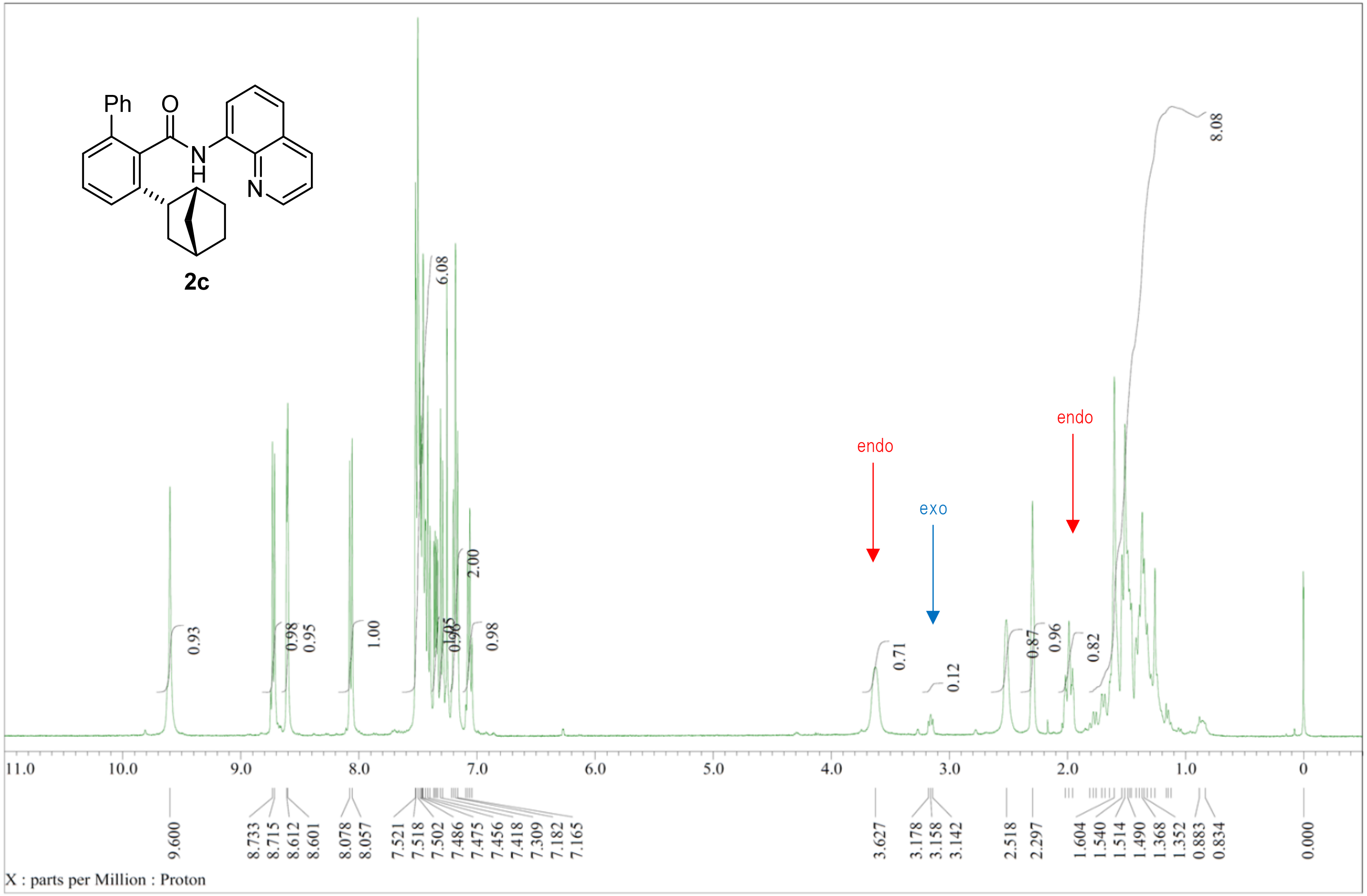

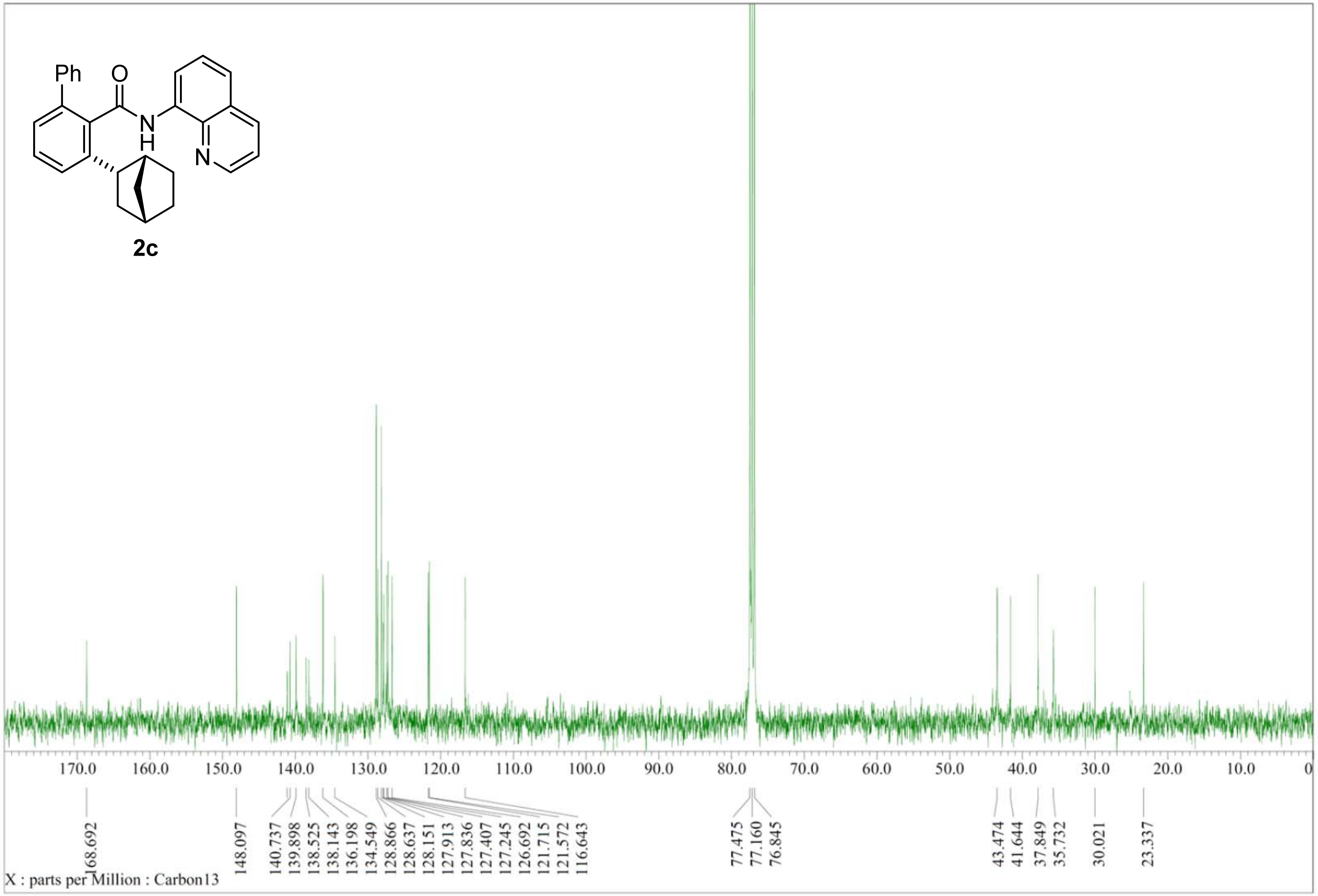
Ph
O
N
H
N
2c
170.0
160.0
150.0
140.0
130.0
120.0
110.0
100.0
90.0
80.0
70.0
60.0
50.0
40.0
30.0
20.0
10.0
0
168.692
148.097
140.737
139.898
138.525
138.143
136.198
134.549
128.866
128.637
128.151
127.913
127.836
127.407
127.245
126.692
121.715
121.572
116.643
77.475
77.160
76.845
43.474
41.644
37.849
35.732
30.021
23.337
X : parts per Million : Carbon13

Supplementary Figure 23: ^{1}H NMR, ^{13}C NMR spectra for 2d.

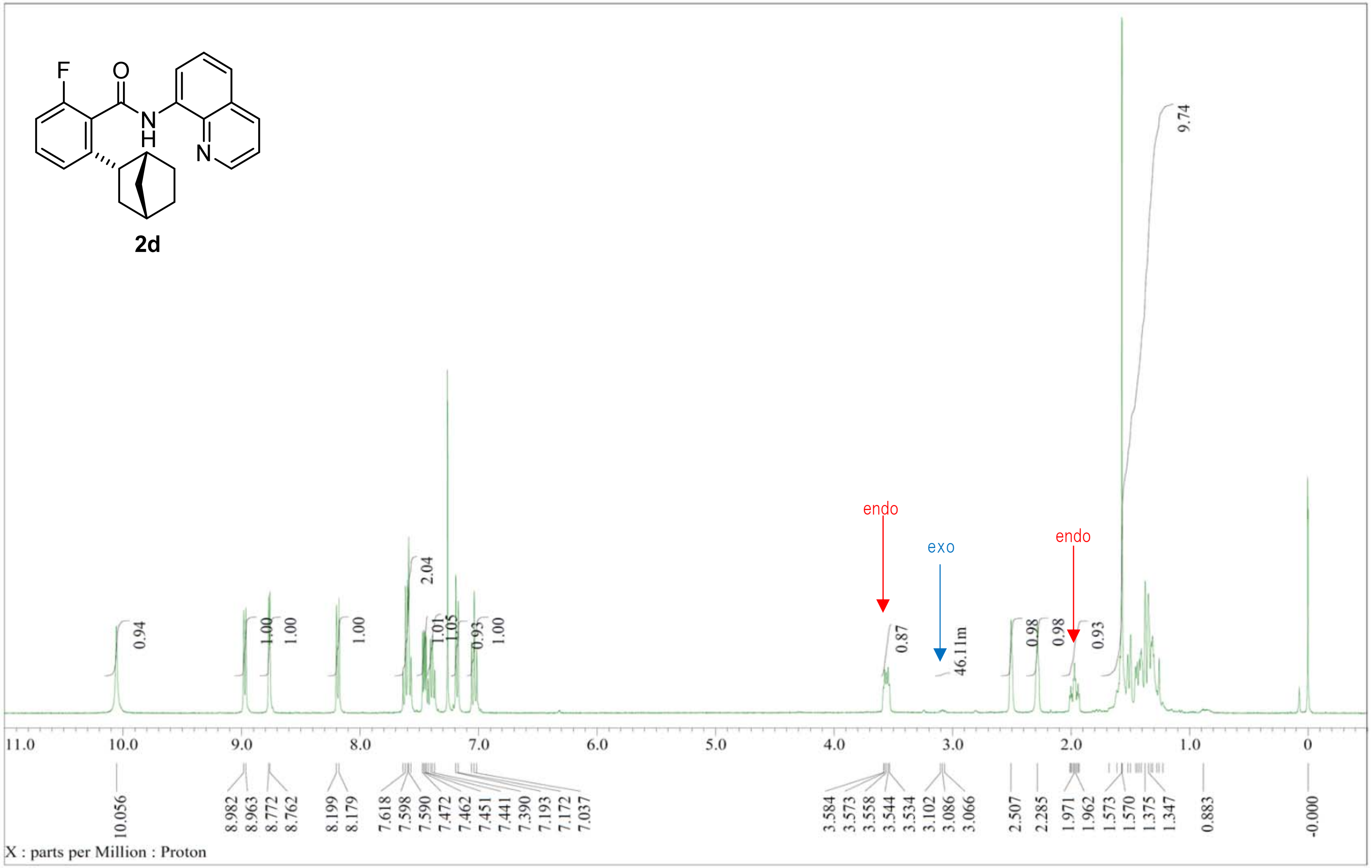

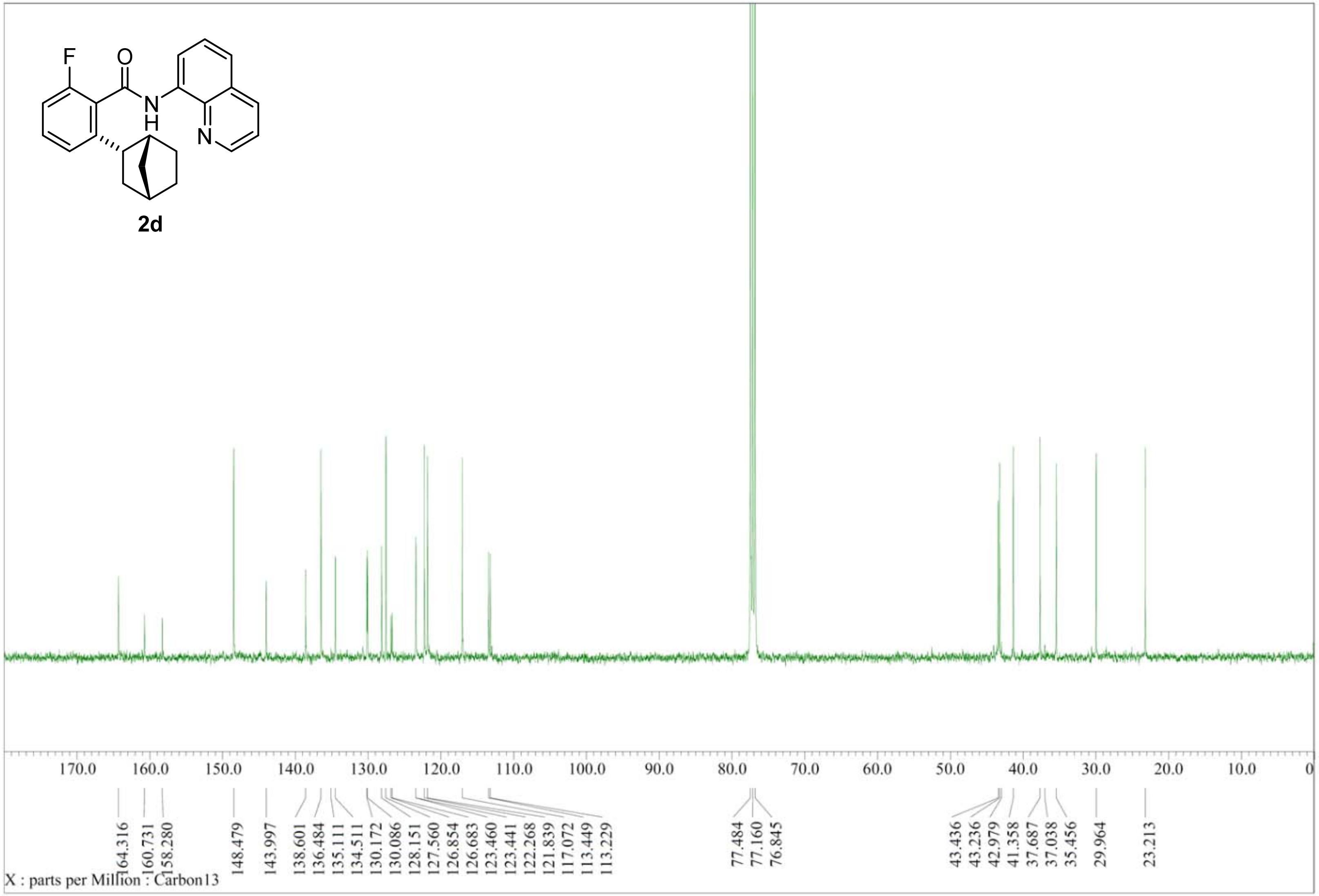
F
O
N
H
N
2d
170.0
160.0
150.0
140.0
130.0
120.0
110.0
100.0
90.0
80.0
70.0
60.0
50.0
40.0
30.0
20.0
10.0
0
164.316
160.731
158.280
148.479
143.997
138.601
136.484
135.111
134.511
130.172
130.086
128.151
127.560
126.854
126.683
123.460
123.441
122.268
121.839
117.072
113.449
113.229
77.484
77.160
76.845
43.436
43.236
42.979
41.358
37.687
37.038
35.456
29.964
23.213
X : parts per Million : Carbon13

Supplementary Figure 24: ^{1}H NMR, ^{13}C NMR spectra for 2e.

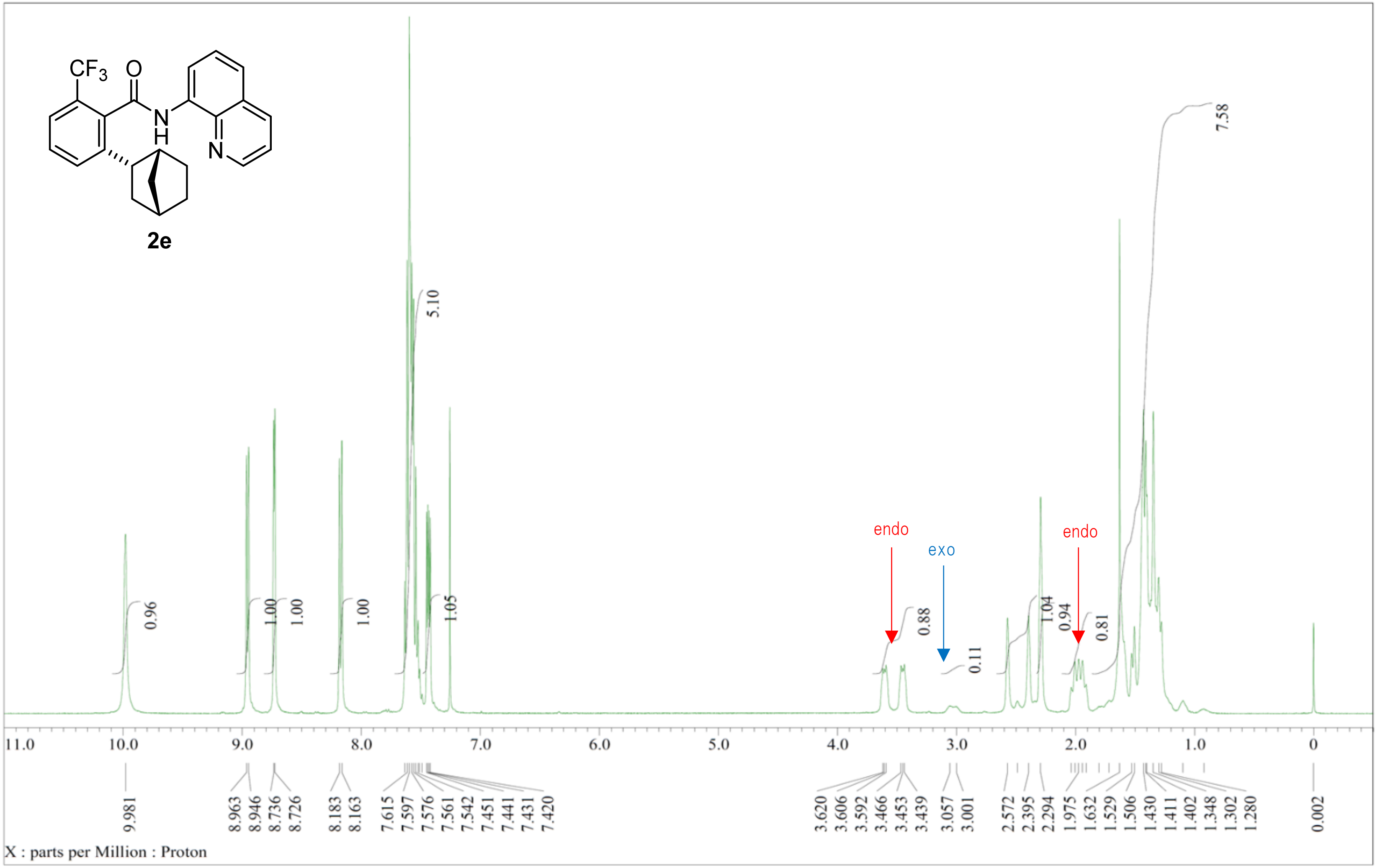

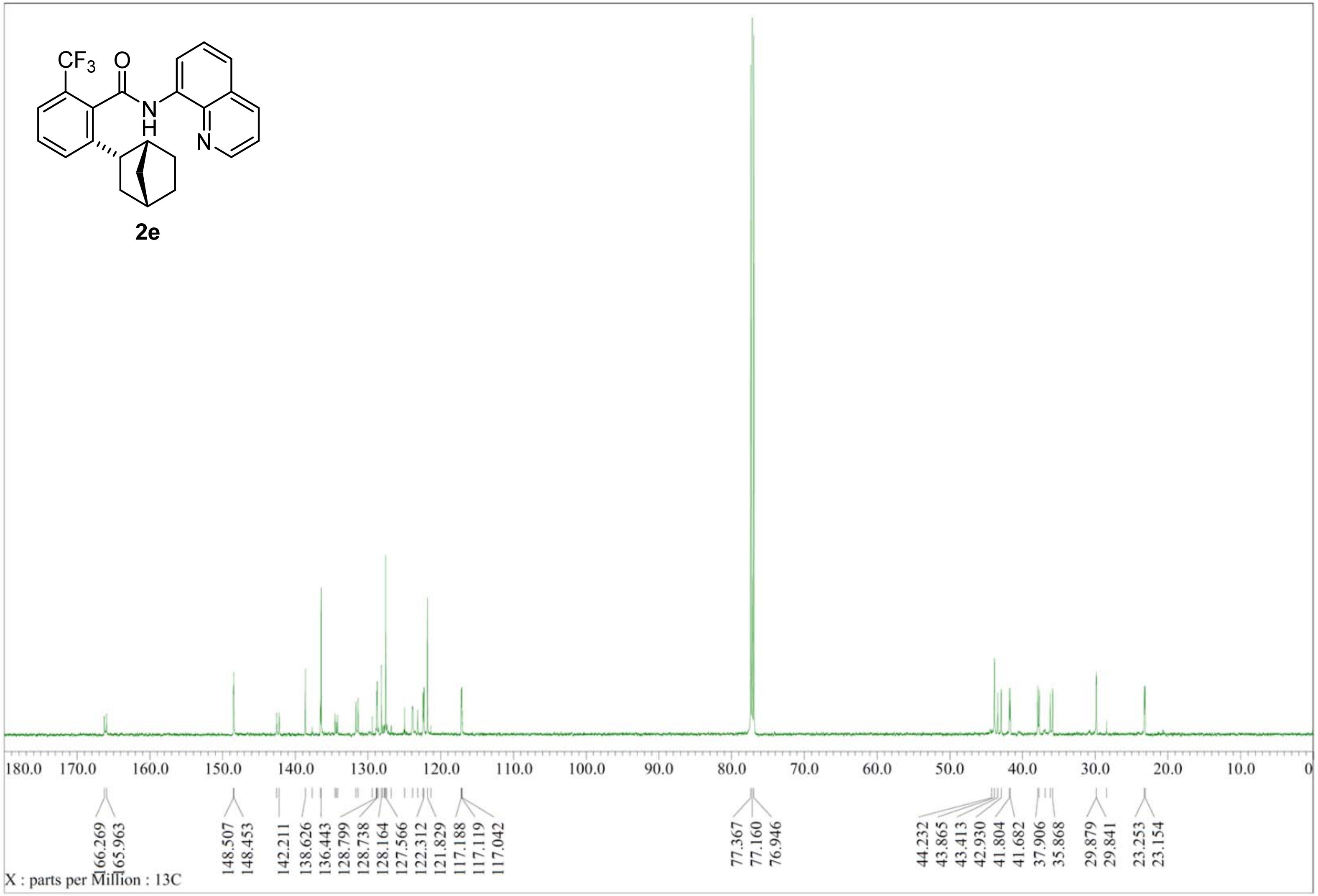
2e
180.0 170.0 160.0 150.0 140.0 130.0 120.0 110.0 100.0 90.0 80.0 70.0 60.0 50.0 40.0 30.0 20.0 10.0 0
166.269
165.963
148.507
148.453
142.211
138.626
136.443
128.799
128.738
128.164
127.566
122.312
121.829
117.188
117.119
117.042
77.367
77.160
76.946
44.232
43.865
43.413
42.930
41.804
41.682
37.906
35.868
29.879
29.841
23.253
23.154
X : parts per Million : 13C

Supplementary Figure 25: ^{1}H NMR, ^{13}C NMR spectra for 2f.

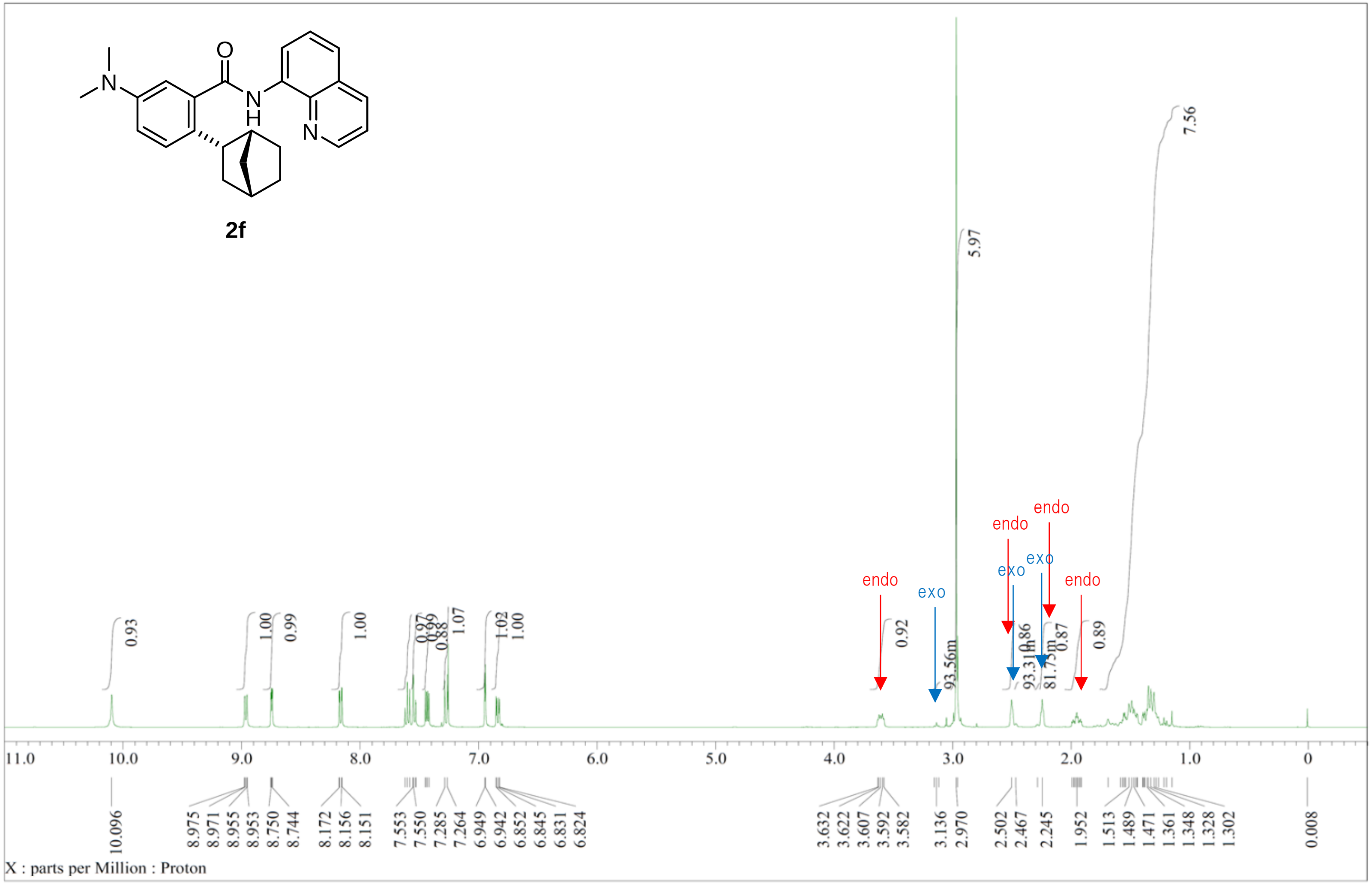

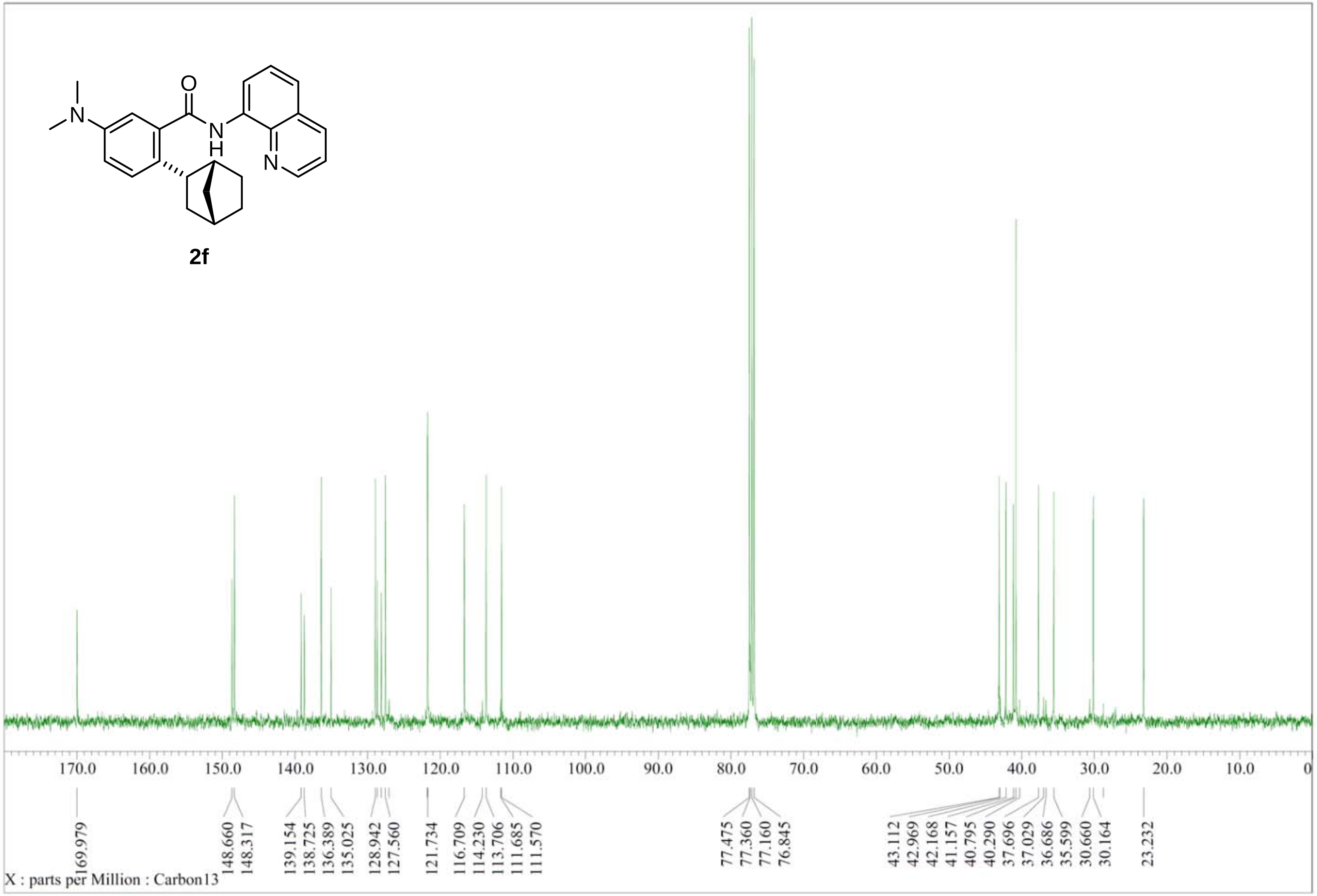

2f
170.0 160.0 150.0 140.0 130.0 120.0 110.0 100.0 90.0 80.0 70.0 60.0 50.0 40.0 30.0 20.0 10.0 0
169.979
148.660
148.317
139.154
138.725
136.389
135.025
128.942
127.560
121.734
116.709
114.230
113.706
111.685
111.570
77.475
77.360
77.160
76.845
43.112
42.969
42.168
41.157
40.795
40.290
37.696
37.029
36.686
35.599
30.660
30.164
23.232
X : parts per Million : Carbon13

Supplementary Figure 26: ^{1}H NMR, ^{13}C NMR spectra for 2g.

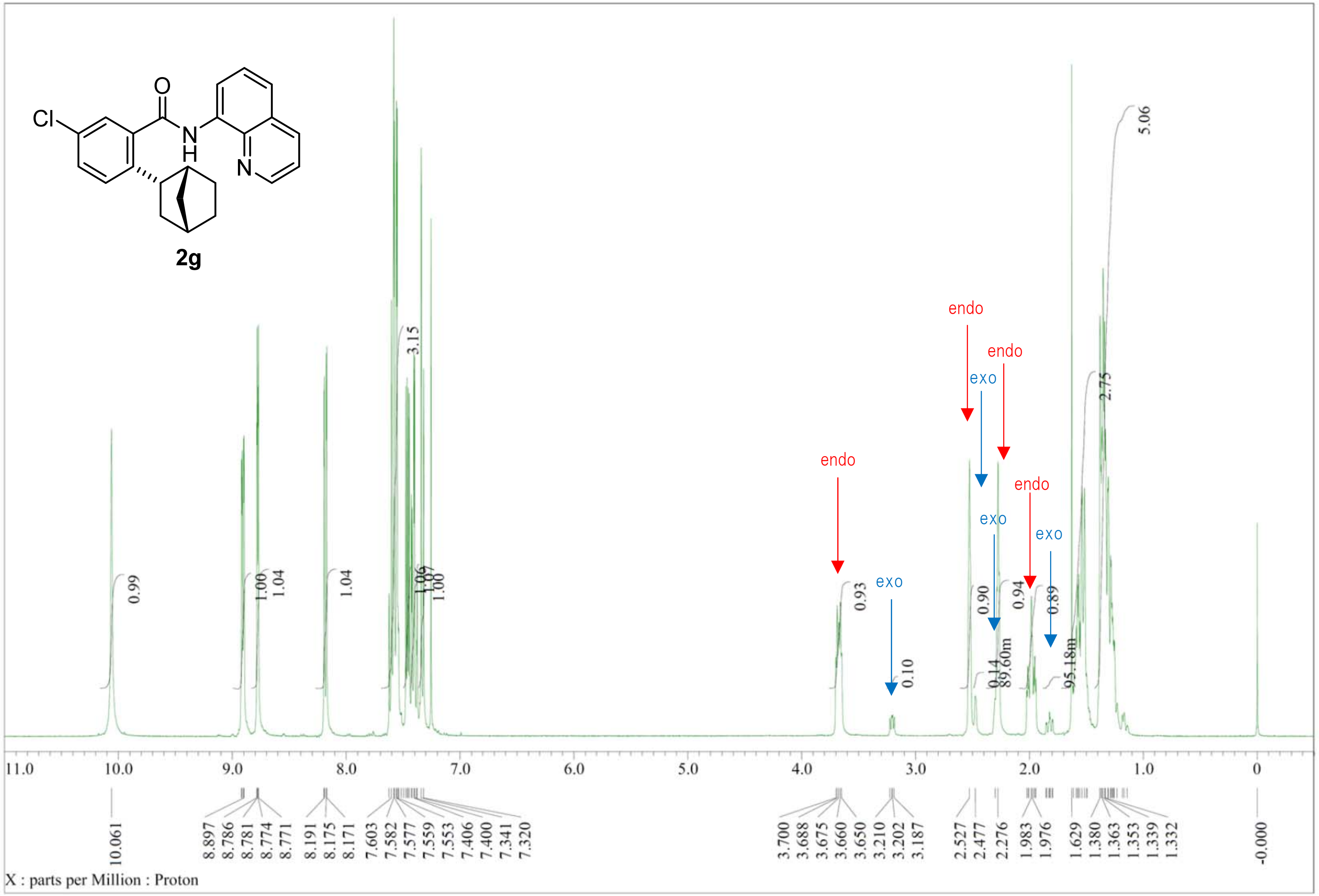

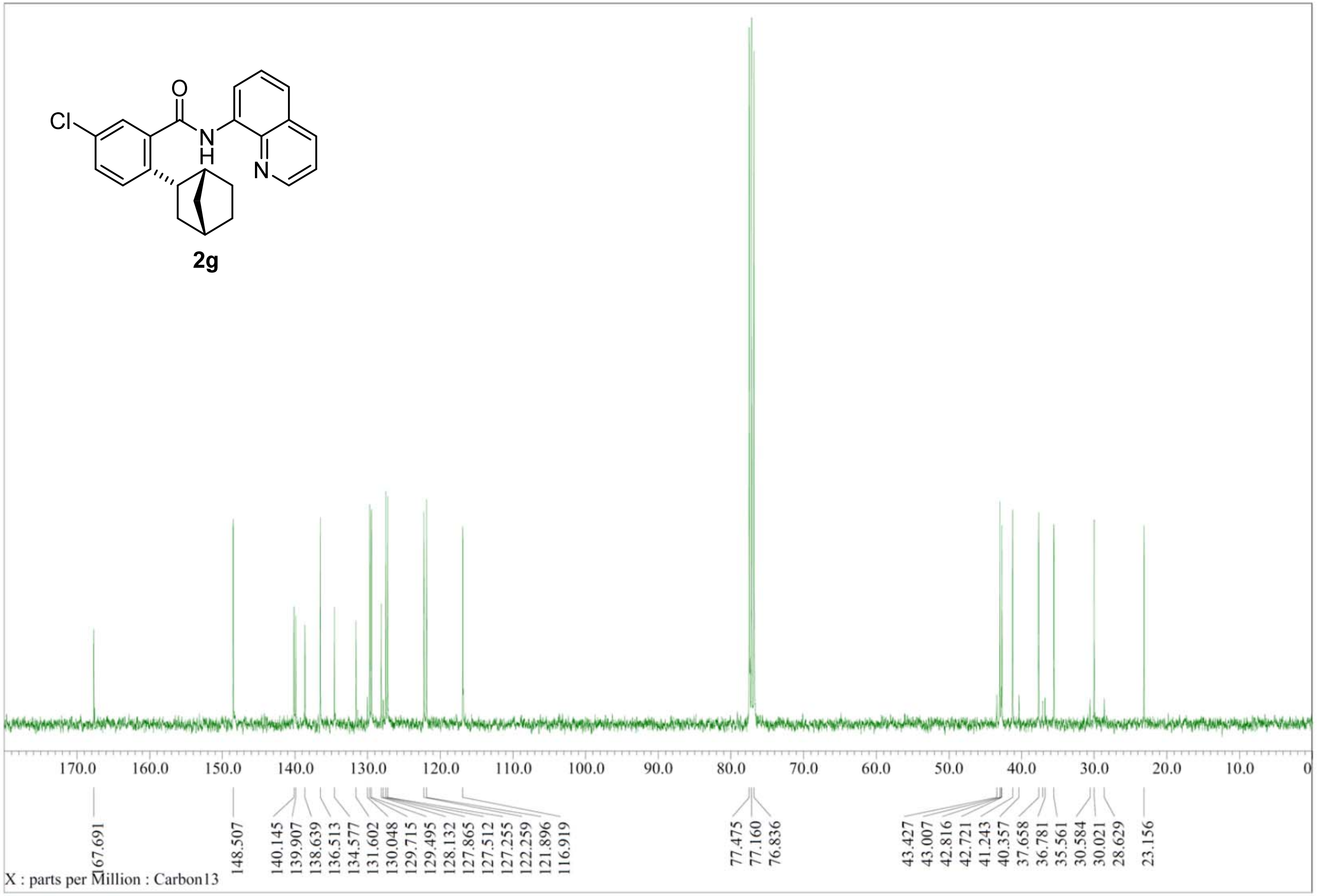
Cl
O
N
H
N
2g
170.0
160.0
150.0
140.0
130.0
120.0
110.0
100.0
90.0
80.0
70.0
60.0
50.0
40.0
30.0
20.0
10.0
0
167.691
148.507
140.145
139.907
138.639
136.513
134.577
131.602
130.048
129.715
129.495
128.132
127.865
127.512
127.255
122.259
121.896
116.919
77.475
77.160
76.836
43.427
43.007
42.816
42.721
41.243
40.357
37.658
36.781
35.561
30.584
30.021
28.629
23.156
X : parts per Million : Carbon13

Supplementary Figure 27: ^{1}H NMR, ^{13}C NMR spectra for 2h

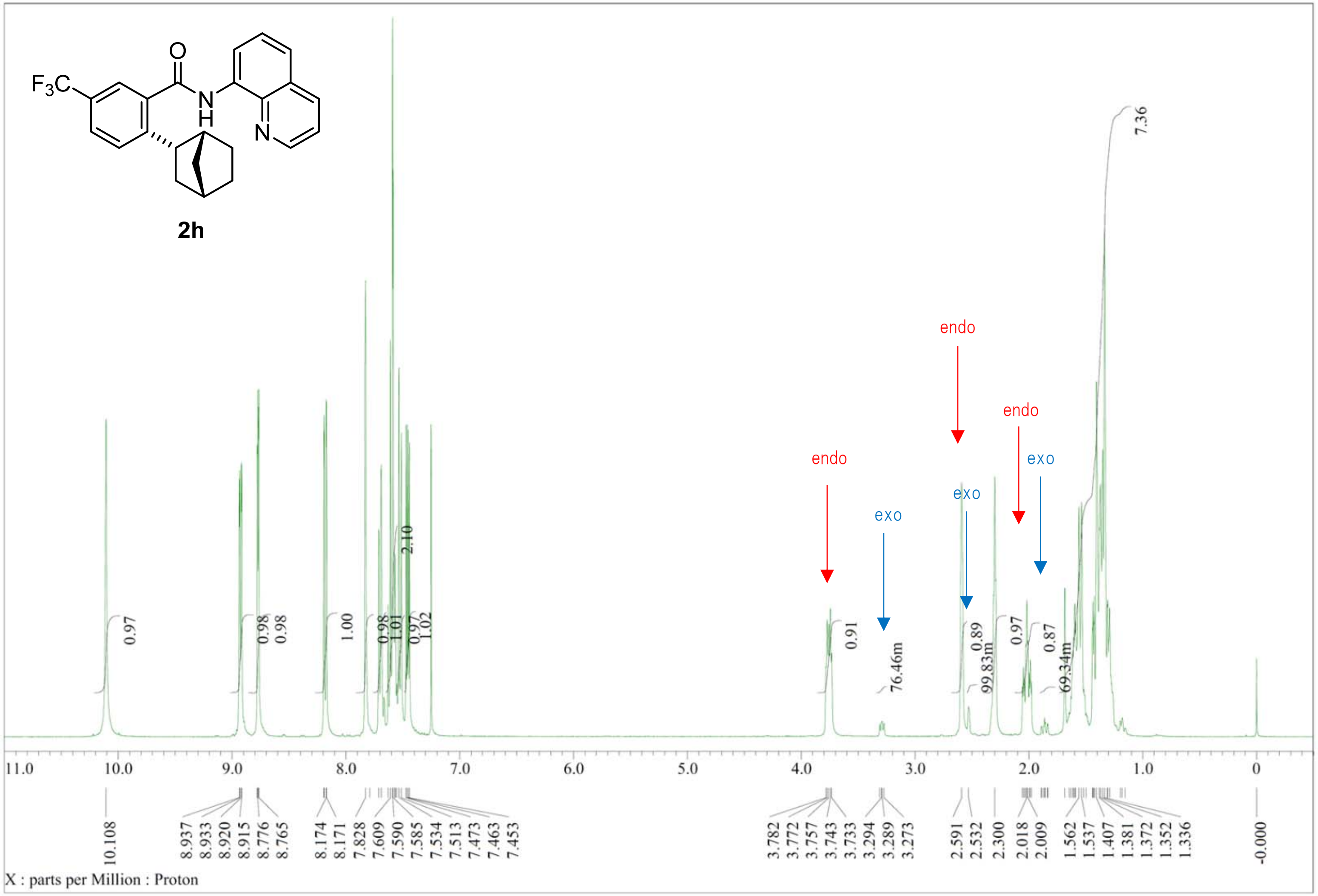

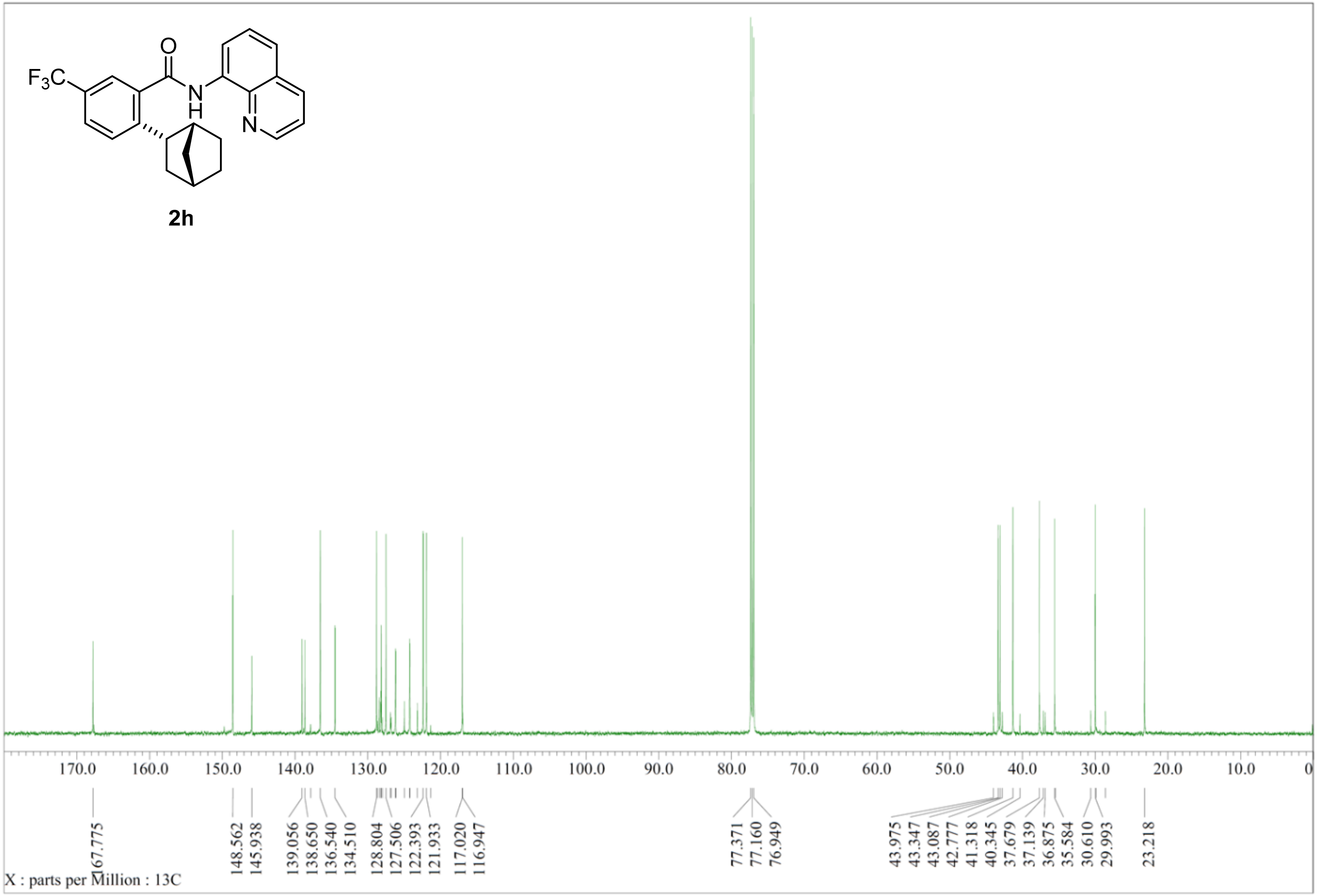
F3C
O
N
H
N
2h
170.0
160.0
150.0
140.0
130.0
120.0
110.0
100.0
90.0
80.0
70.0
60.0
50.0
40.0
30.0
20.0
10.0
0
167.775
148.562
145.938
139.056
138.650
136.540
134.510
128.804
127.506
122.393
121.933
117.020
116.947
77.371
77.160
76.949
43.975
43.347
43.087
42.777
41.318
40.345
37.679
37.139
36.875
35.584
30.610
29.993
23.218
X : parts per Million : 13C

Supplementary Figure 28: ^{1}H NMR, ^{13}C NMR spectra for 2i.

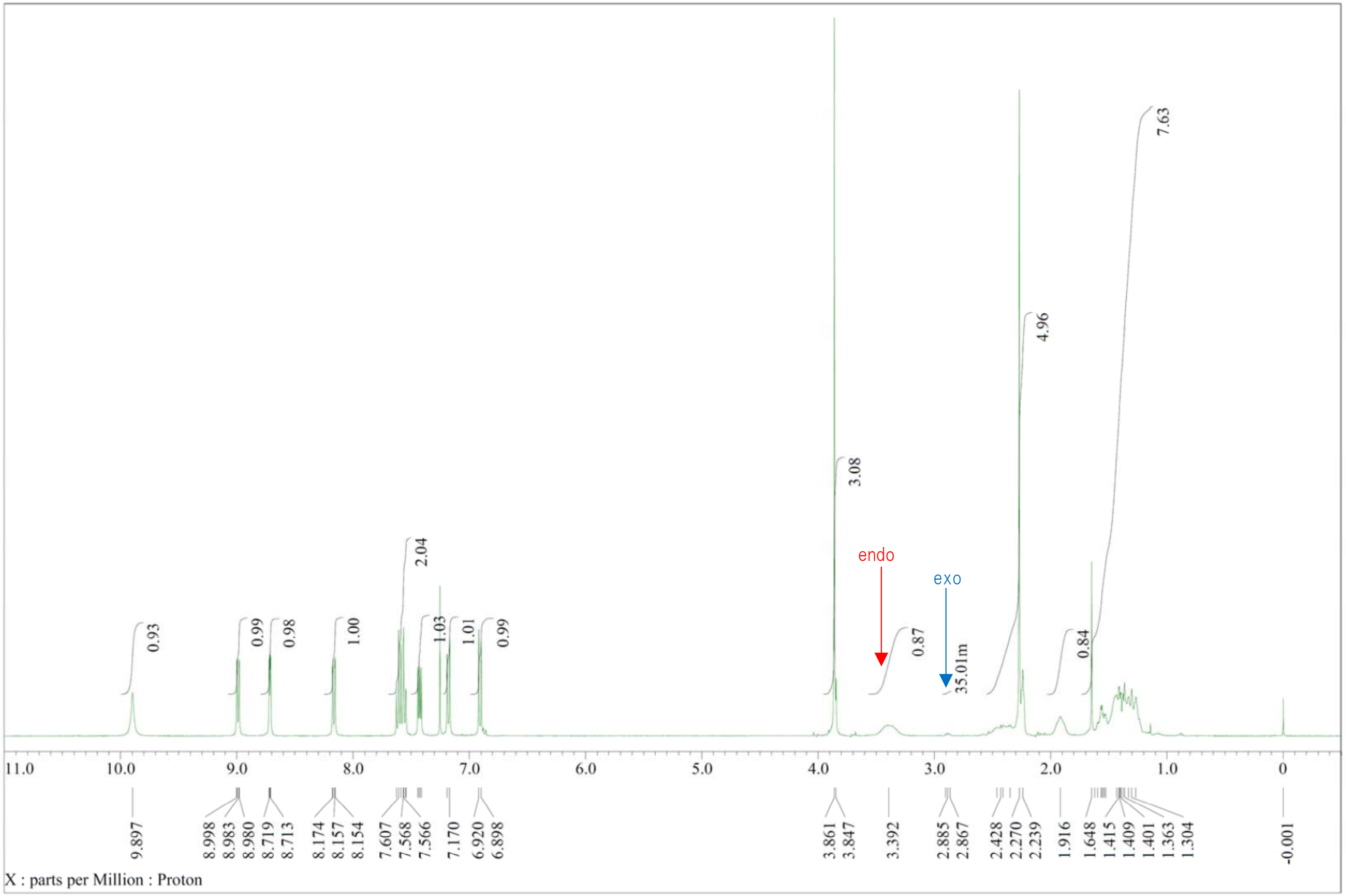

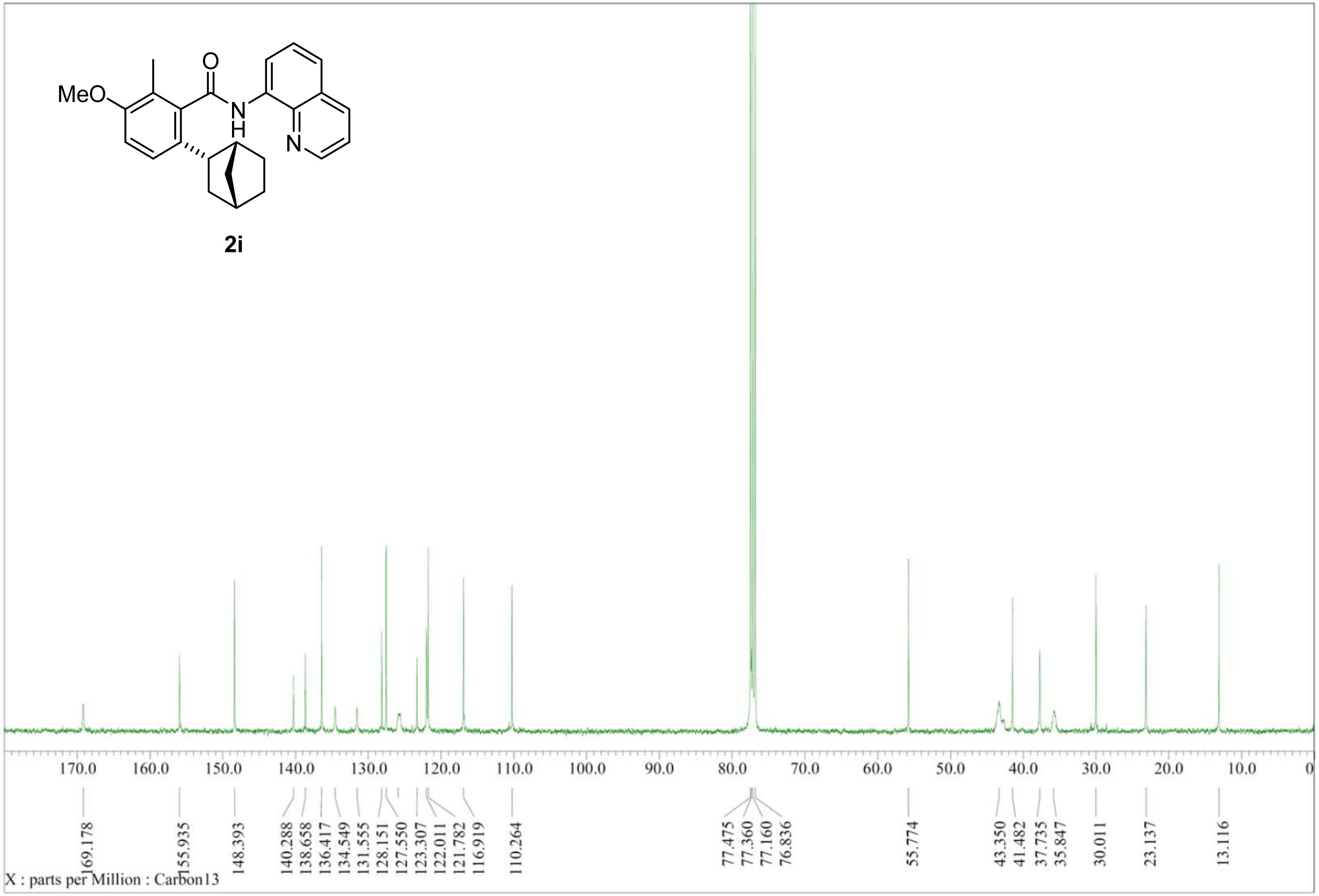
MeO
O
N
H
N
2i
170.0 160.0 150.0 140.0 130.0 120.0 110.0 100.0 90.0 80.0 70.0 60.0 50.0 40.0 30.0 20.0 10.0 0
169.178
155.935
148.393
140.288
138.658
136.417
134.549
131.555
128.151
127.550
123.307
122.011
121.782
116.919
110.264
77.475
77.360
77.160
76.836
55.774
43.350
41.482
37.735
35.847
30.011
23.137
13.116
X : parts per Million : Carbon13

Supplementary Figure 29: ^{1}H NMR, ^{13}C NMR spectra for 2j.

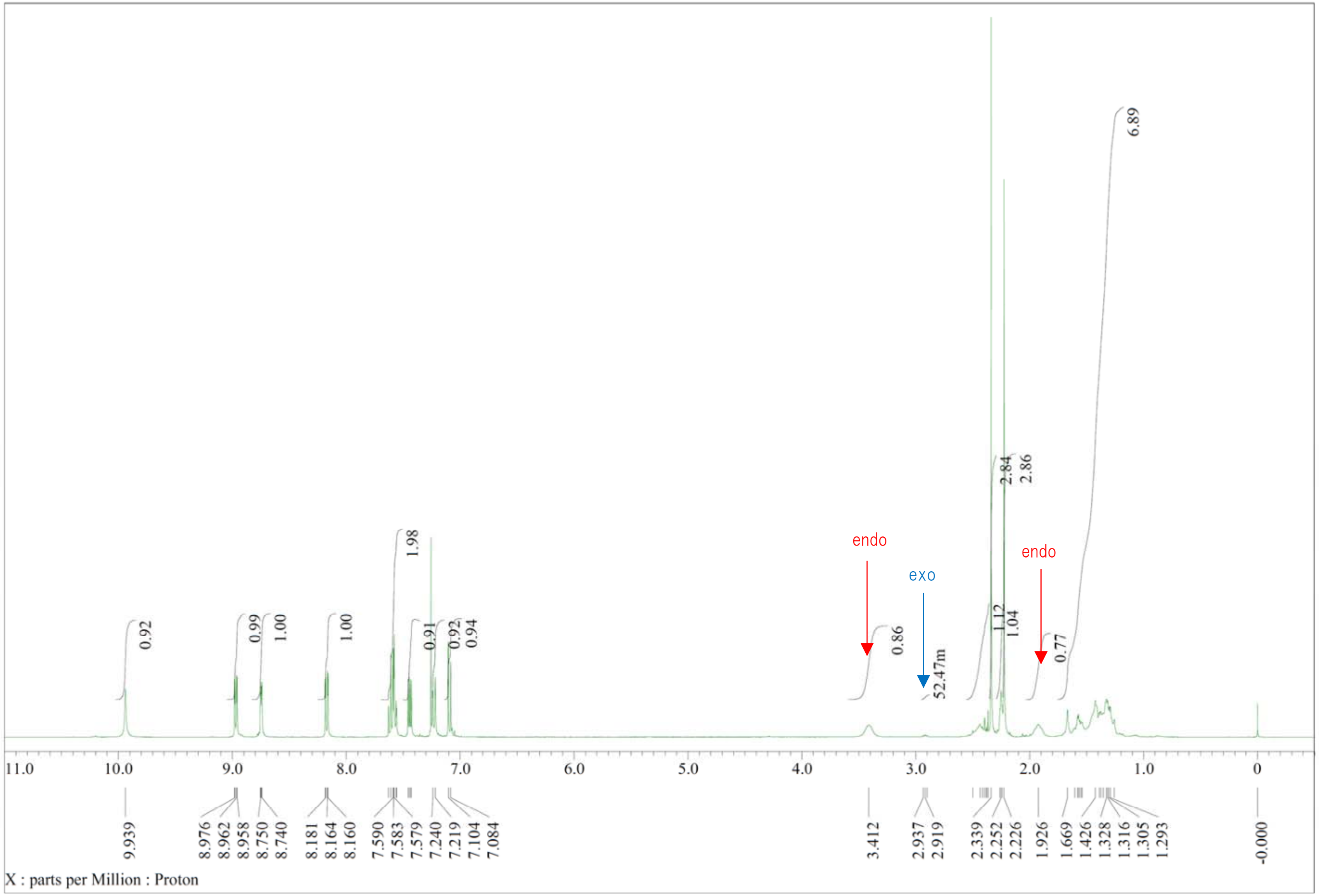

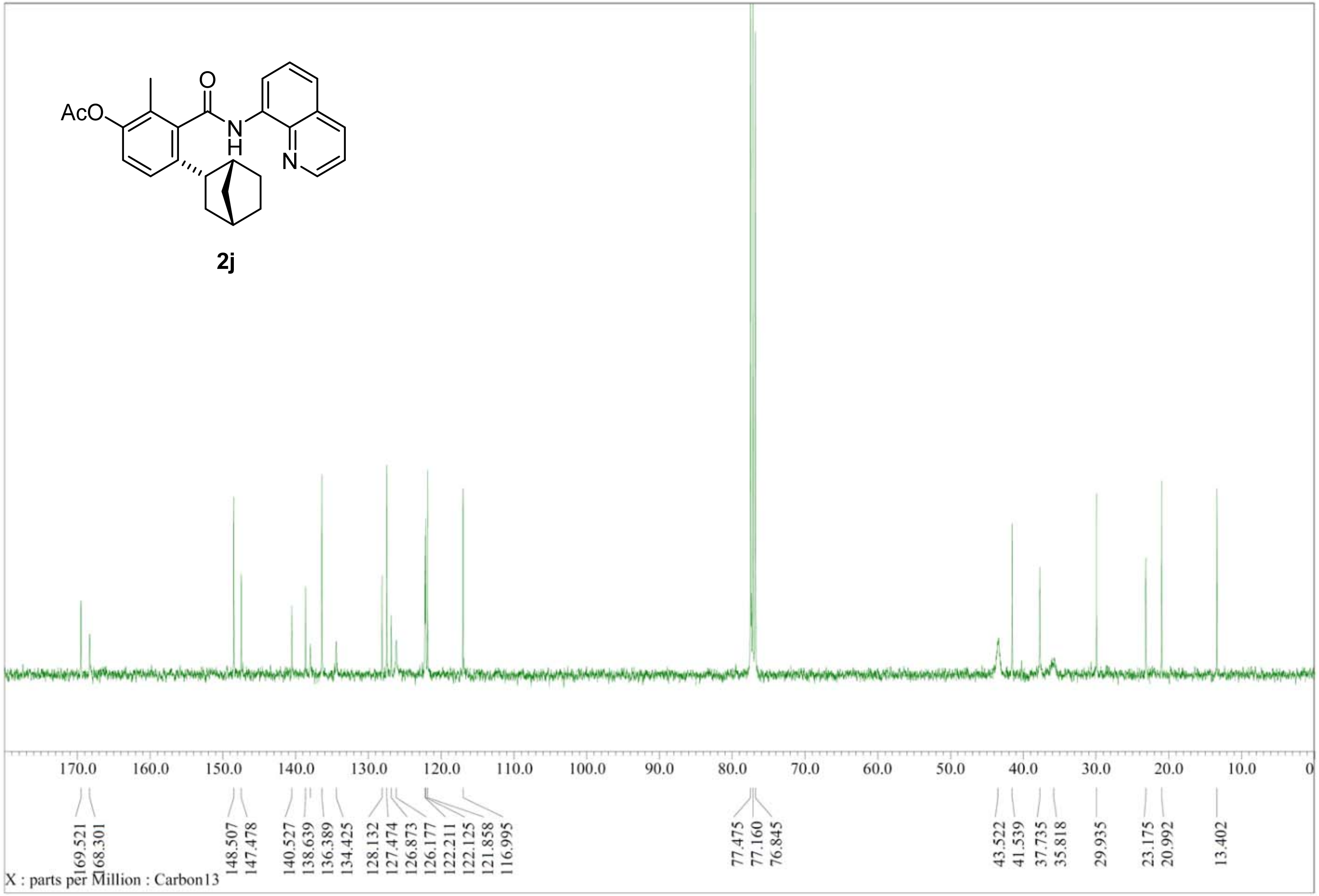
AcO
O
N
H
N
2j
170.0 160.0 150.0 140.0 130.0 120.0 110.0 100.0 90.0 80.0 70.0 60.0 50.0 40.0 30.0 20.0 10.0 0
169.521
168.301
148.507
147.478
140.527
138.639
136.389
134.425
128.132
127.474
126.873
126.177
122.211
122.125
121.858
116.995
77.475
77.160
76.845
43.522
41.539
37.735
35.818
29.935
23.175
20.992
13.402
X : parts per Million : Carbon13

Supplementary Figure 30: ^{1}H NMR, ^{13}C NMR spectra for 2k.

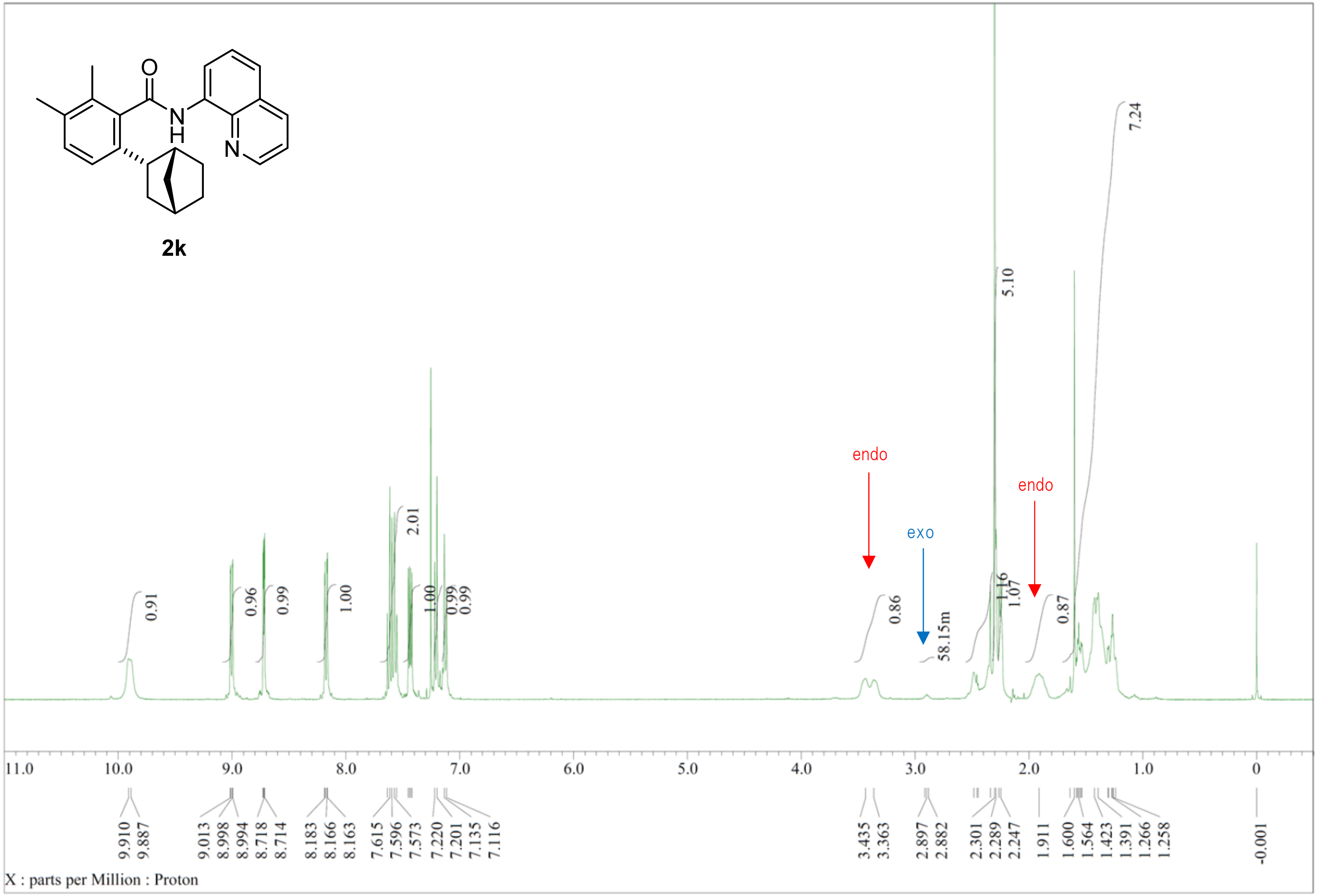

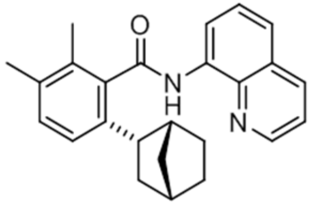

2k

Supplementary Figure 31: ^{1}H NMR, ^{13}C NMR spectra for 2l

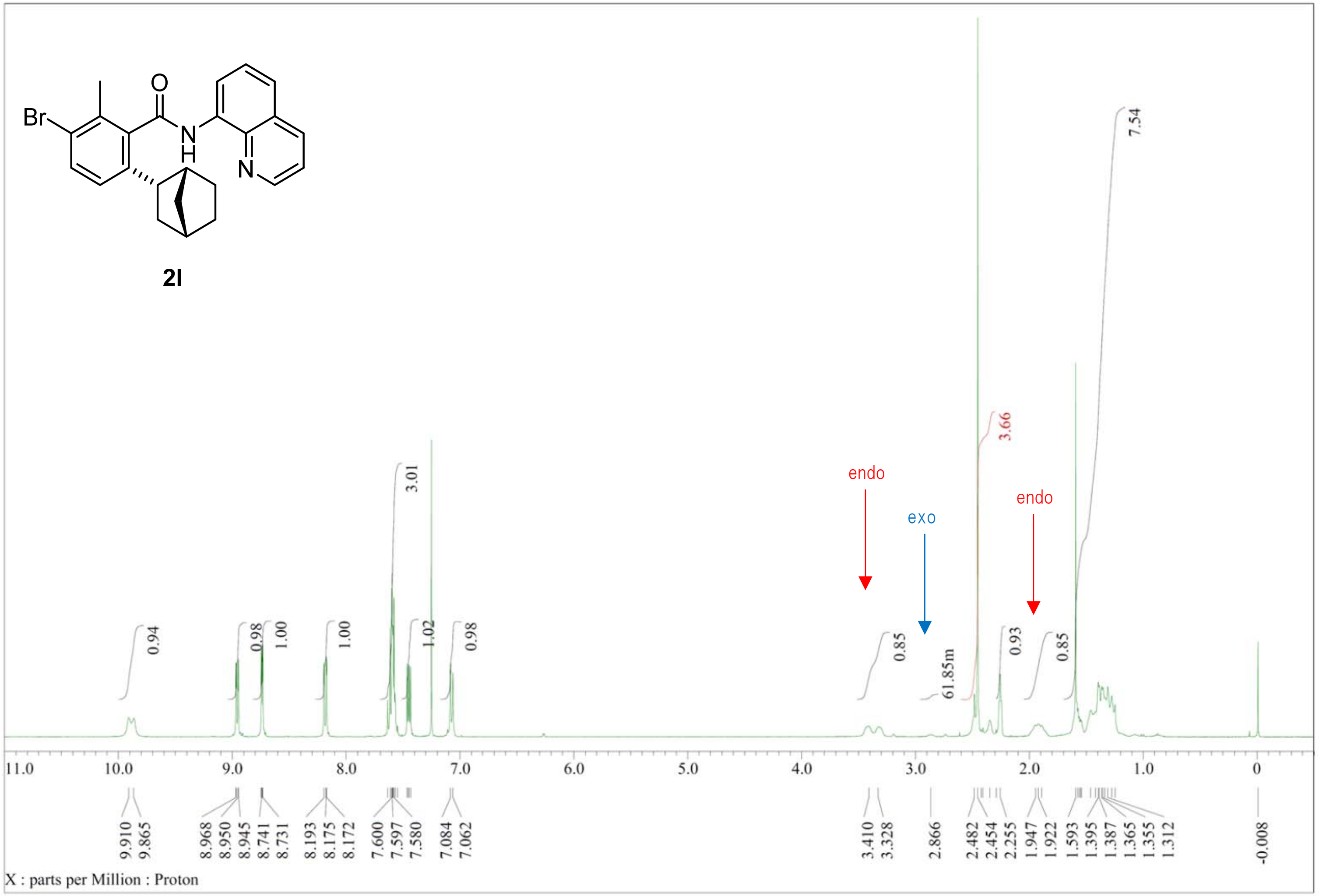

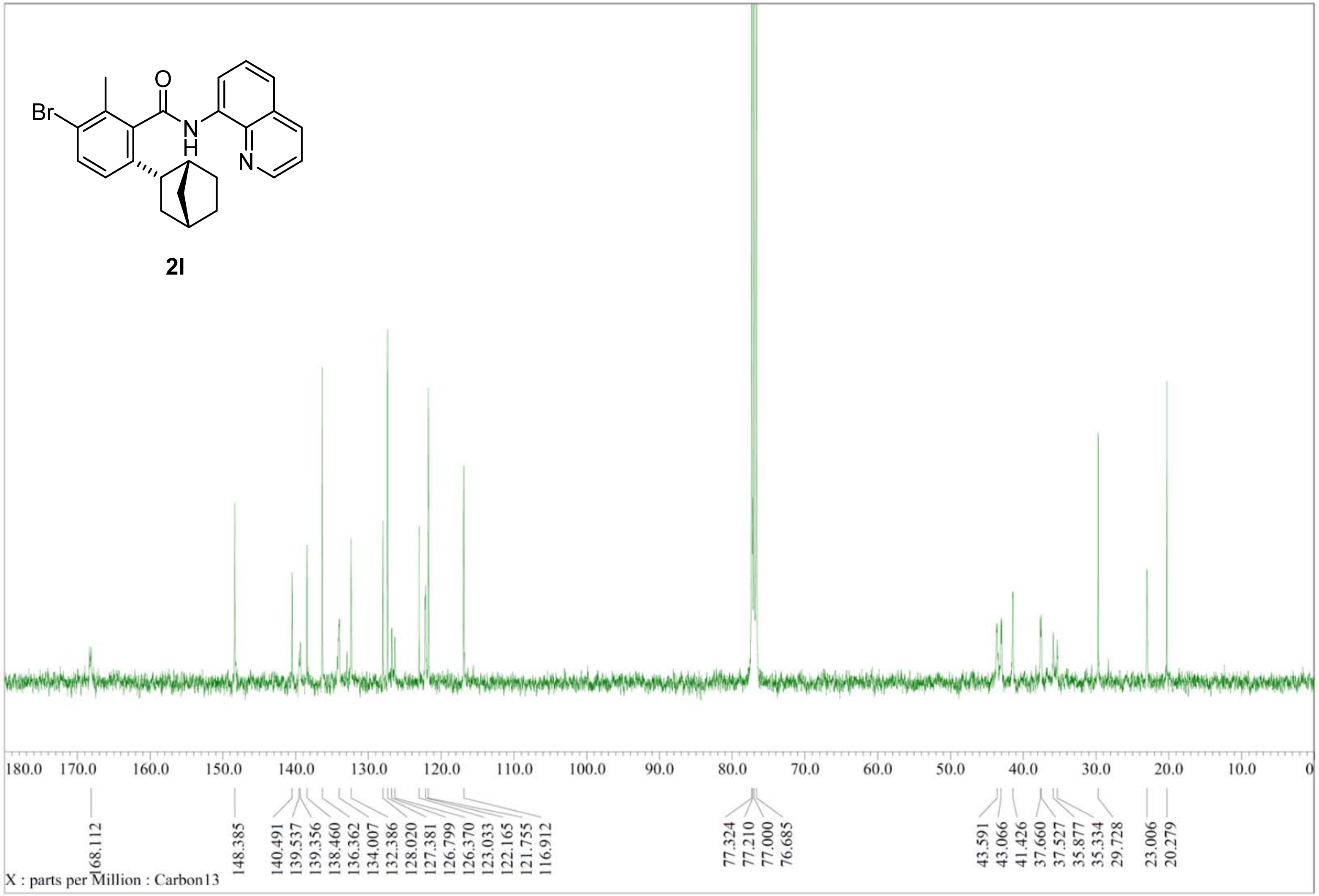

Br
O
N
H
N
2l
180.0 170.0 160.0 150.0 140.0 130.0 120.0 110.0 100.0 90.0 80.0 70.0 60.0 50.0 40.0 30.0 20.0 10.0 0
168.112
148.385
140.491
139.537
139.356
138.460
136.362
134.007
132.386
128.020
127.381
126.799
126.370
123.033
122.165
121.755
116.912
77.324
77.210
77.000
76.685
43.591
43.066
41.426
37.660
37.527
35.877
35.334
29.728
23.006
20.279
X : parts per Million : Carbon13

Supplementary Figure 32: ^{1}H NMR, ^{13}C NMR spectra for 2m.

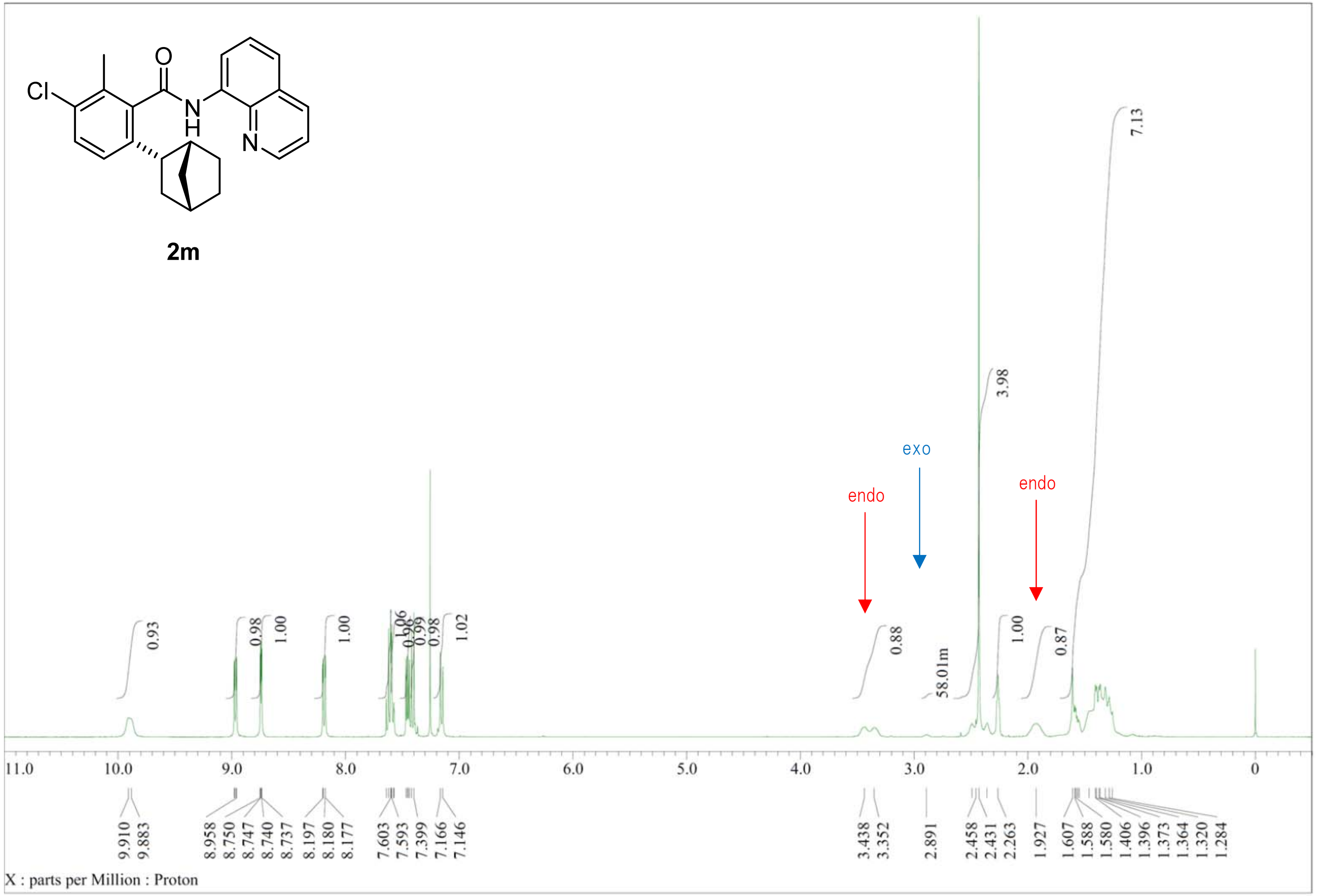

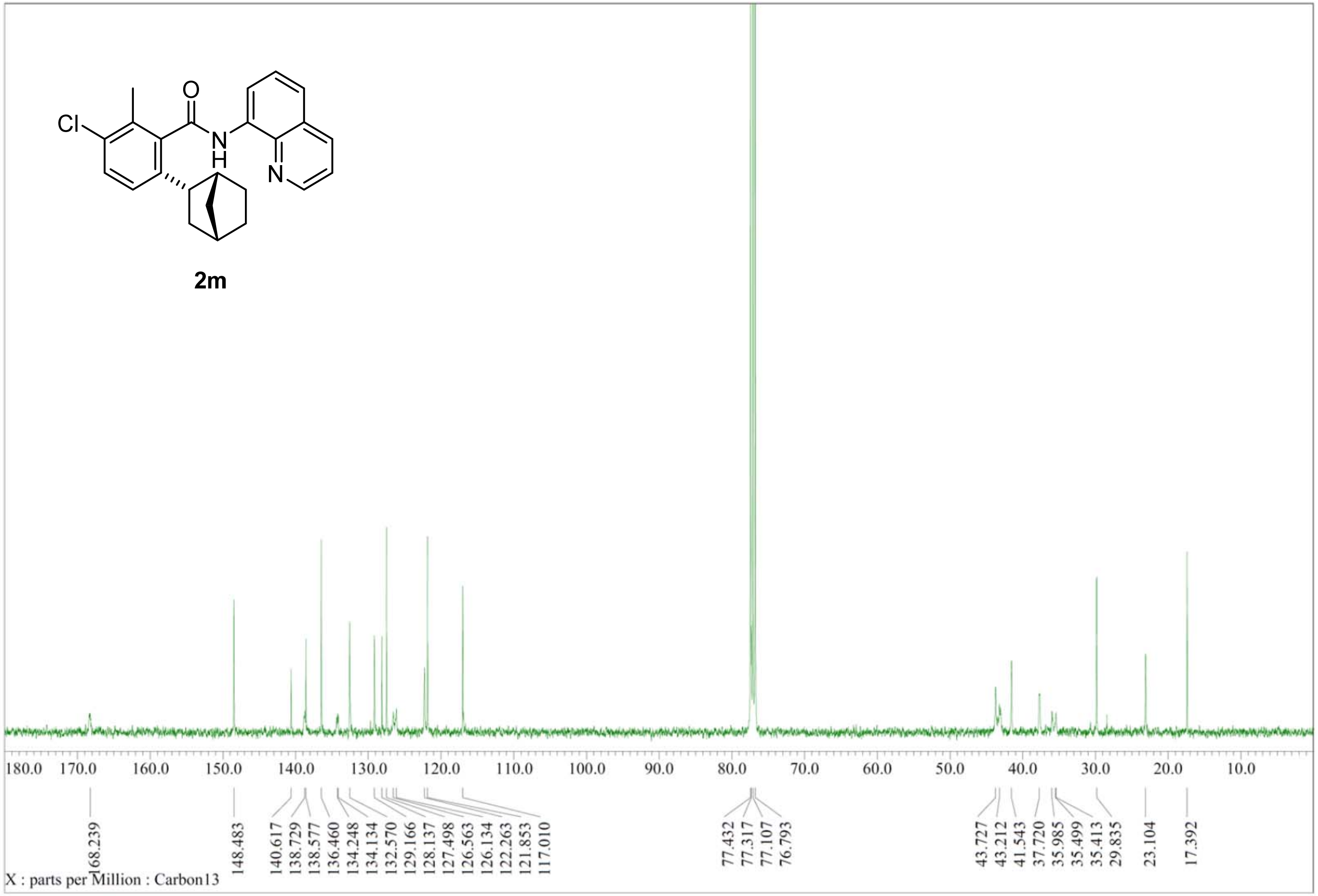
Cl
O
N
H
N
2m
180.0 170.0 160.0 150.0 140.0 130.0 120.0 110.0 100.0 90.0 80.0 70.0 60.0 50.0 40.0 30.0 20.0 10.0
168.239
148.483
140.617
138.729
138.577
136.460
134.248
134.134
132.570
129.166
128.137
127.498
126.563
126.134
122.263
121.853
117.010
77.432
77.317
77.107
76.793
43.727
43.212
41.543
37.720
35.985
35.499
35.413
29.835
23.104
17.392
X : parts per Million : Carbon13

Supplementary Figure 33: ^{1}H NMR, ^{13}C NMR spectra for 2n

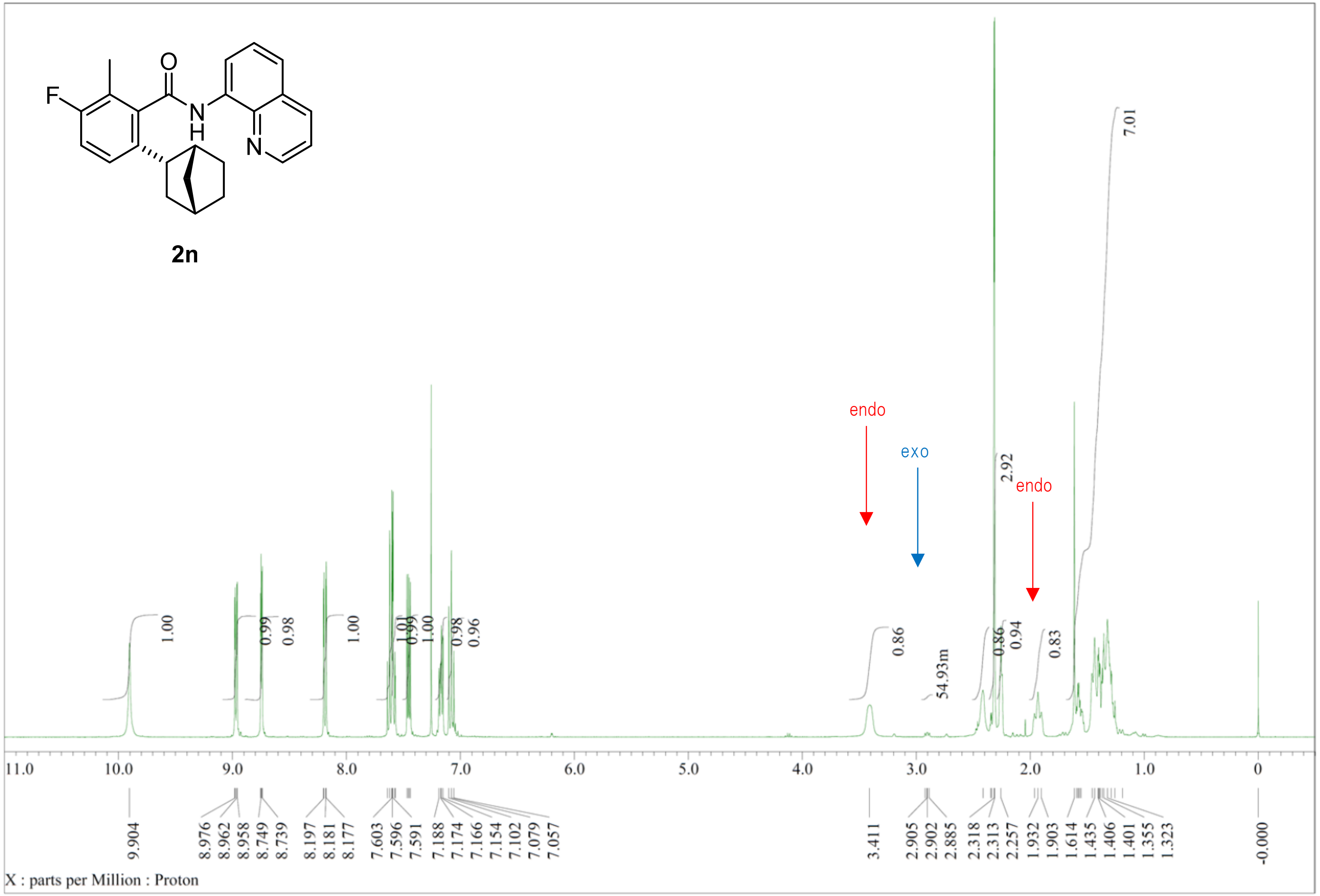

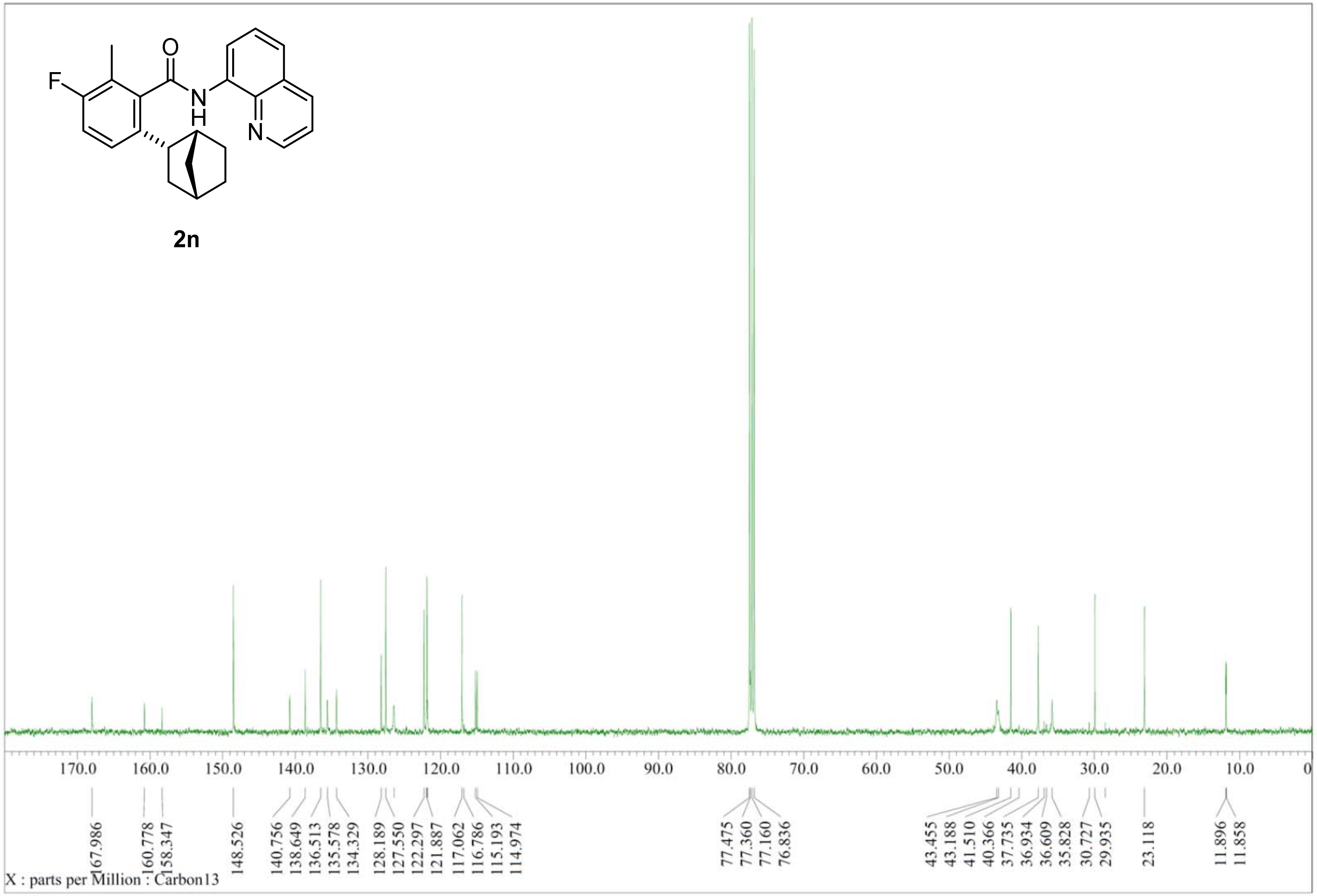
2n
170.0 160.0 150.0 140.0 130.0 120.0 110.0 100.0 90.0 80.0 70.0 60.0 50.0 40.0 30.0 20.0 10.0 0
167.986
160.778
158.347
148.526
140.756
138.649
136.513
135.578
134.329
128.189
127.550
122.297
121.887
117.062
116.786
115.193
114.974
77.475
77.360
77.160
76.836
43.455
43.188
41.510
40.366
37.735
36.934
36.609
35.828
30.727
29.935
23.118
11.896
11.858
X : parts per Million : Carbon13

Supplementary Figure 34: ^{1}H NMR, ^{13}C NMR spectra for 2o

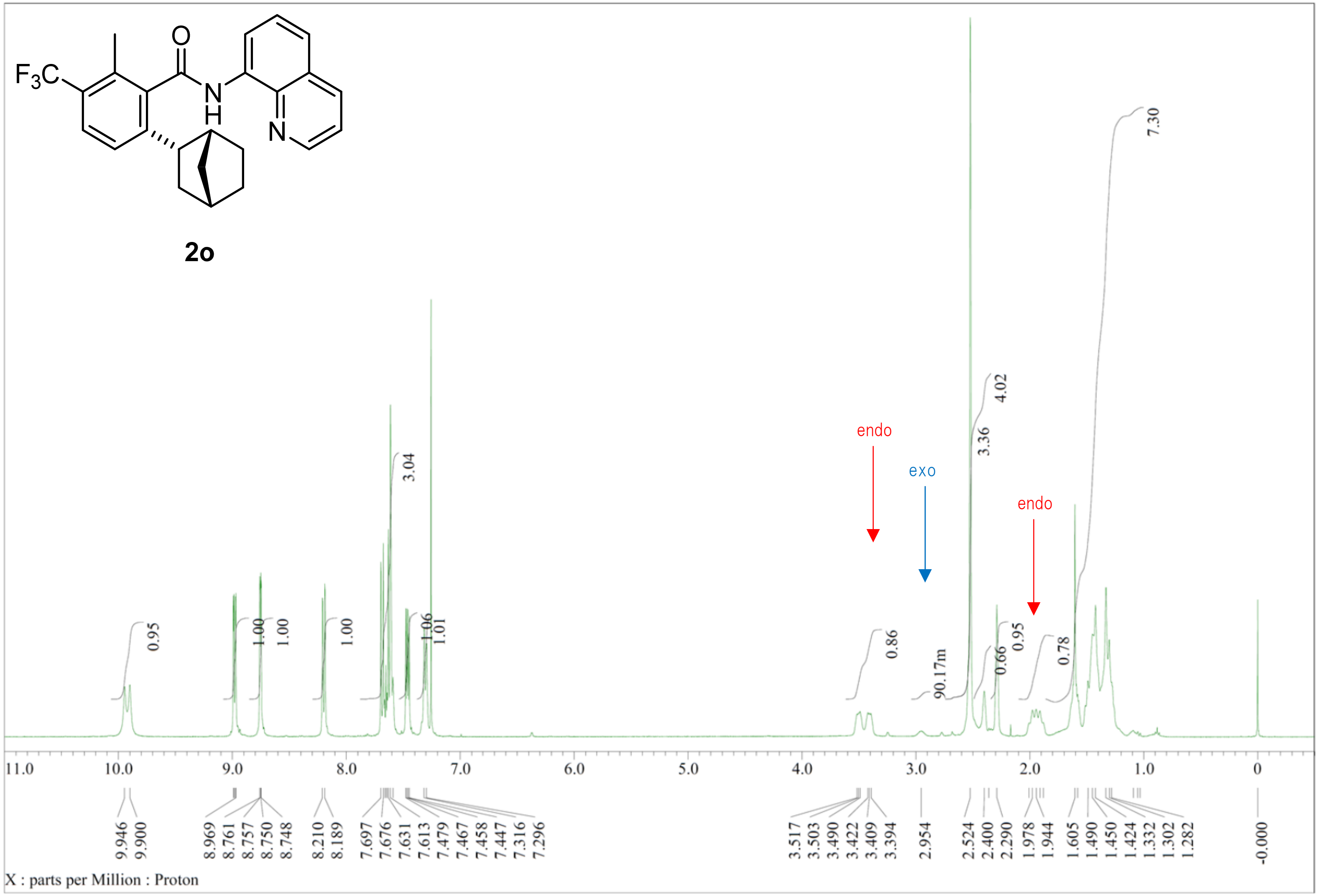

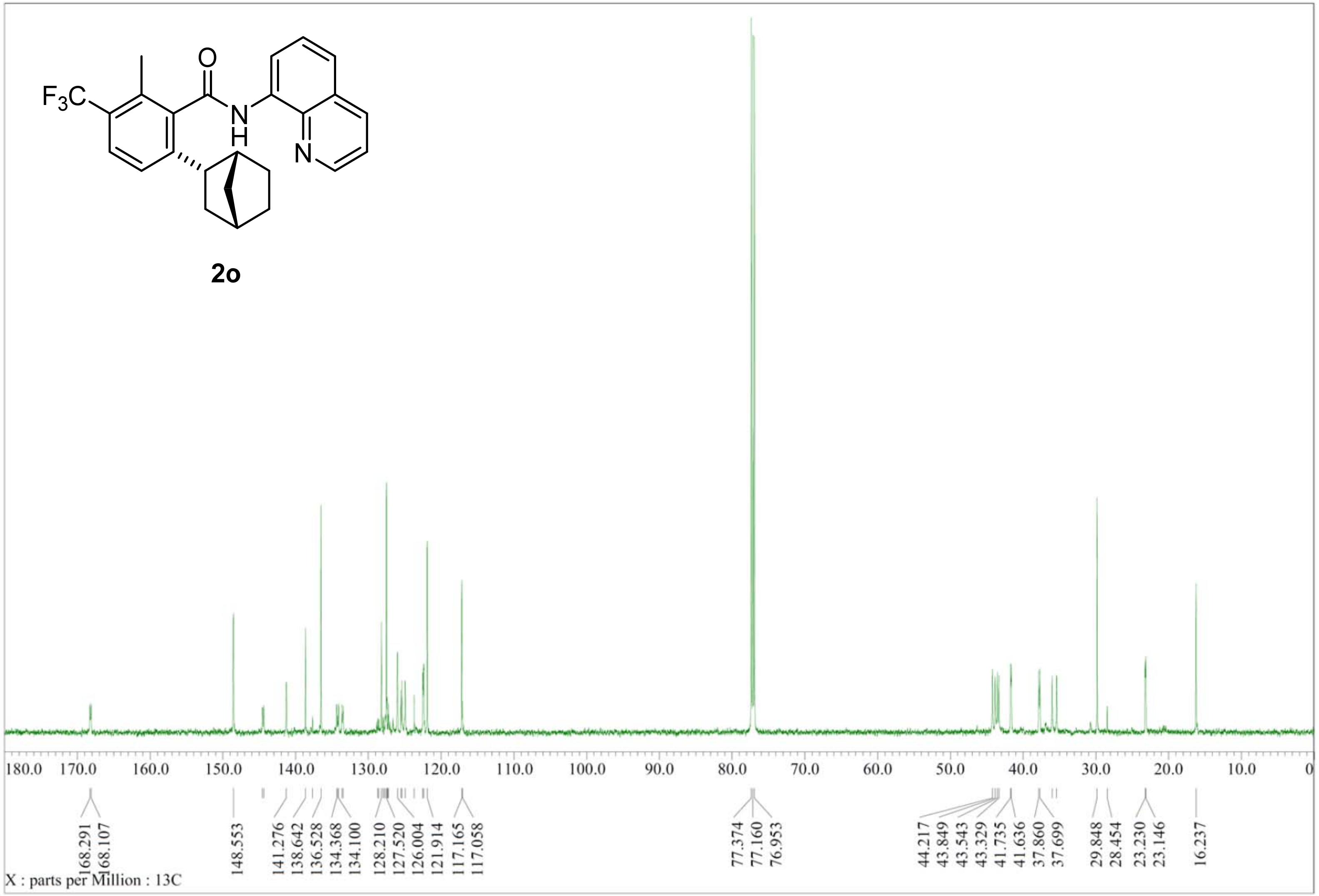

F3C
O
N
H
N
2o
180.0 170.0 160.0 150.0 140.0 130.0 120.0 110.0 100.0 90.0 80.0 70.0 60.0 50.0 40.0 30.0 20.0 10.0 0
168.291
168.107
148.553
141.276
138.642
136.528
134.368
134.100
128.210
127.520
126.004
121.914
117.165
117.058
77.374
77.160
76.953
44.217
43.849
43.543
43.329
41.735
41.636
37.860
37.699
29.848
28.454
23.230
23.146
16.237
X : parts per Million : 13C

Supplementary Figure 35: ^{1}H NMR, ^{13}C NMR spectra for 2p

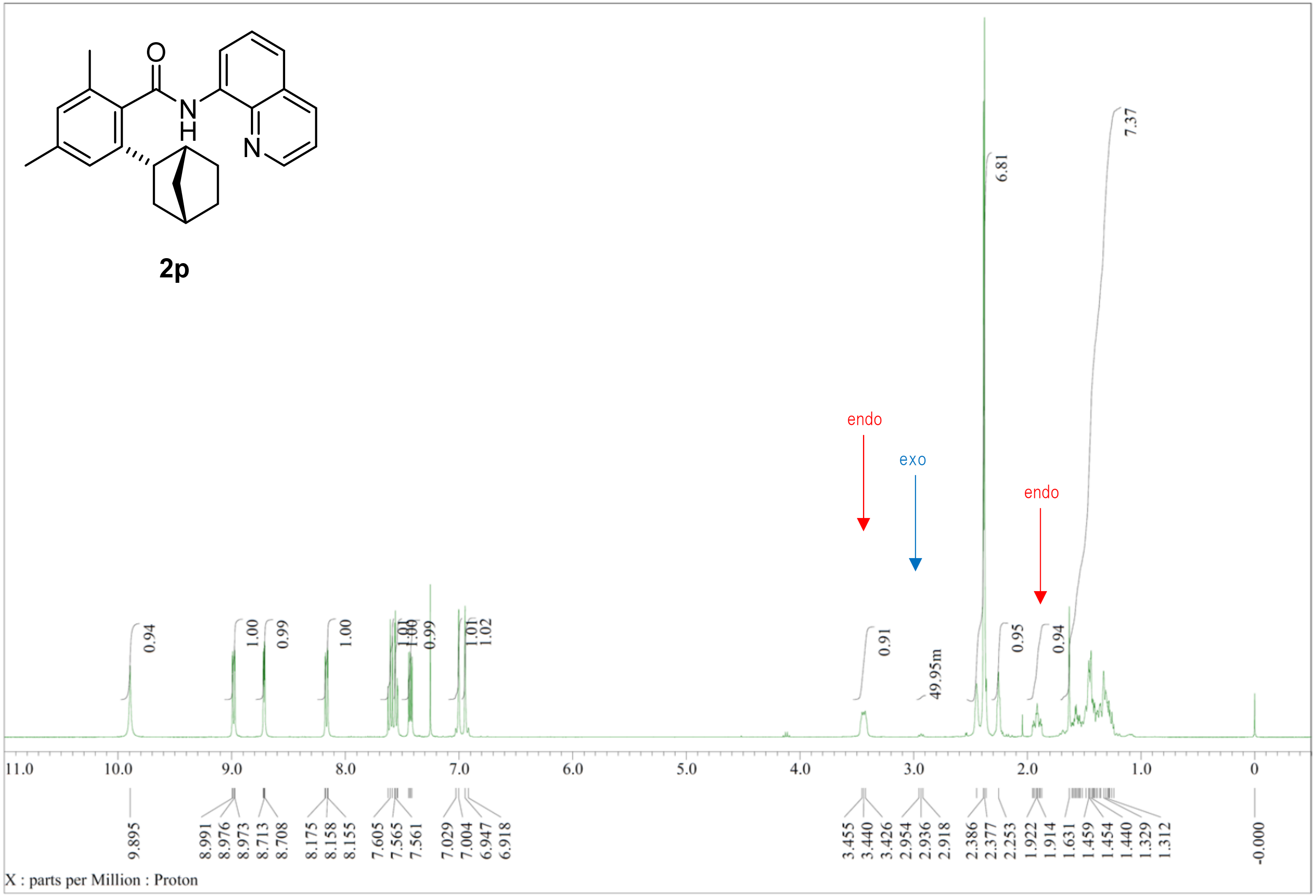

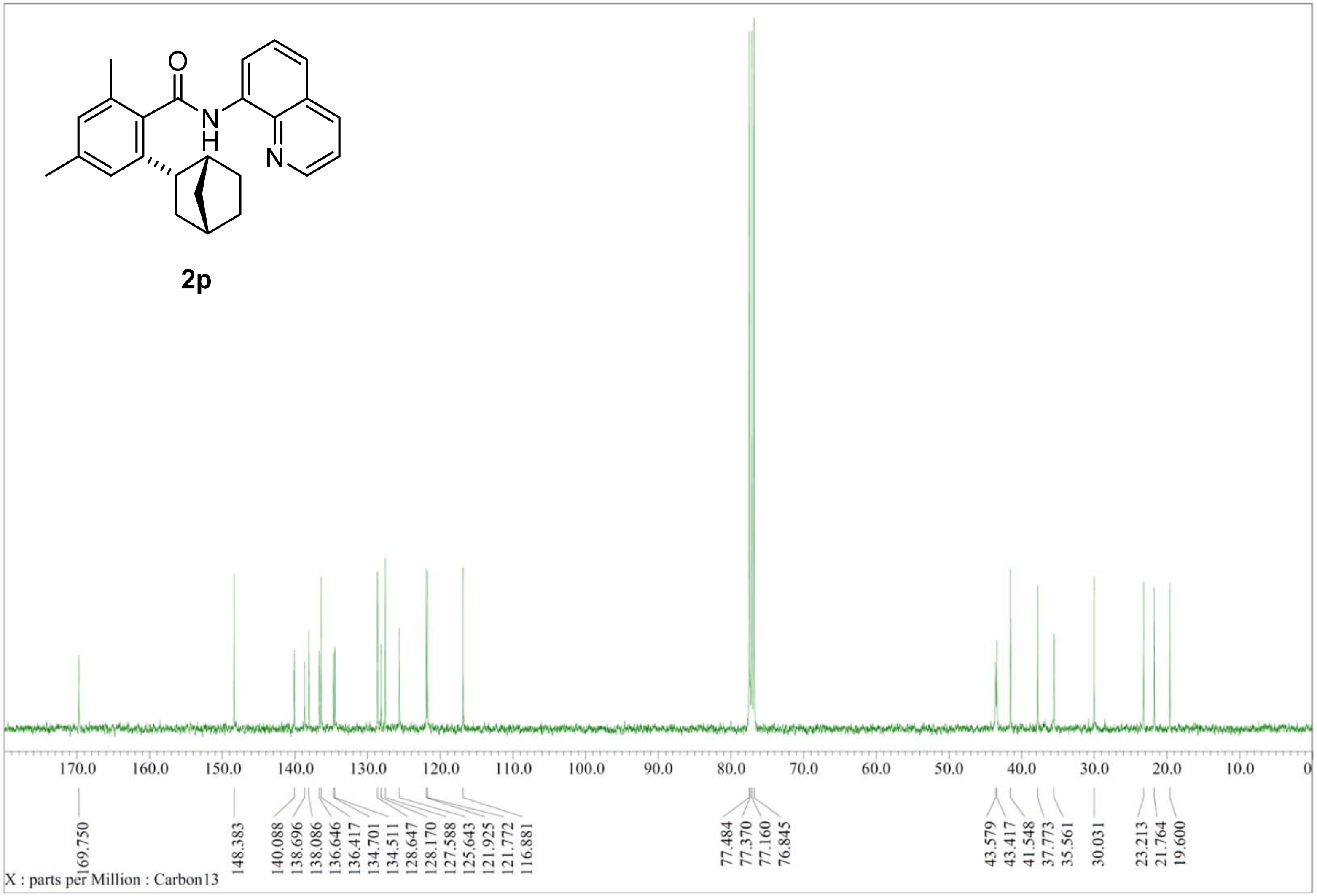
2p
O
N
H
N
170.0 160.0 150.0 140.0 130.0 120.0 110.0 100.0 90.0 80.0 70.0 60.0 50.0 40.0 30.0 20.0 10.0 0
169.750
148.383
140.088
138.696
138.086
136.646
136.417
134.701
134.511
128.647
128.170
127.588
125.643
121.925
121.772
116.881
77.484
77.370
77.160
76.845
43.579
43.417
41.548
37.773
35.561
30.031
23.213
21.764
19.600
X : parts per Million : Carbon13

Supplementary Figure 36: ^{1}H NMR, ^{13}C NMR spectra for 2q

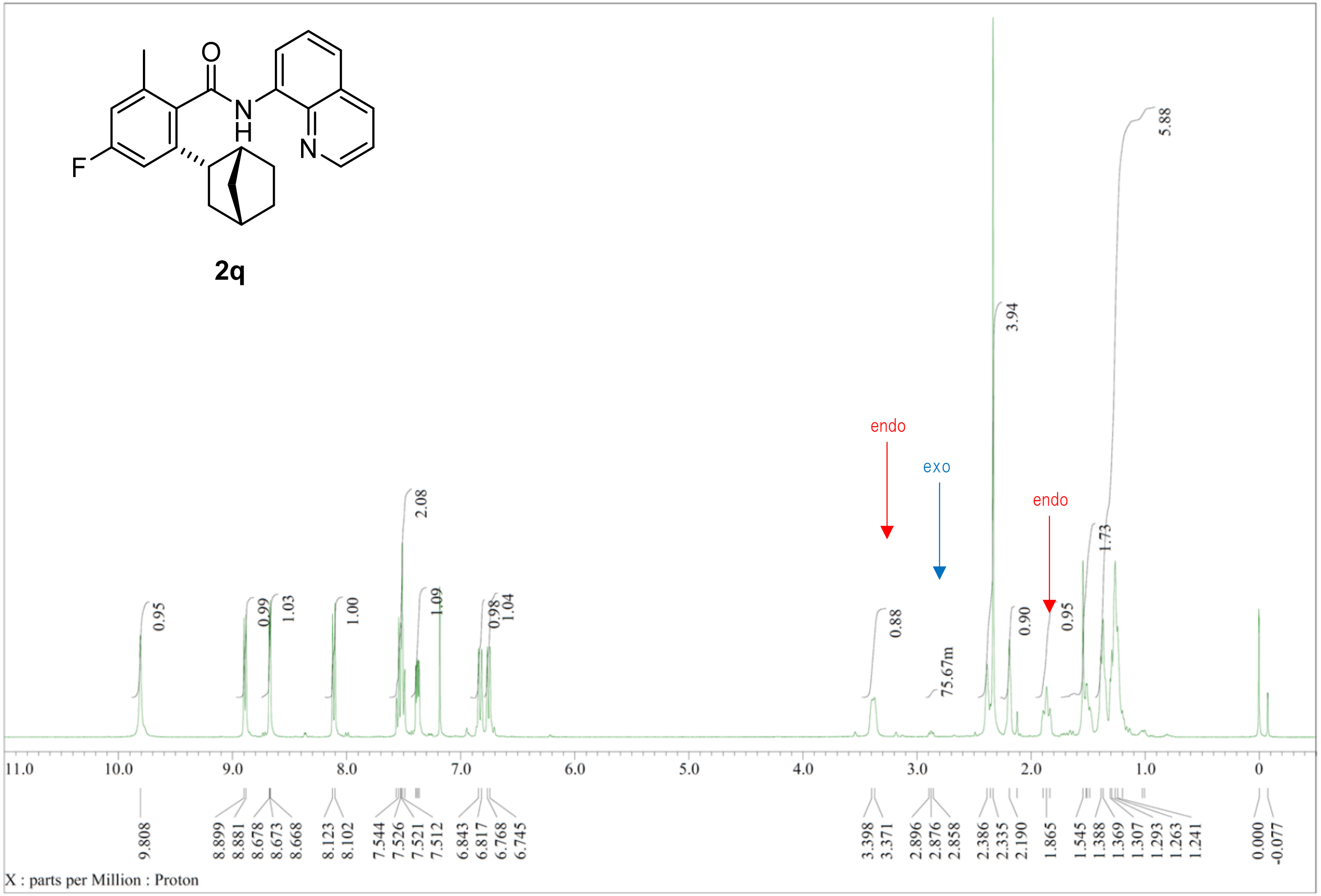

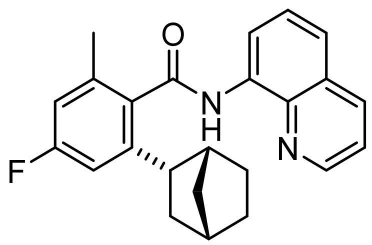

2q

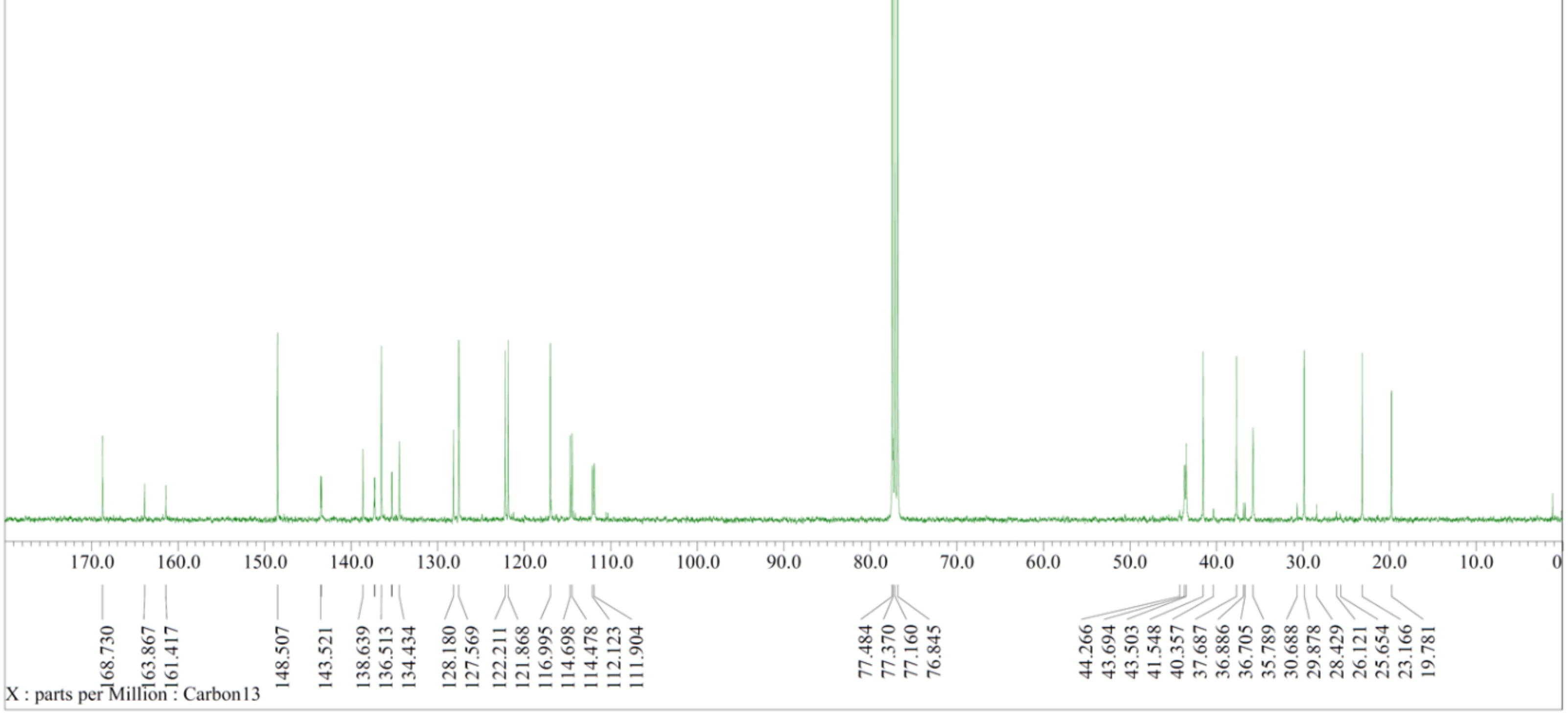

170.0
160.0
150.0
140.0
130.0
120.0
110.0
100.0
90.0
80.0
70.0
60.0
50.0
40.0
30.0
20.0
10.0
168.730
163.867
161.417
148.507
143.521
138.639
136.513
134.434
128.180
127.569
122.211
121.868
116.995
114.698
114.478
112.123
111.904
77.484
77.370
77.160
76.845
44.266
43.694
43.503
41.548
40.357
37.687
36.886
36.705
35.789
30.688
29.878
28.429
26.121
25.654
23.166
19.781
X : parts per Million : Carbon13

Supplementary Figure 37: ^{1}H NMR, ^{13}C NMR spectra for 2r.

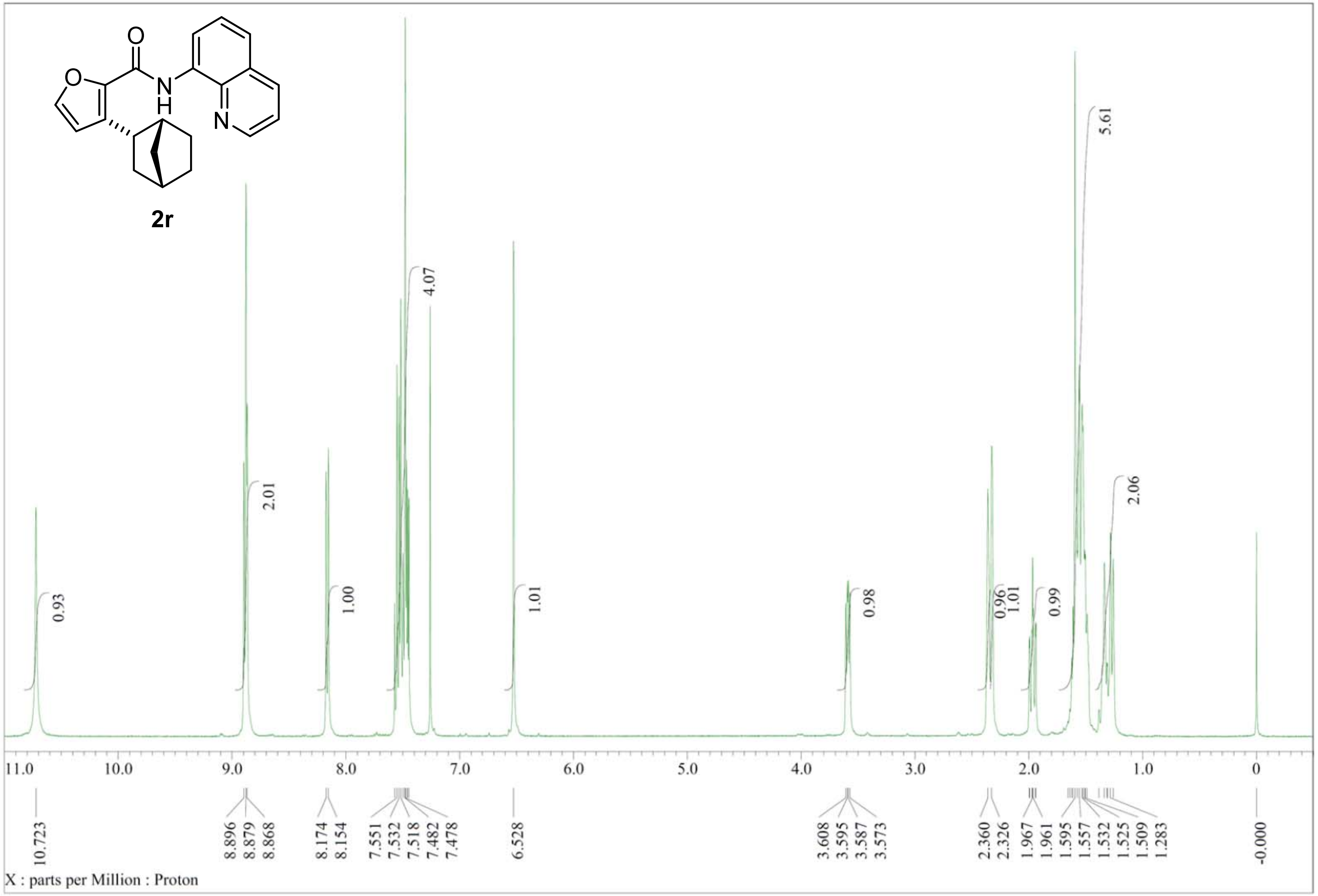

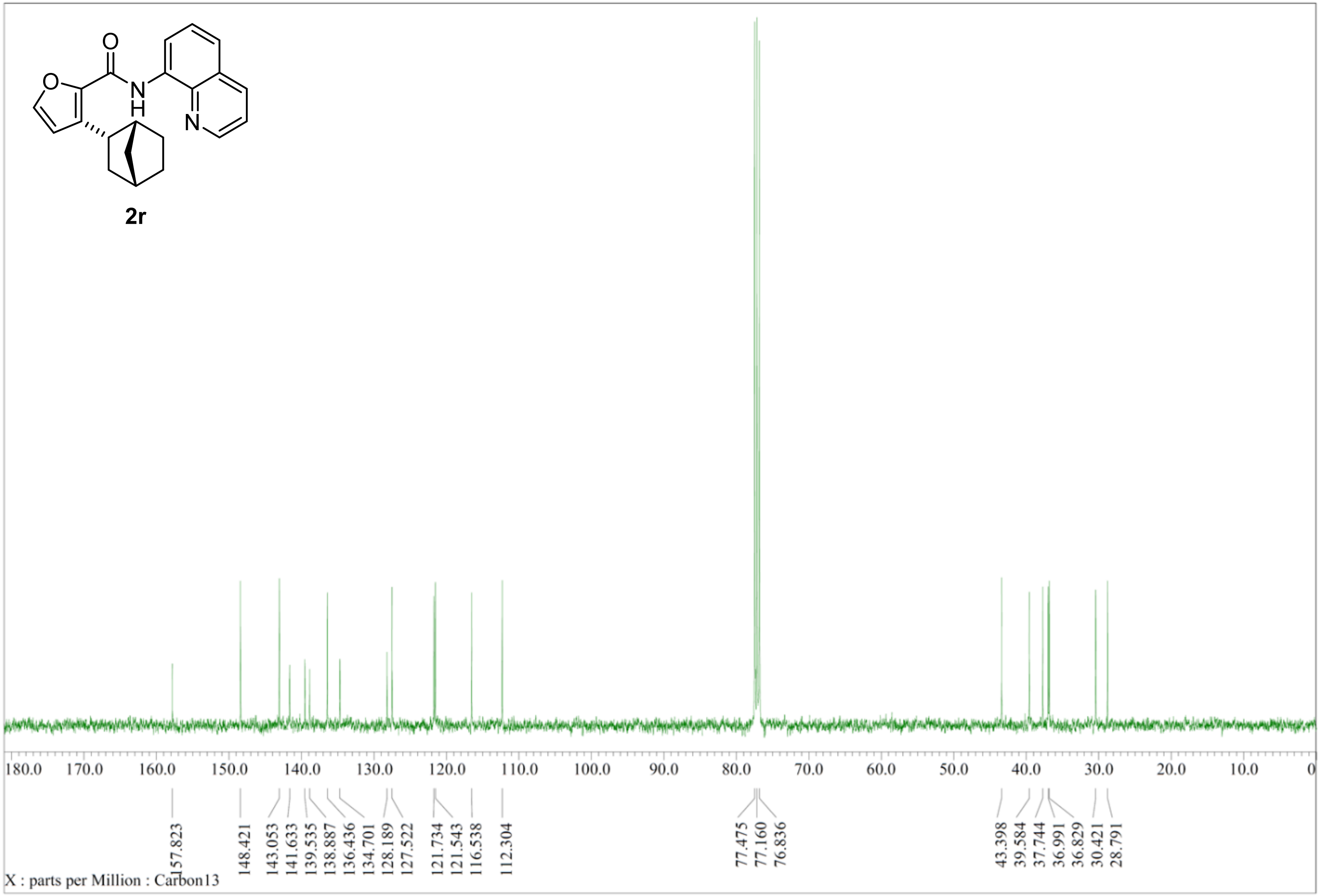
O
N
H
N
2r
180.0 170.0 160.0 150.0 140.0 130.0 120.0 110.0 100.0 90.0 80.0 70.0 60.0 50.0 40.0 30.0 20.0 10.0 0
157.823
148.421
143.053
141.633
139.535
138.887
136.436
134.701
128.189
127.522
121.734
121.543
116.538
112.304
77.475
77.160
76.836
43.398
39.584
37.744
36.991
36.829
30.421
28.791
X : parts per Million : Carbon13

Supplementary Figure 38: ^{1}H NMR, ^{13}C NMR spectra for 2s

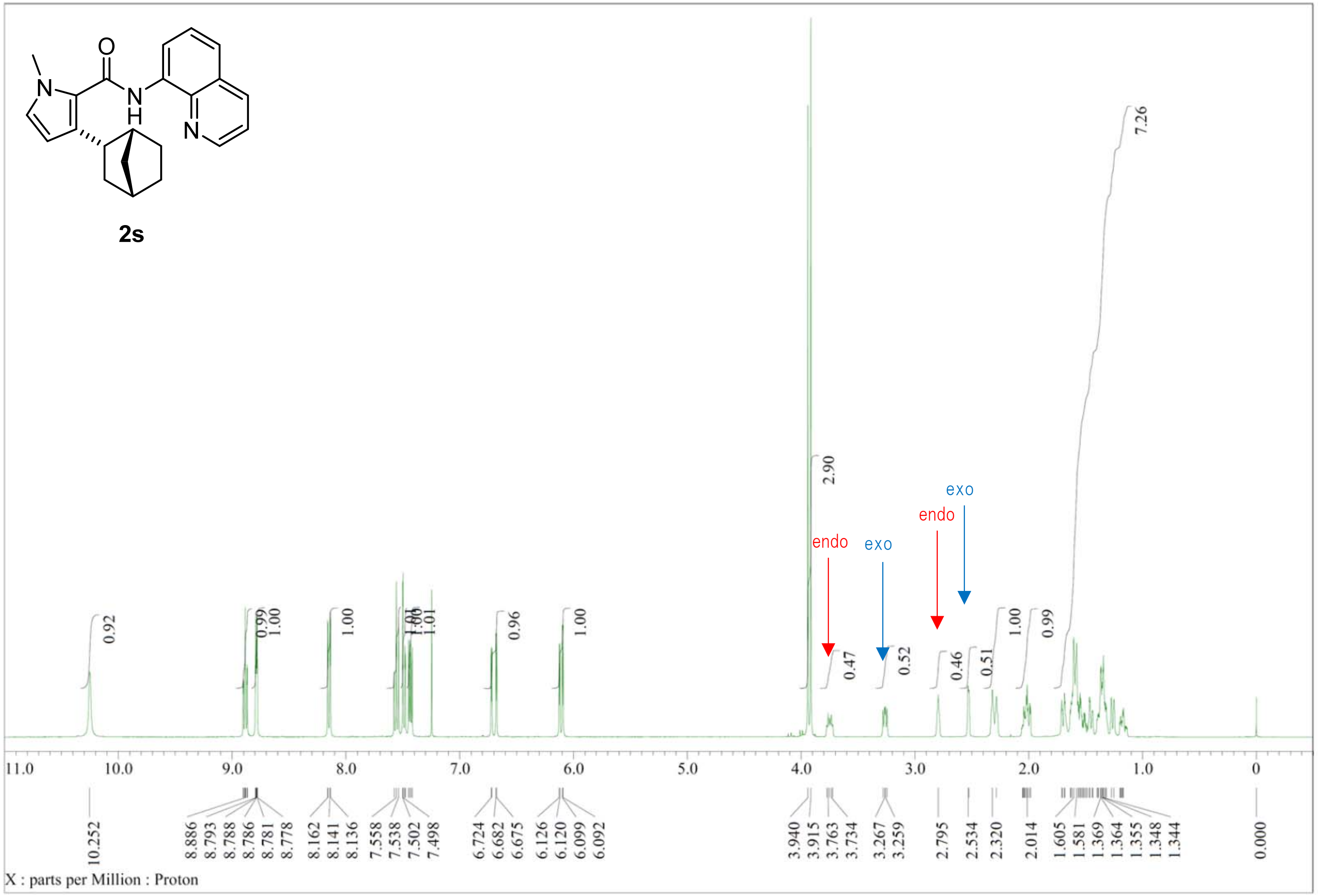

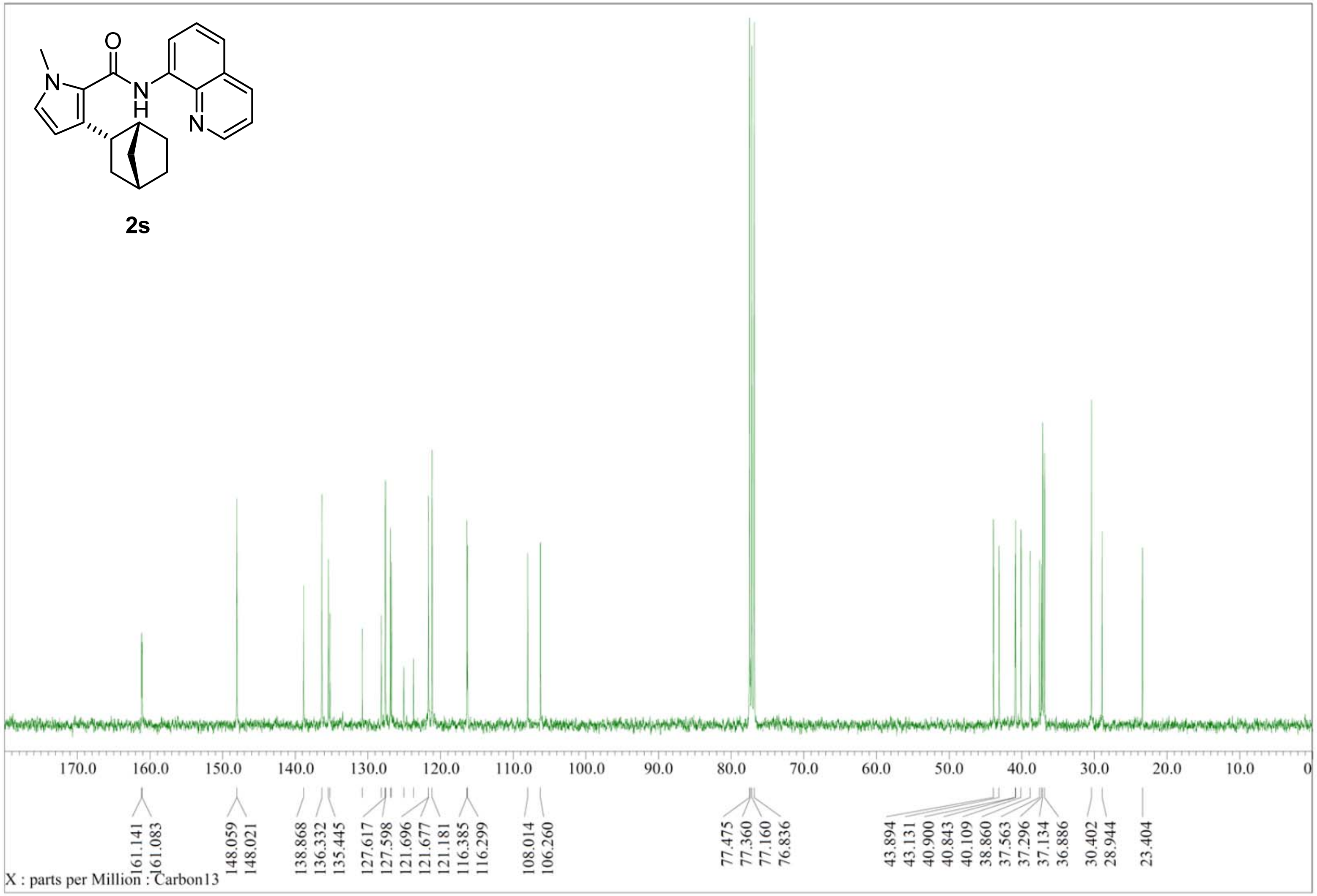
2s
170.0 160.0 150.0 140.0 130.0 120.0 110.0 100.0 90.0 80.0 70.0 60.0 50.0 40.0 30.0 20.0 10.0 0
161.141
161.083
148.059
148.021
138.868
136.332
135.445
127.617
127.598
121.696
121.677
121.181
116.385
116.299
108.014
106.260
77.475
77.360
77.160
76.836
43.894
43.131
40.900
40.843
40.109
38.860
37.563
37.296
37.134
36.886
30.402
28.944
23.404
X : parts per Million : Carbon13

Supplementary Figure 39: ^{1}H NMR, ^{13}C NMR spectra for 2t

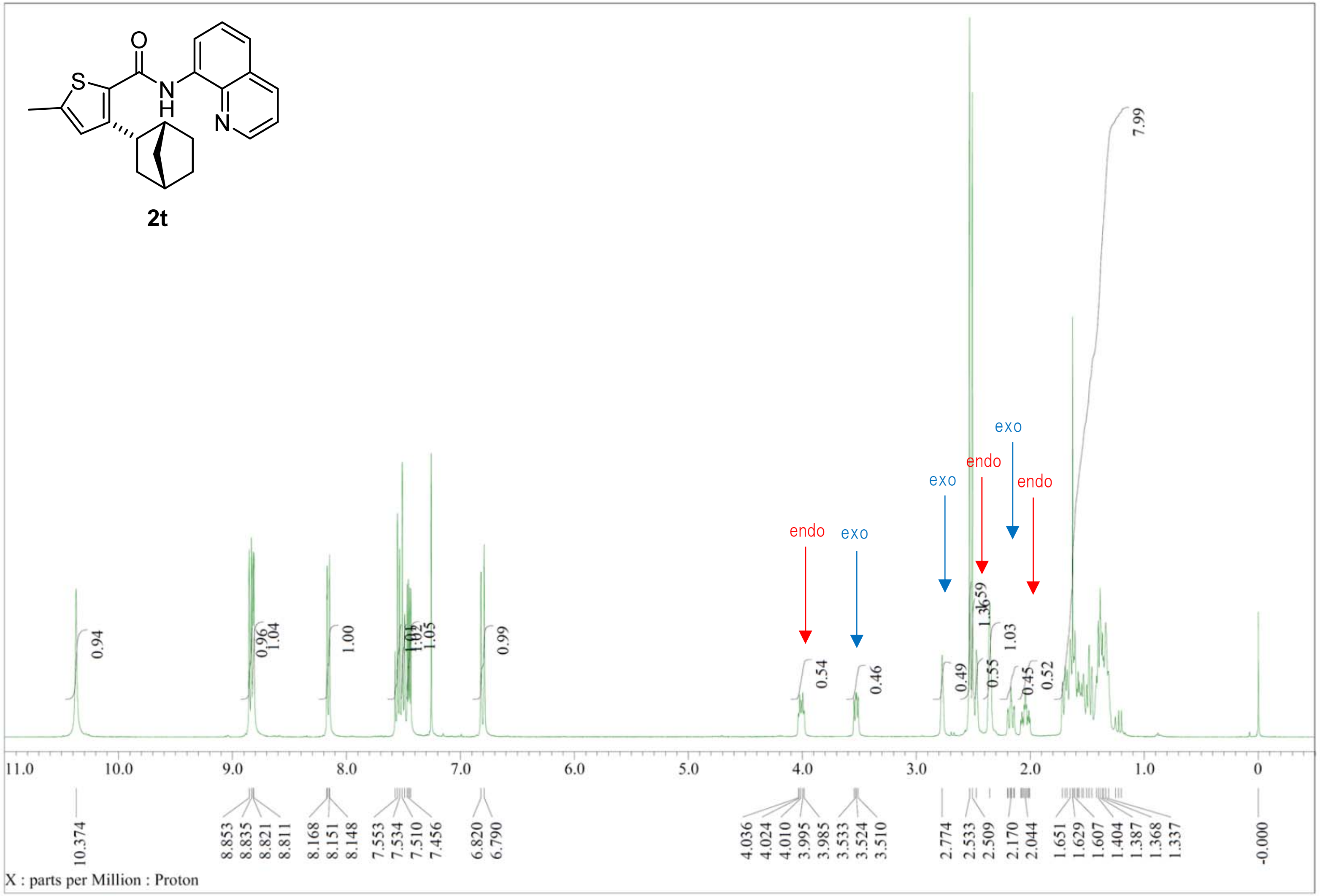

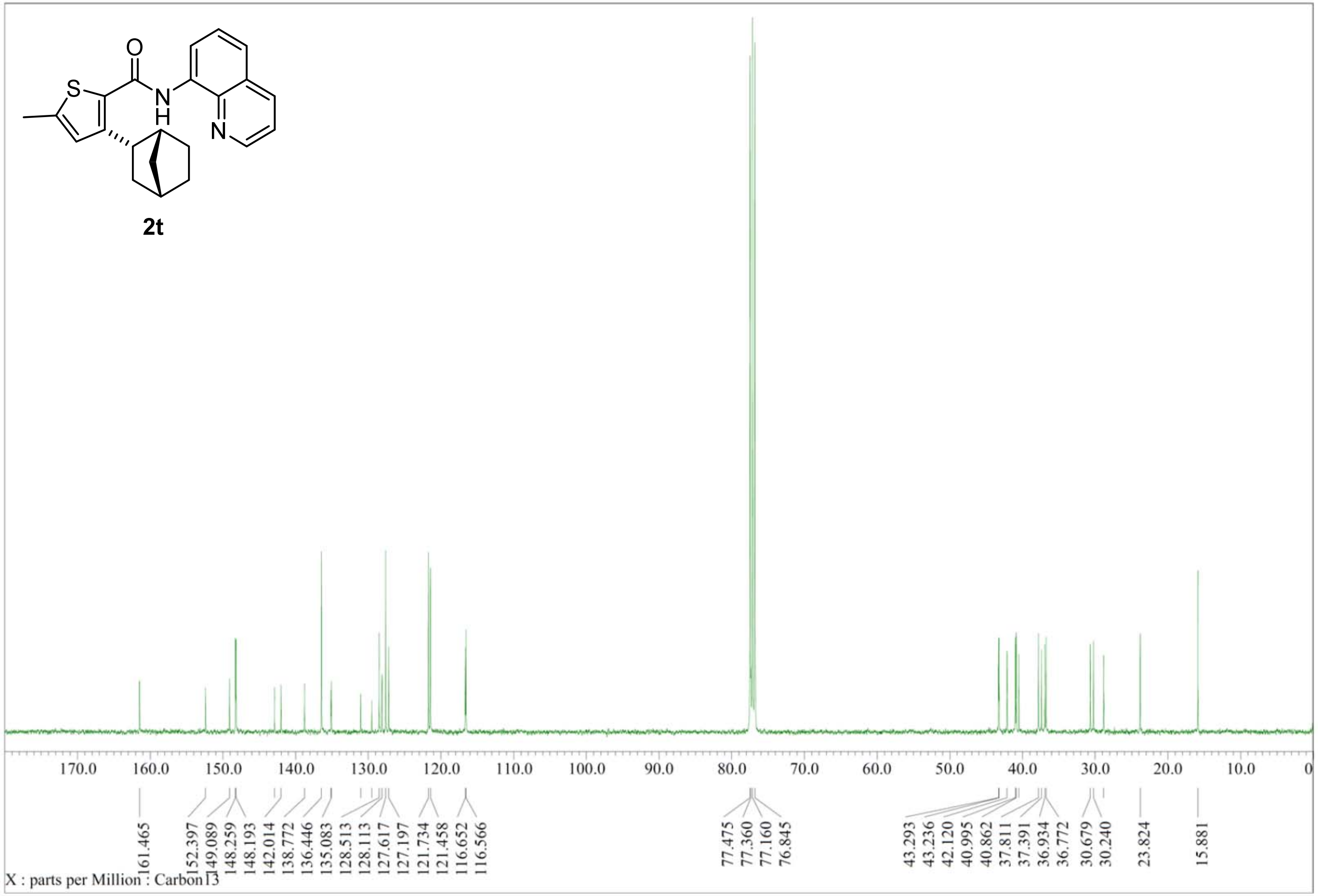
O
S
N
H
N
2t
170.0 160.0 150.0 140.0 130.0 120.0 110.0 100.0 90.0 80.0 70.0 60.0 50.0 40.0 30.0 20.0 10.0 0
161.465
152.397
149.089
148.259
148.193
142.014
138.772
136.446
135.083
128.513
128.113
127.617
127.197
121.734
121.458
116.652
116.566
77.475
77.360
77.160
76.845
43.293
43.236
42.120
40.995
40.862
37.811
37.391
36.934
36.772
30.679
30.240
23.824
15.881
X : parts per Million : Carbon13

Supplementary Figure 40: ^{1}H NMR, ^{13}C NMR spectra for 8

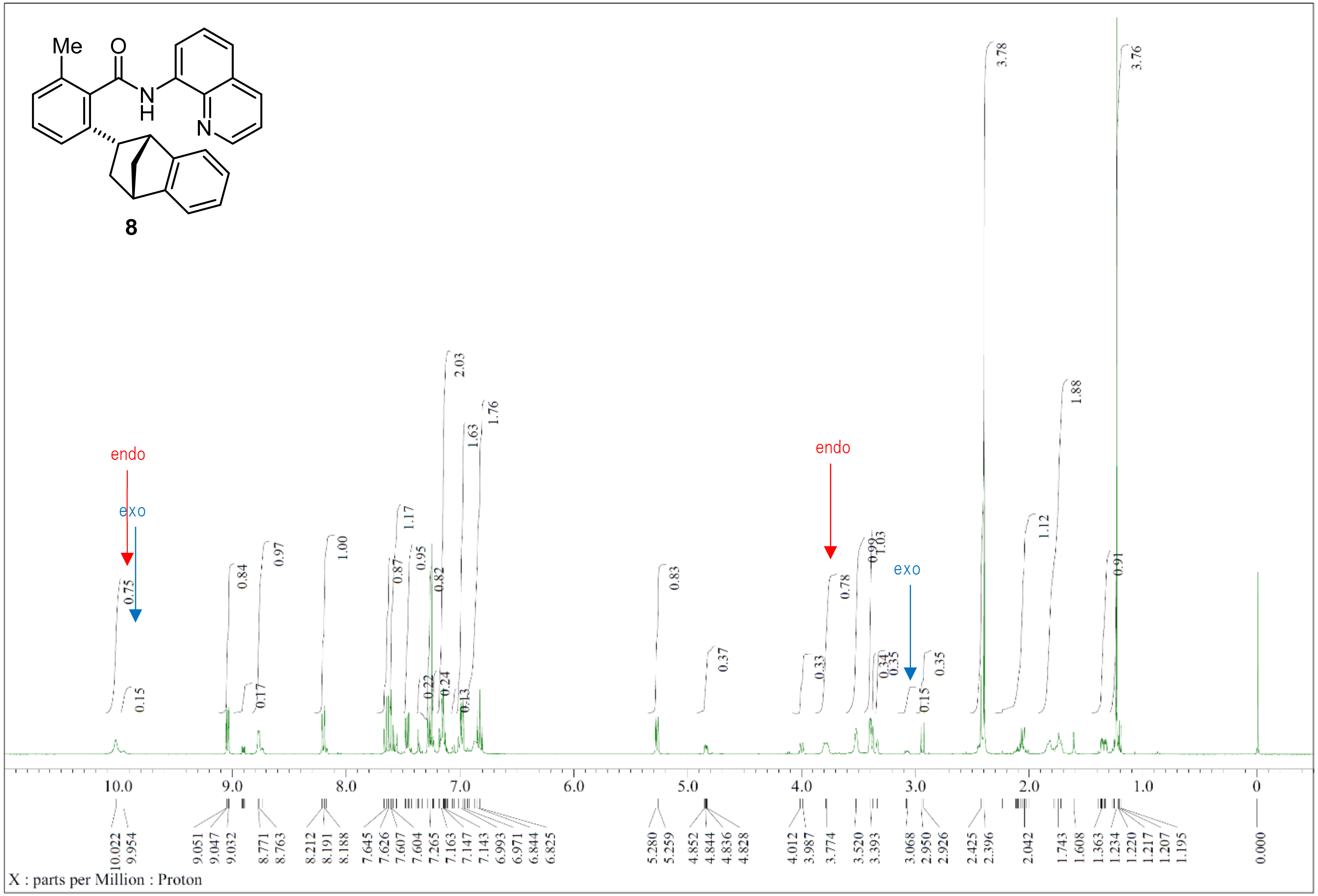

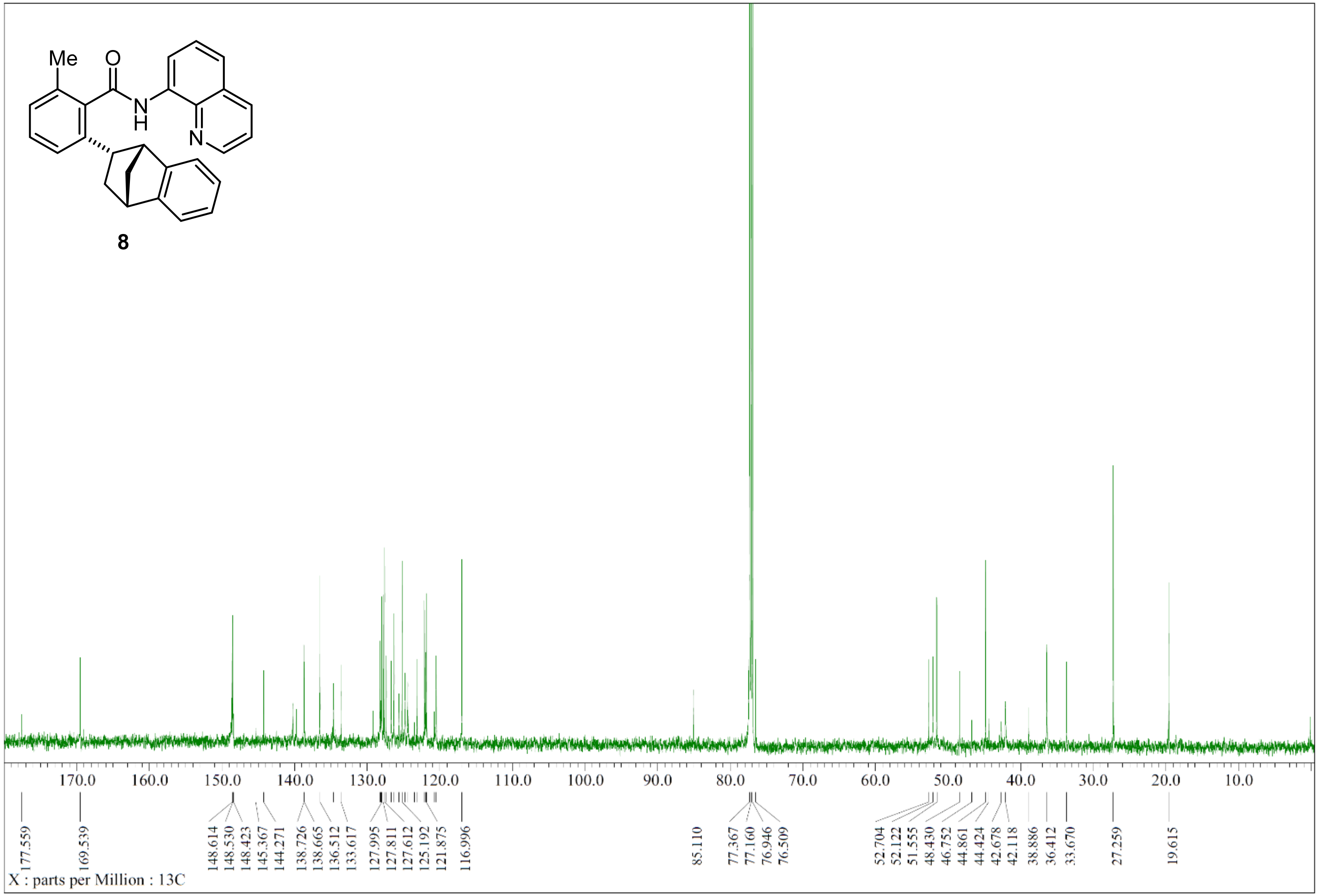
Me
O
N
H
N
8
170.0 160.0 150.0 140.0 130.0 120.0 110.0 100.0 90.0 80.0 70.0 60.0 50.0 40.0 30.0 20.0 10.0
177.559
169.539
148.614
148.530
148.423
145.367
144.271
138.726
138.665
136.512
133.617
127.995
127.811
127.612
125.192
121.875
116.996
85.110
77.367
77.160
76.946
76.509
52.704
52.122
51.555
48.430
46.752
44.861
44.424
42.678
42.118
38.886
36.412
33.670
27.259
19.615
X : parts per Million : 13C

Supplementary Figure 41: ^{1}H NMR, ^{13}C NMR spectra for 9

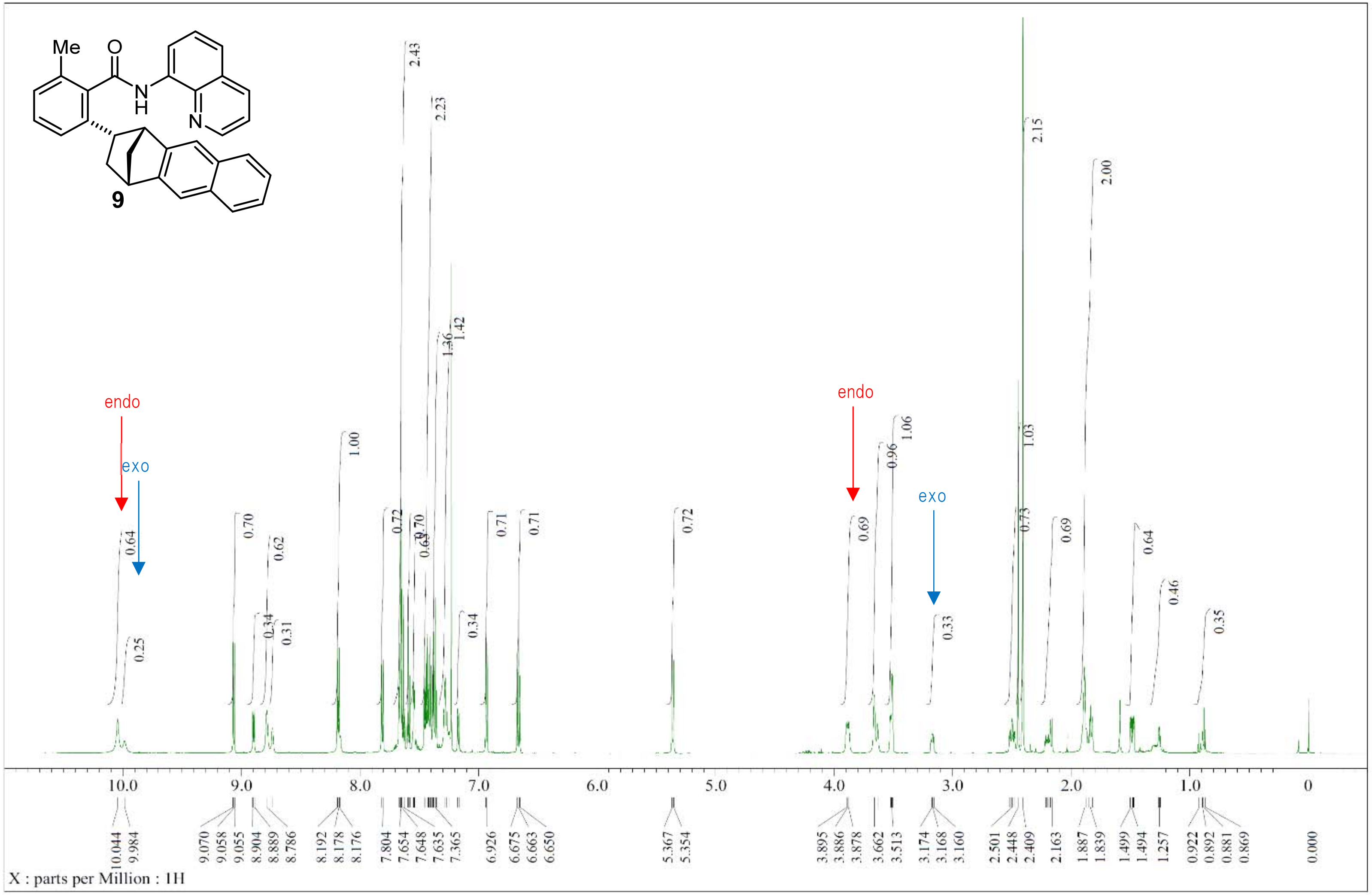

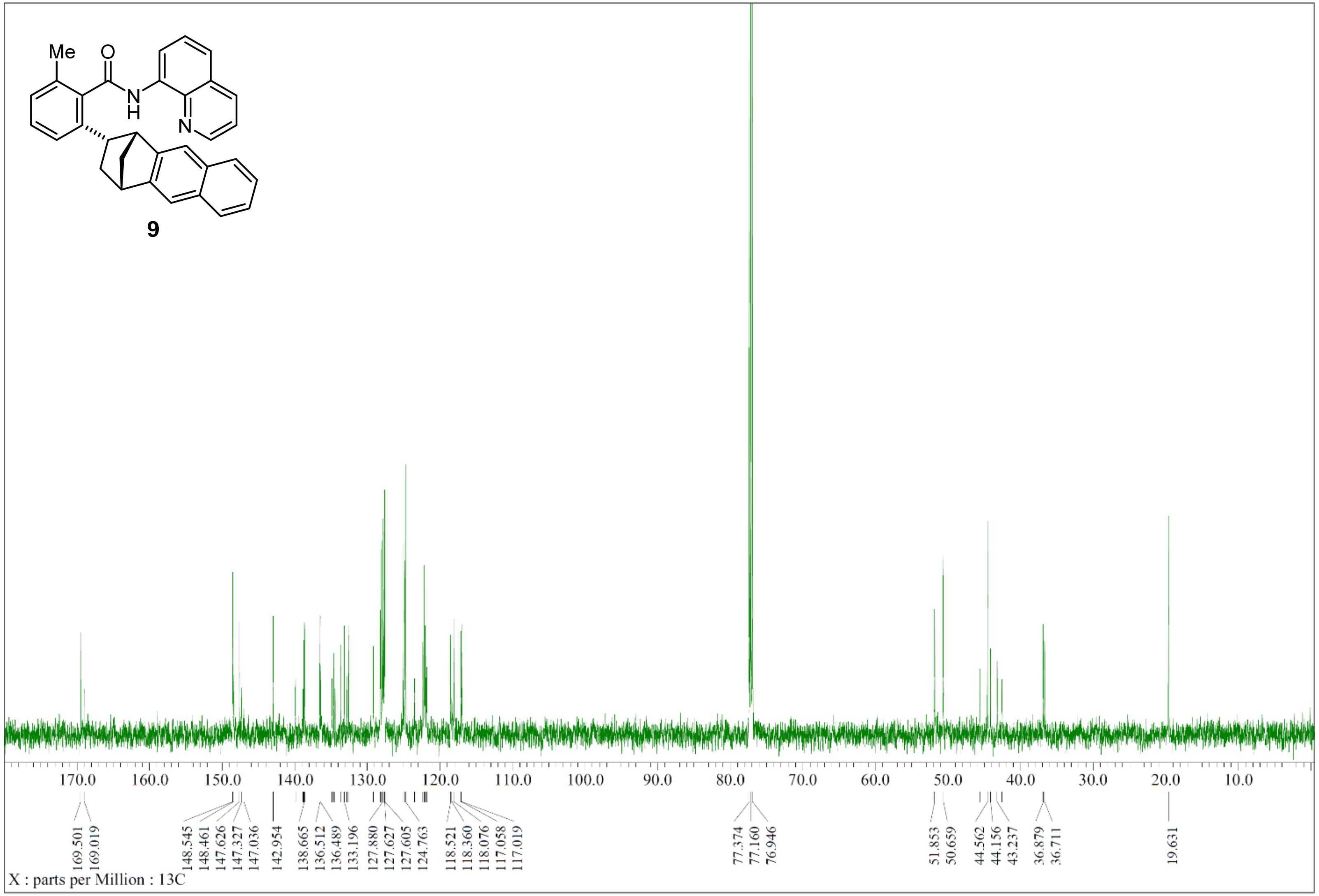
Me
O
N
H
N
9
170.0
160.0
150.0
140.0
130.0
120.0
110.0
100.0
90.0
80.0
70.0
60.0
50.0
40.0
30.0
20.0
10.0
169.501
169.019
148.545
148.461
147.626
147.327
147.036
142.954
138.665
136.512
136.489
133.196
127.880
127.627
127.605
124.763
118.521
118.360
118.076
117.058
117.019
77.374
77.160
76.946
51.853
50.659
44.562
44.156
43.237
36.879
36.711
19.631
X : parts per Million : 13C

Supplementary Figure 42: ^{1}H NMR, ^{13}C NMR spectra for 10a

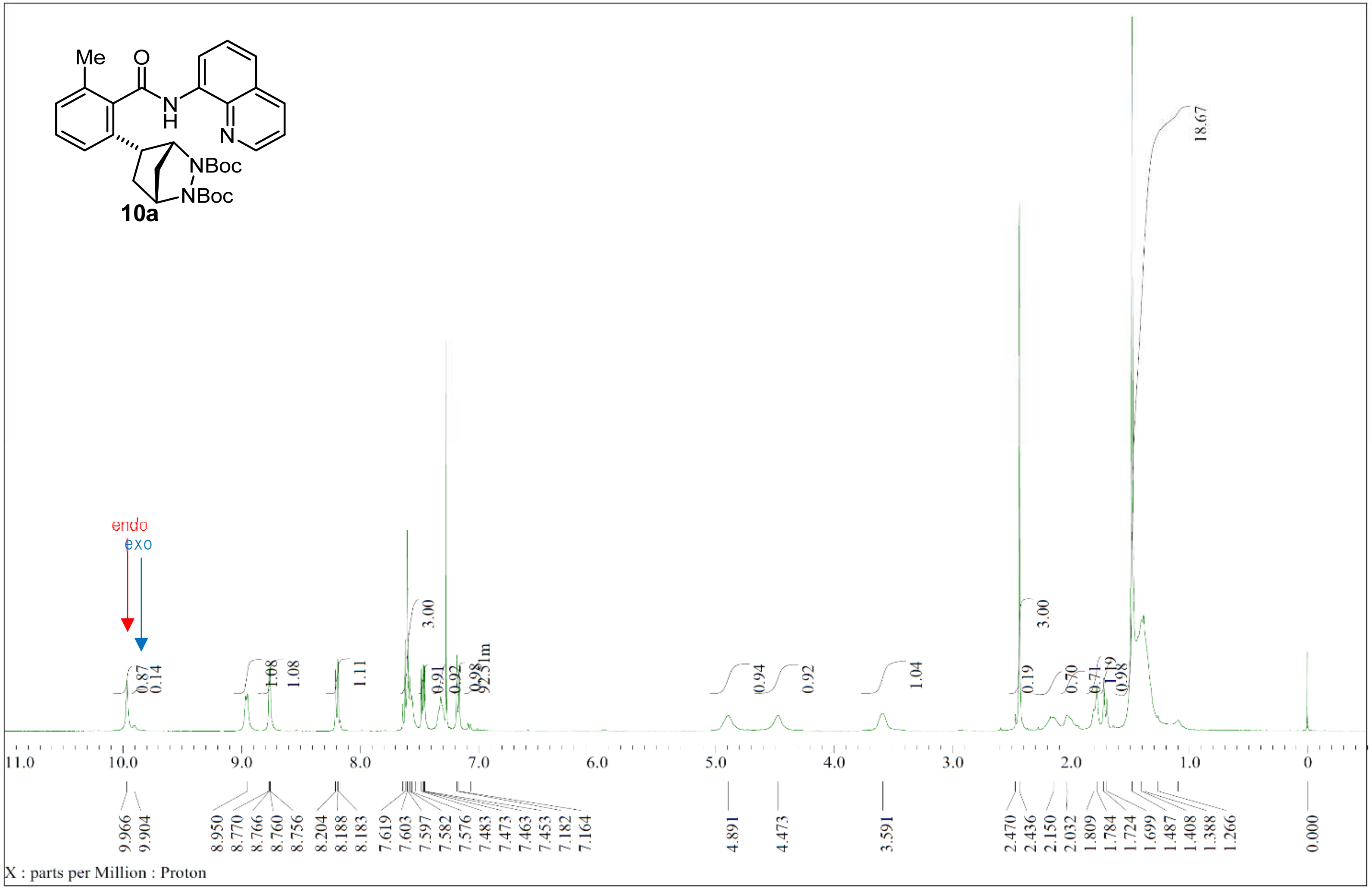

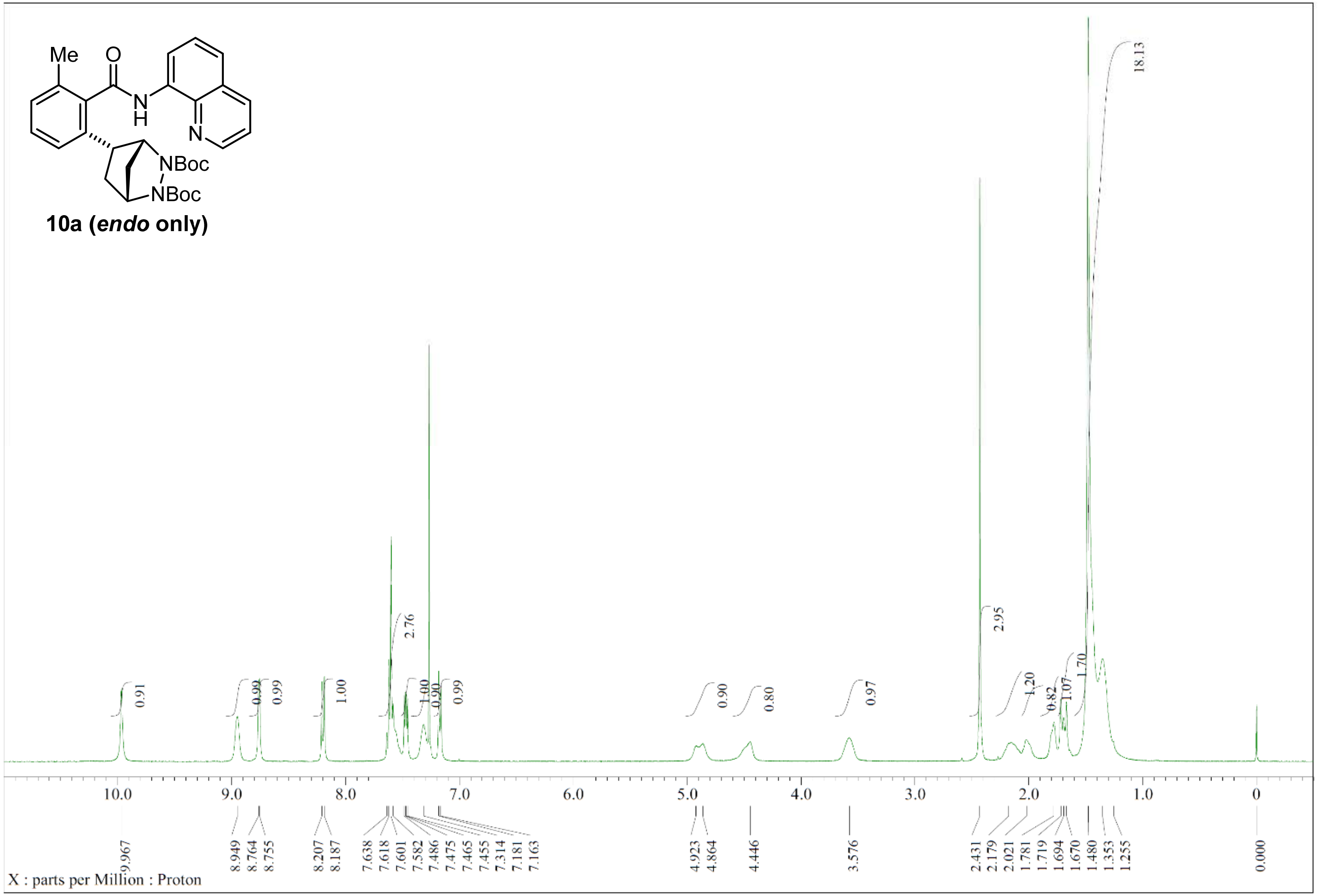
Me
O
N
H
N
NBoc
NBoc
10a (endo only)
X : parts per Million : Proton

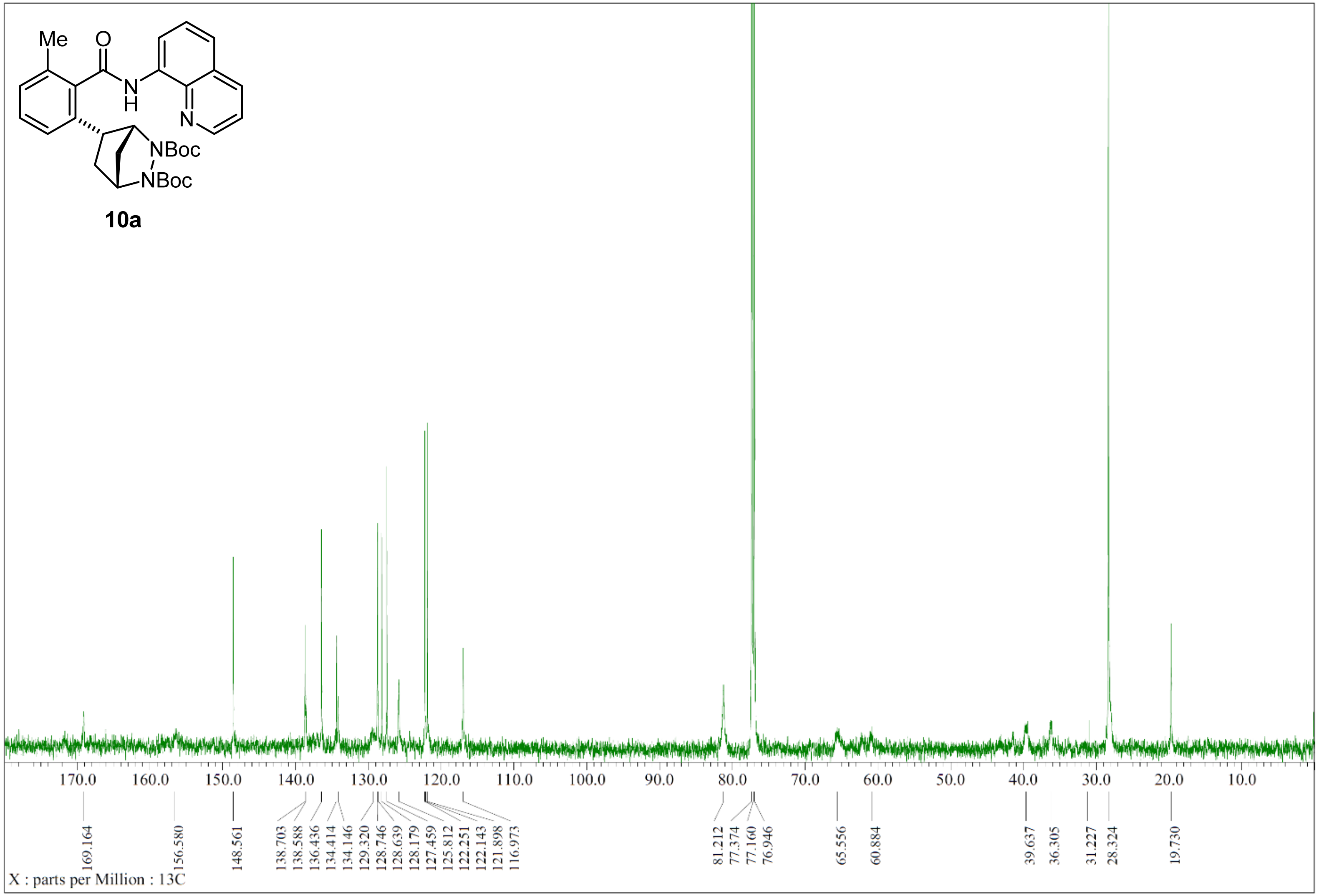

Me
O
N
H
N
NBoc
NBoc
10a
170.0 160.0 150.0 140.0 130.0 120.0 110.0 100.0 90.0 80.0 70.0 60.0 50.0 40.0 30.0 20.0 10.0
169.164
156.580
148.561
138.703
138.588
136.436
134.414
134.146
129.320
128.746
128.639
128.179
127.459
125.812
122.251
122.143
121.898
116.973
81.212
77.374
77.160
76.946
65.556
60.884
39.637
36.305
31.227
28.324
19.730
X : parts per Million : 13C

Supplementary Figure 43: ^{1}H NMR, ^{13}C NMR spectra for 10b

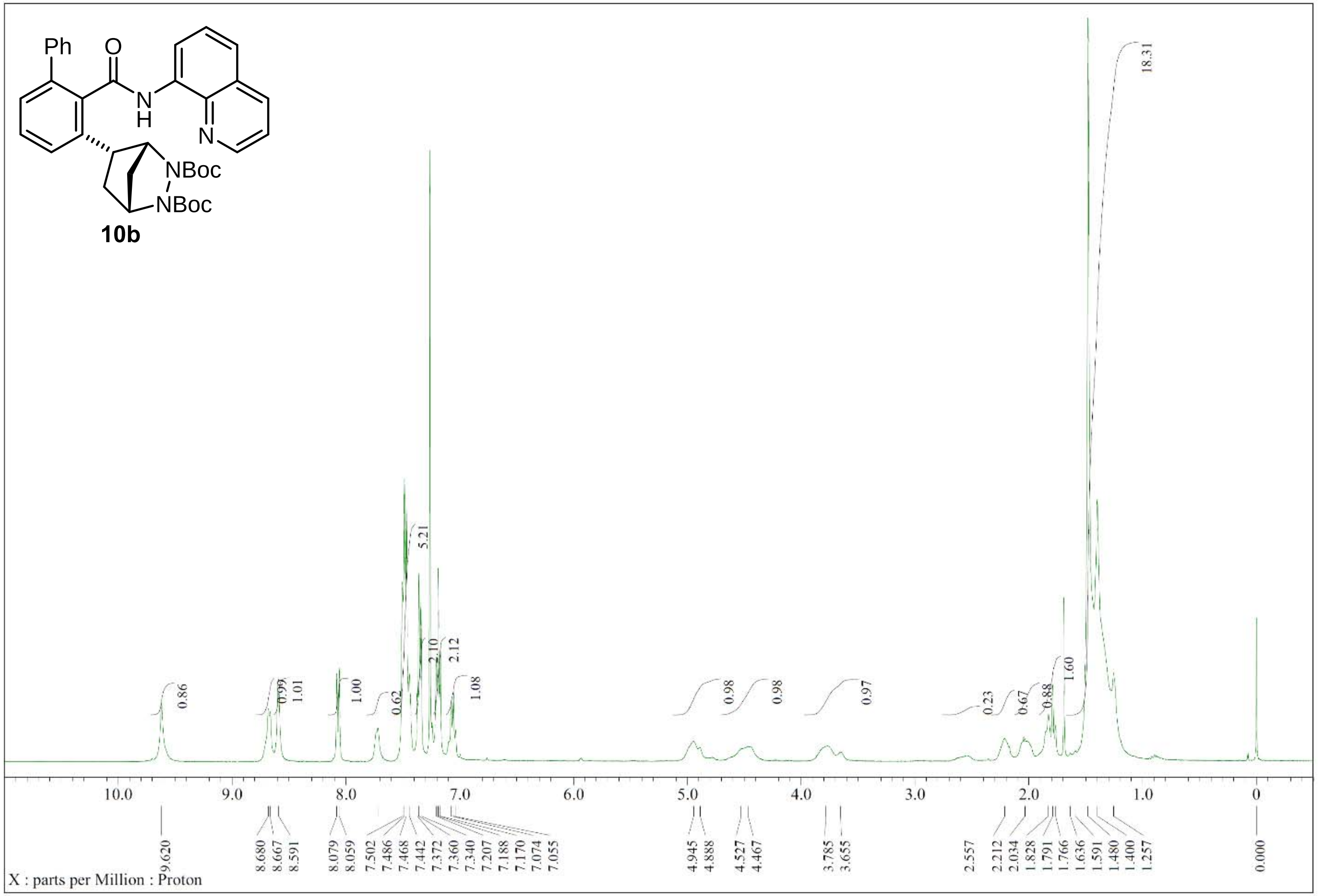

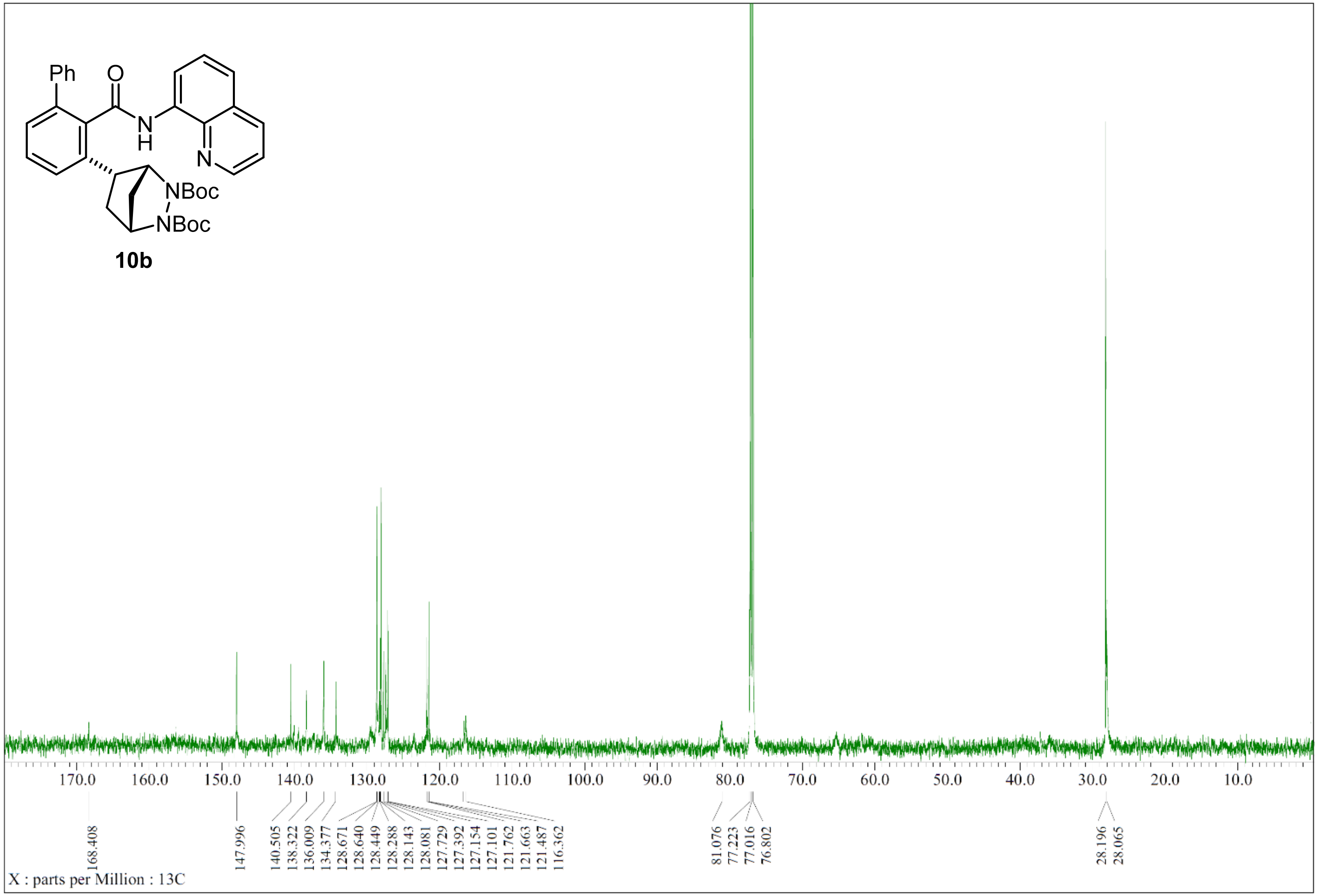
Ph
O
N
H
N
NBoc
NBoc
10b
170.0 160.0 150.0 140.0 130.0 120.0 110.0 100.0 90.0 80.0 70.0 60.0 50.0 40.0 30.0 20.0 10.0
168.408
147.996
140.505
138.322
136.009
134.377
128.671
128.640
128.449
128.288
128.143
128.081
127.729
127.392
127.154
127.101
121.762
121.663
121.487
116.362
81.076
77.223
77.016
76.802
28.196
28.065
X : parts per Million : 13C

Supplementary Figure 44: ^{1}H NMR, ^{13}C NMR spectra for 10c

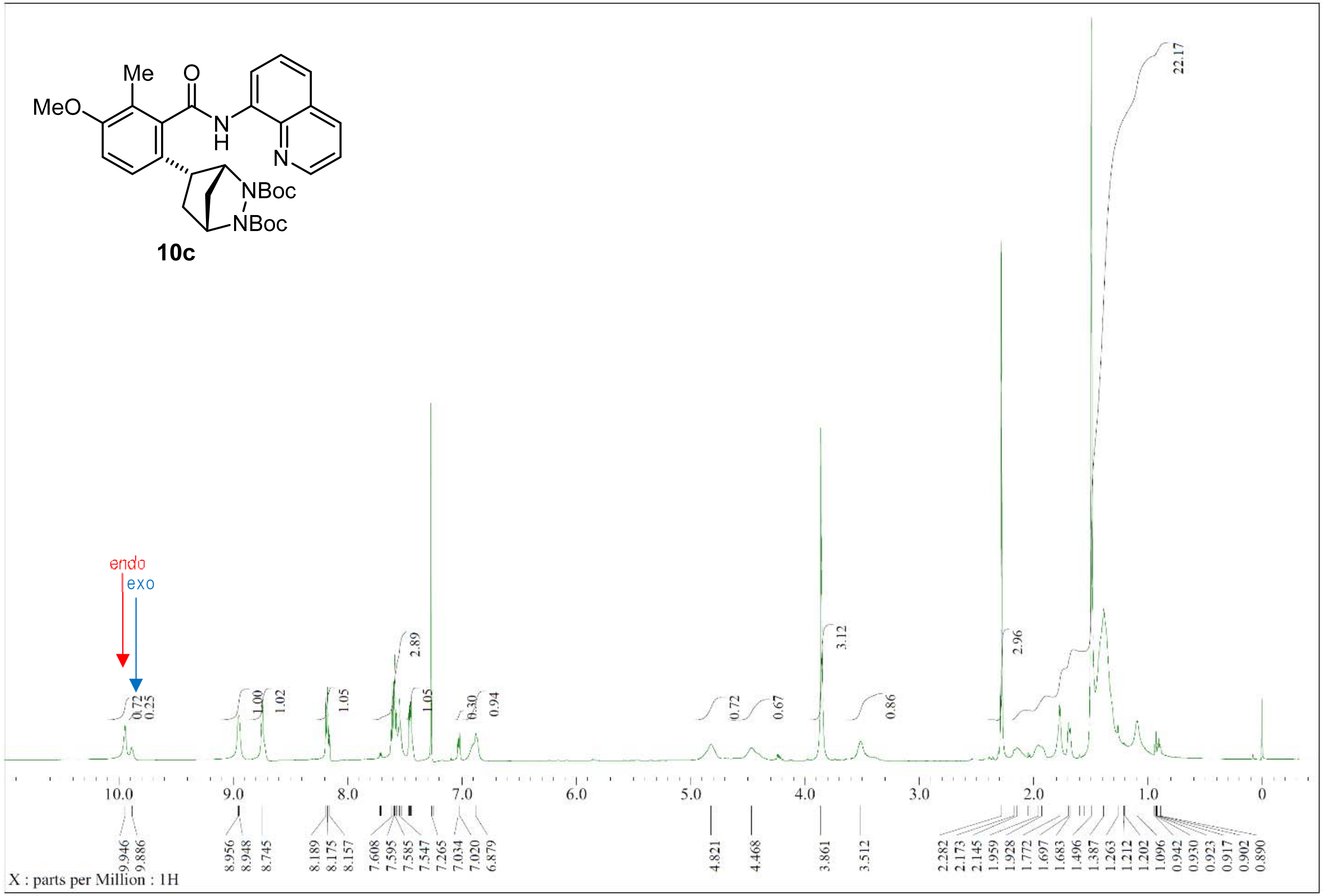

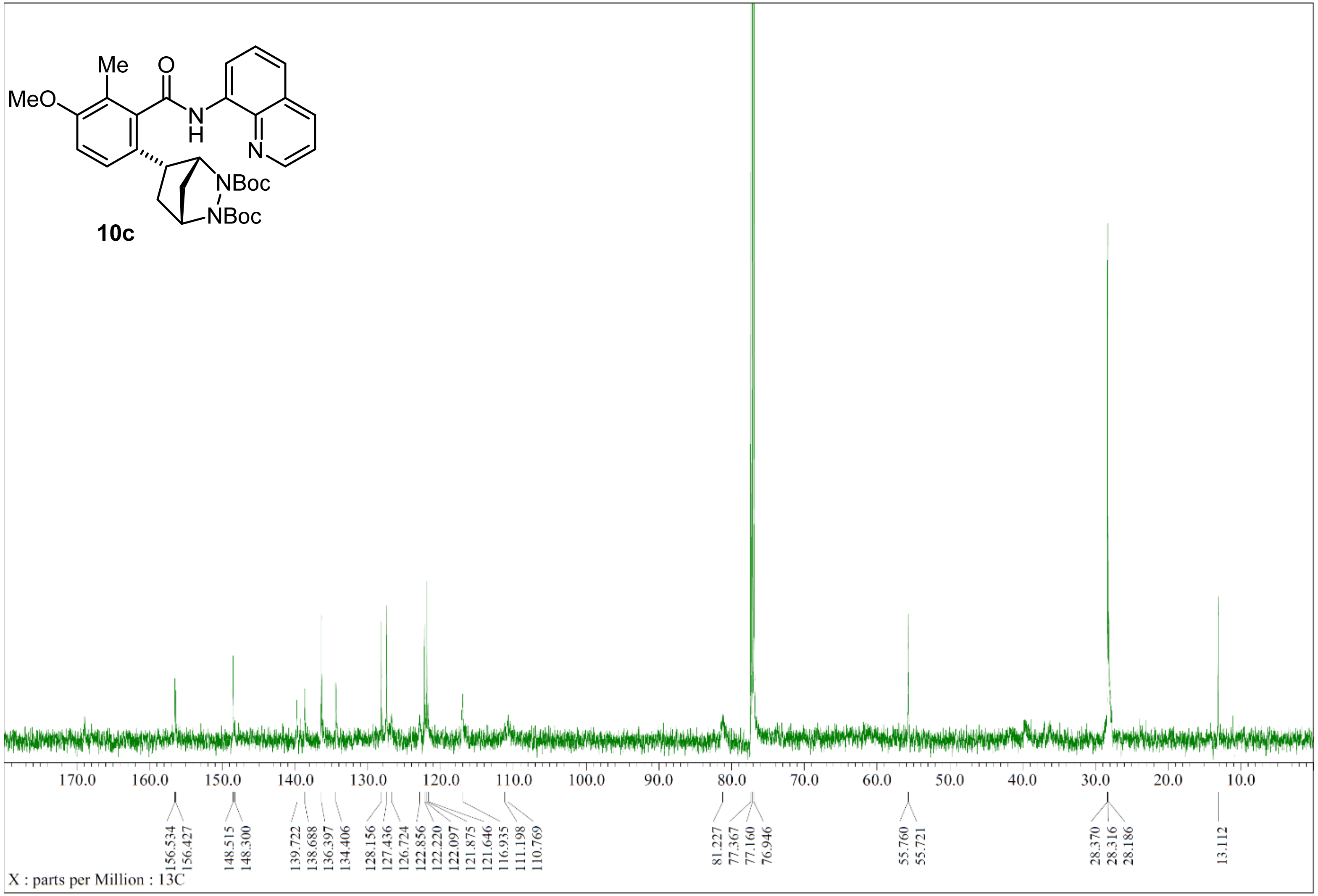

MeO
Me
O
N
H
N
NBoc
NBoc
10c
170.0 160.0 150.0 140.0 130.0 120.0 110.0 100.0 90.0 80.0 70.0 60.0 50.0 40.0 30.0 20.0 10.0
156.534
156.427
148.515
148.300
139.722
138.688
136.397
134.406
128.156
127.436
126.724
122.856
122.220
122.097
121.875
121.646
116.935
111.198
110.769
81.227
77.367
77.160
76.946
55.760
55.721
28.370
28.316
28.186
13.112
X : parts per Million : 13C

Supplementary Figure 45: ^{1}H NMR, ^{13}C NMR spectra for 10d

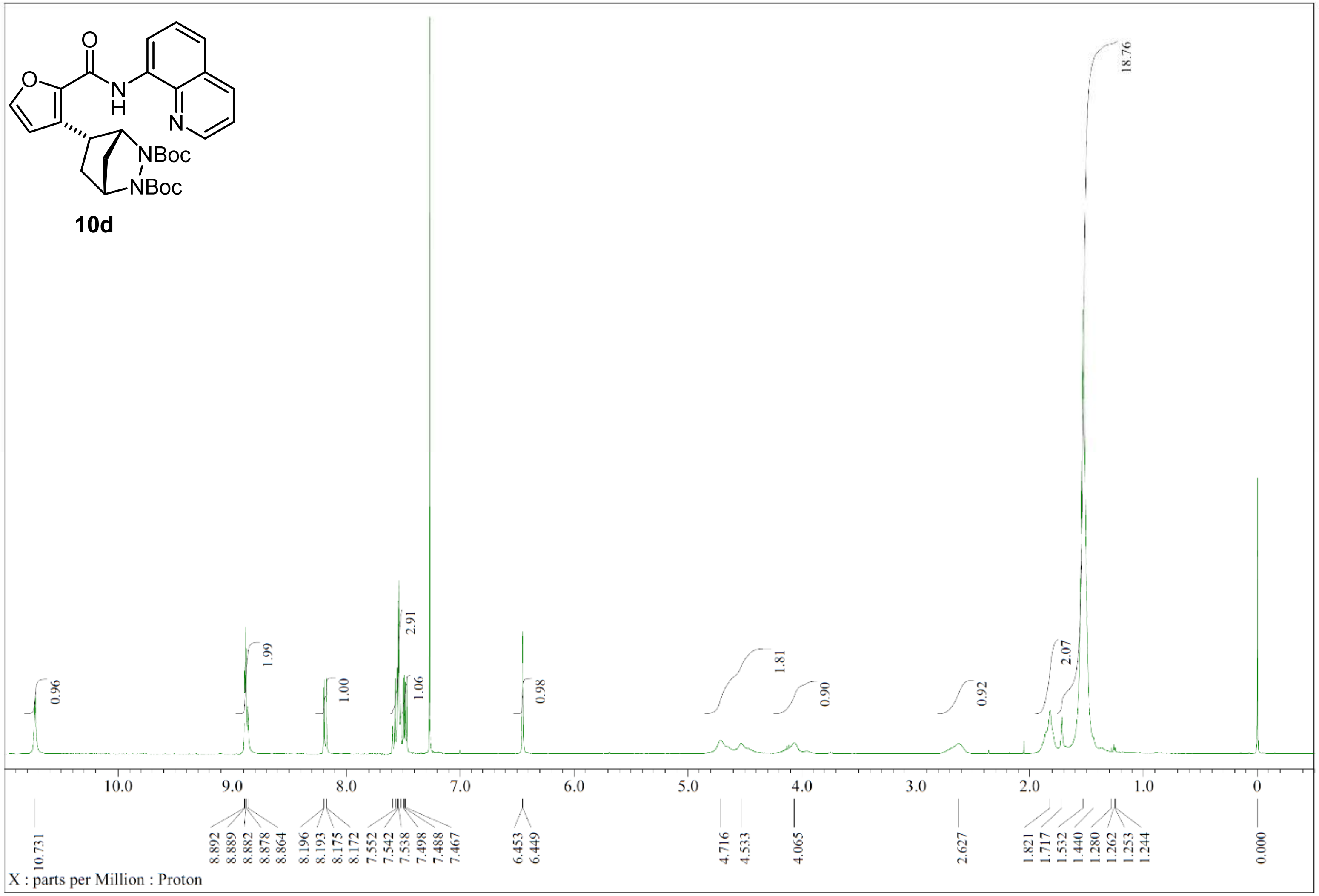

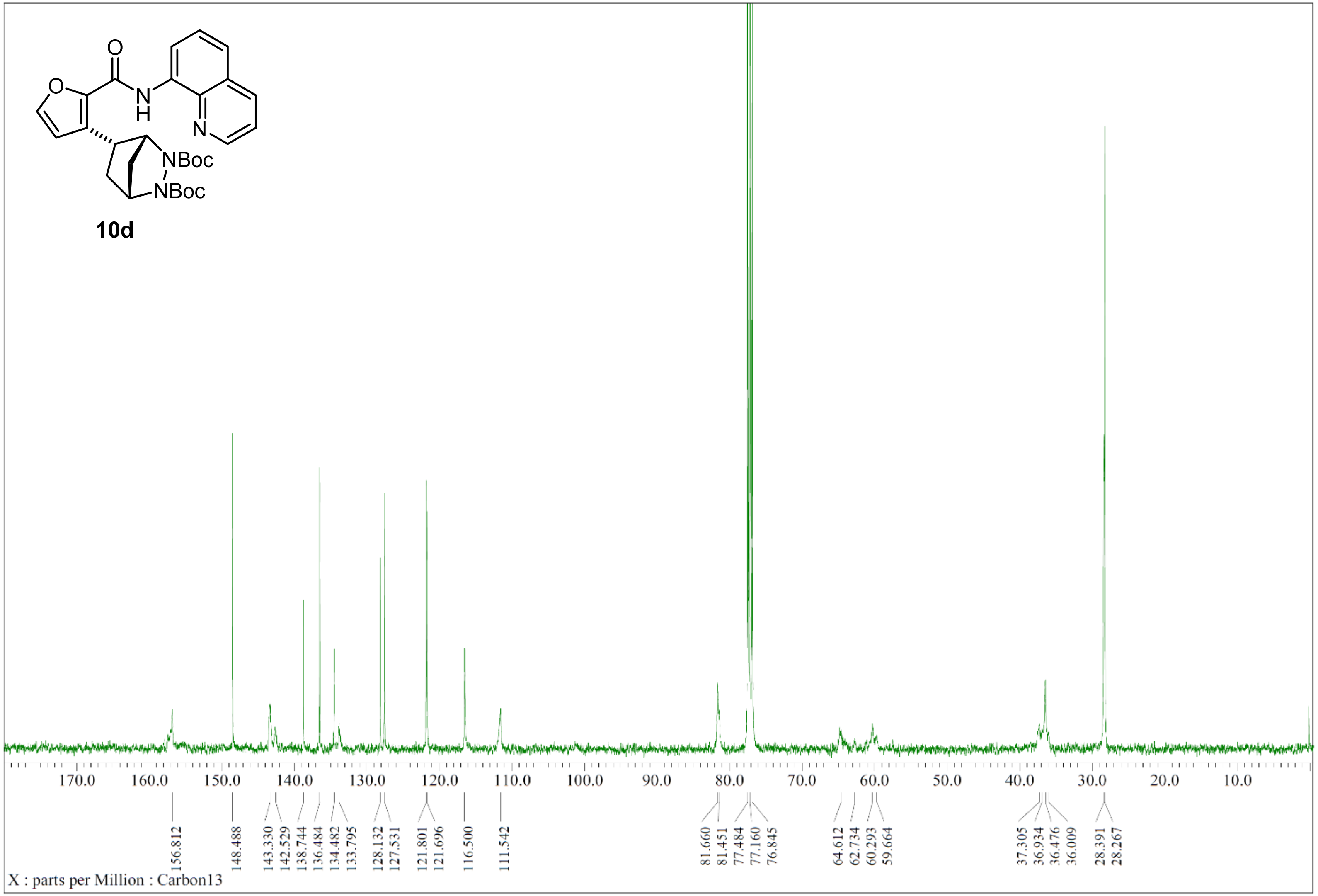
O
O
N
H
N
NBoc
NBoc
10d
170.0 160.0 150.0 140.0 130.0 120.0 110.0 100.0 90.0 80.0 70.0 60.0 50.0 40.0 30.0 20.0 10.0
156.812
148.488
143.330
142.529
138.744
136.484
134.482
133.795
128.132
127.531
121.801
121.696
116.500
111.542
81.660
81.451
77.484
77.160
76.845
64.612
62.734
60.293
59.664
37.305
36.934
36.476
36.009
28.391
28.267
X : parts per Million : Carbon13

Supplementary Figure 46: ^{1}H NMR, ^{13}C NMR spectra for 1l

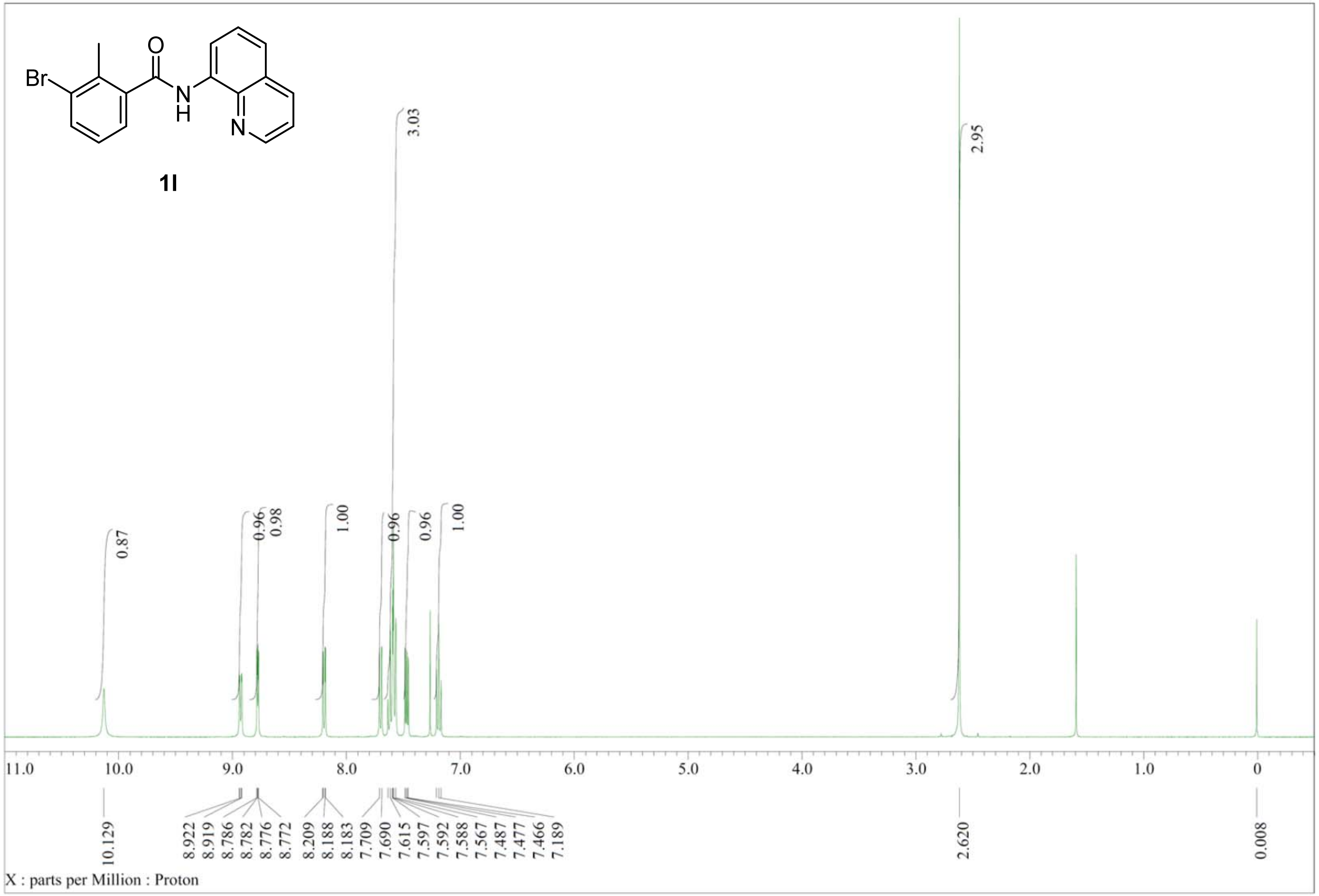

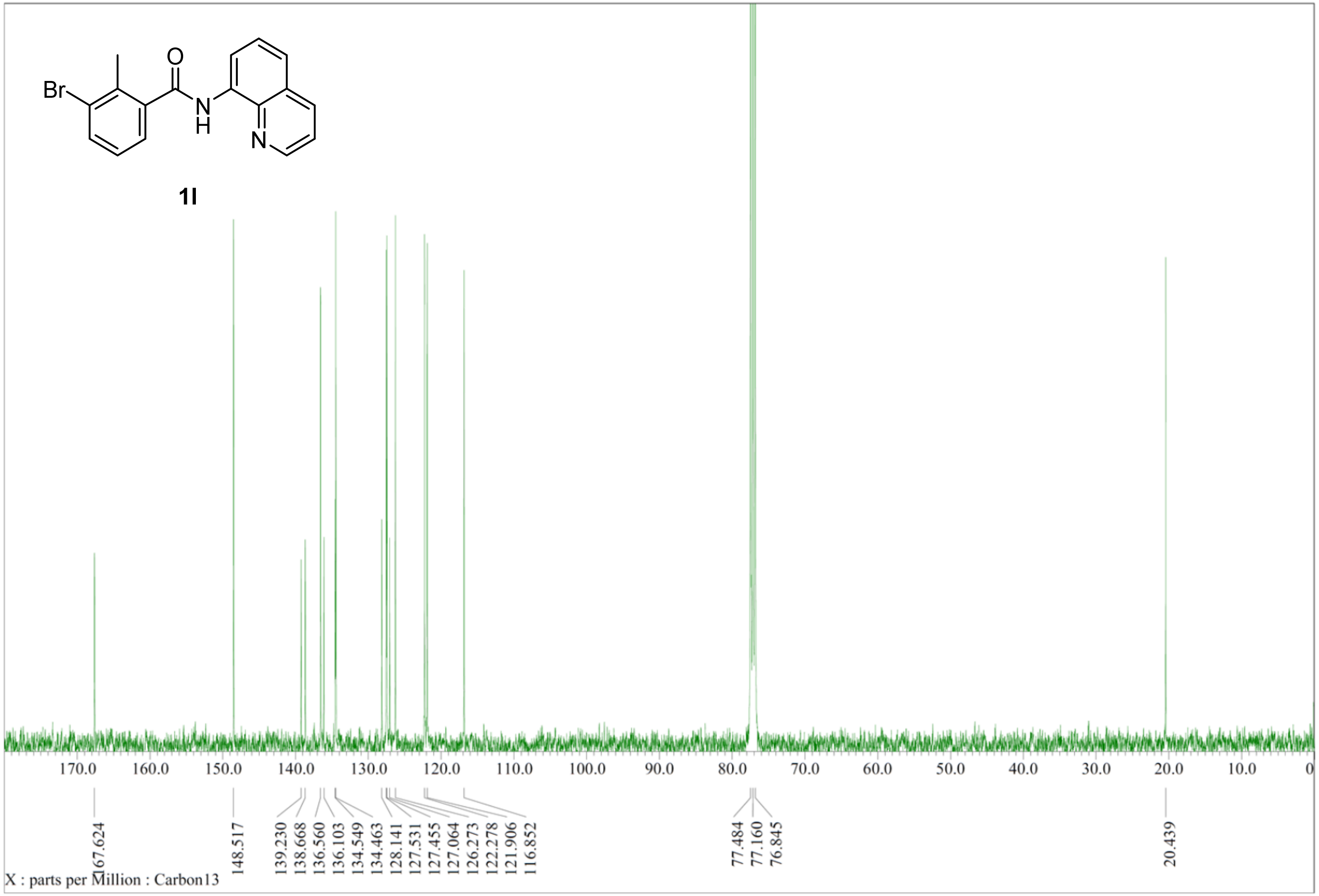
Br
O
N
H
N
1l
170.0
160.0
150.0
140.0
130.0
120.0
110.0
100.0
90.0
80.0
70.0
60.0
50.0
40.0
30.0
20.0
10.0
0
167.624
148.517
139.230
138.668
136.560
136.103
134.549
134.463
128.141
127.531
127.455
127.064
126.273
122.278
121.906
116.852
77.484
77.160
76.845
20.439
X : parts per Million : Carbon13

Supplementary Figure 47: ^{1}H NMR, ^{13}C NMR spectra for 1m

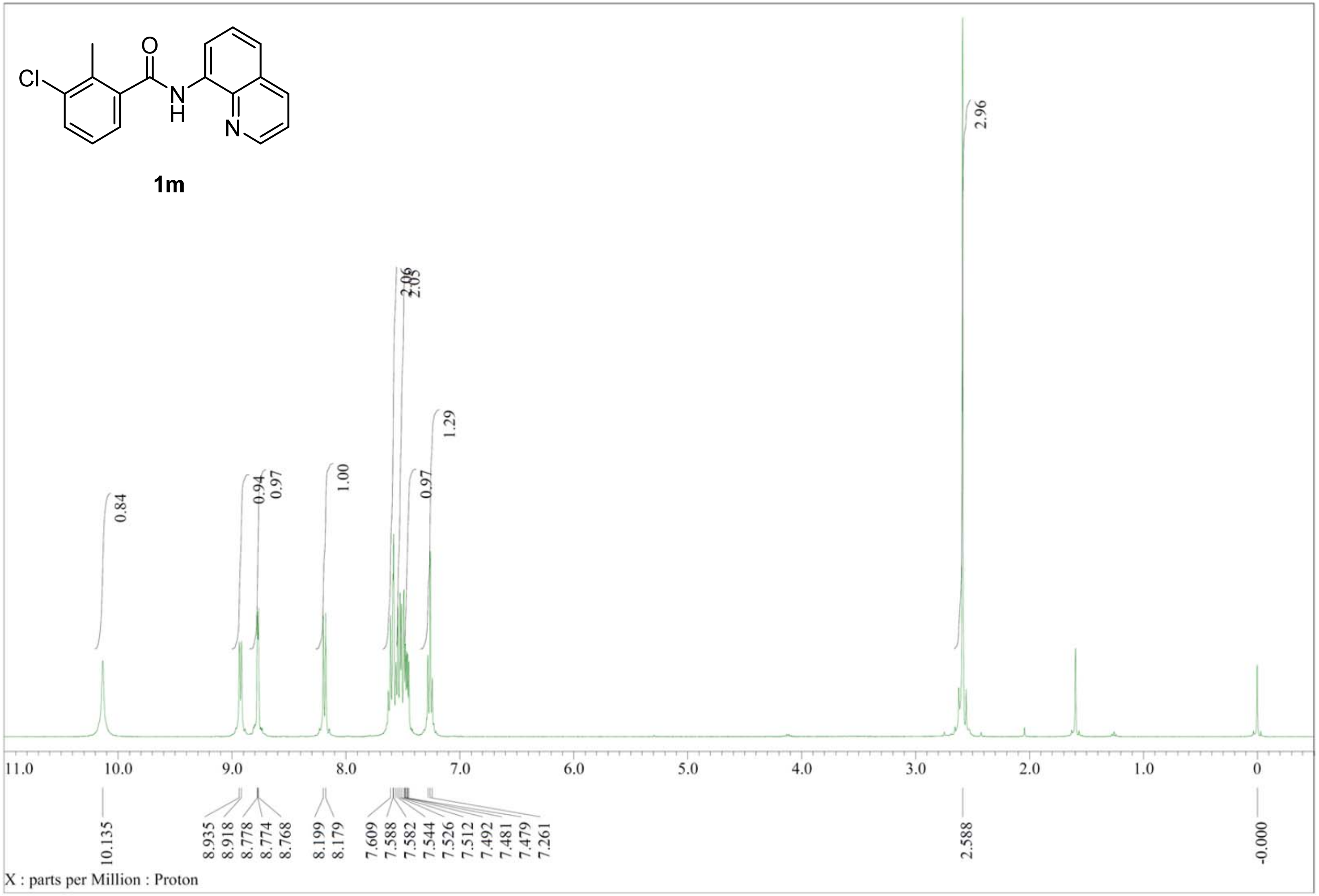

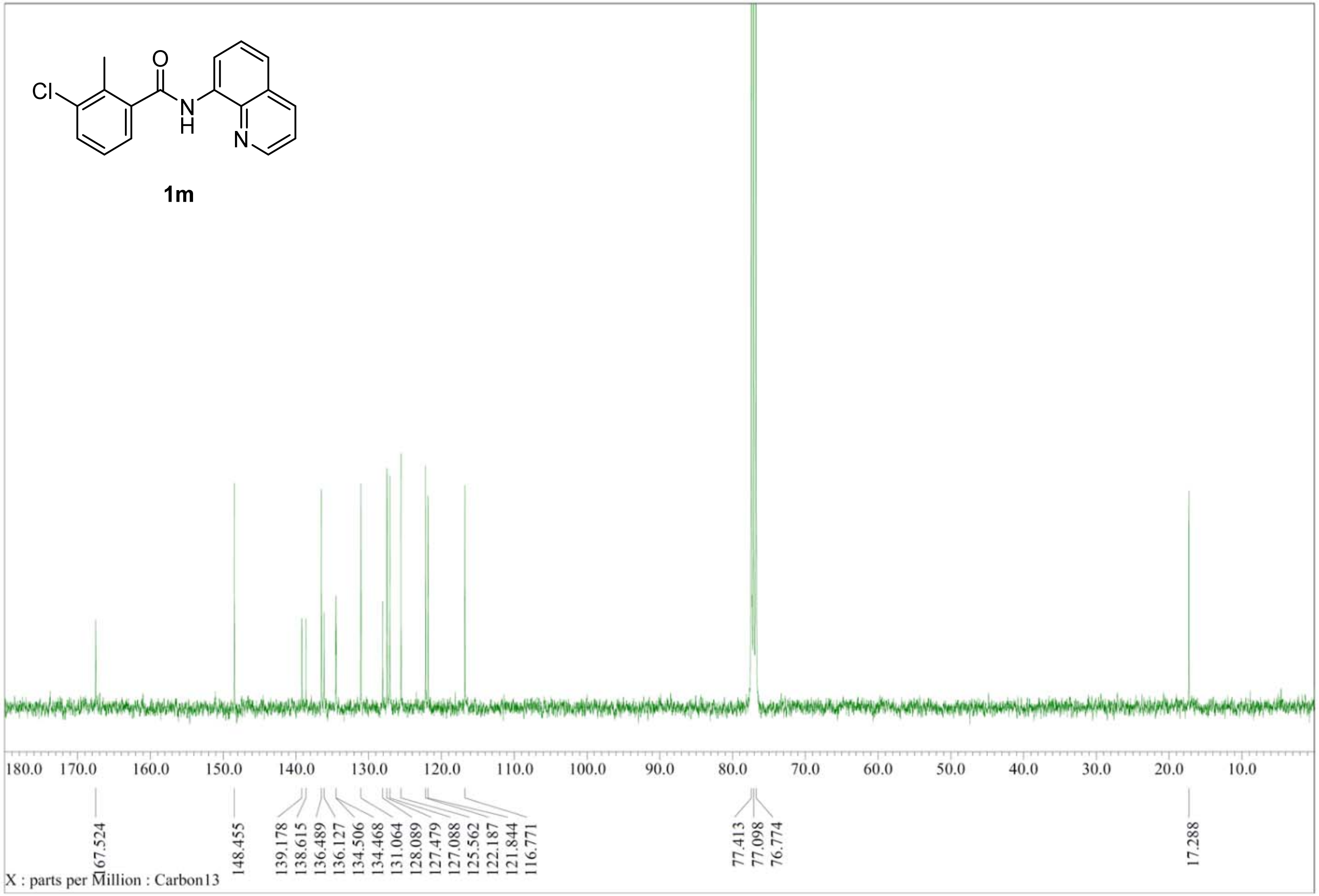
Cl
O
N
H
N
1m
180.0 170.0 160.0 150.0 140.0 130.0 120.0 110.0 100.0 90.0 80.0 70.0 60.0 50.0 40.0 30.0 20.0 10.0
167.524
148.455
139.178
138.615
136.489
136.127
134.506
134.468
131.064
128.089
127.479
127.088
125.562
122.187
121.844
116.771
77.413
77.098
76.774
17.288
X : parts per Million : Carbon13

Supplementary Figure 48: ^{1}H NMR, ^{13}C NMR spectra for 1s

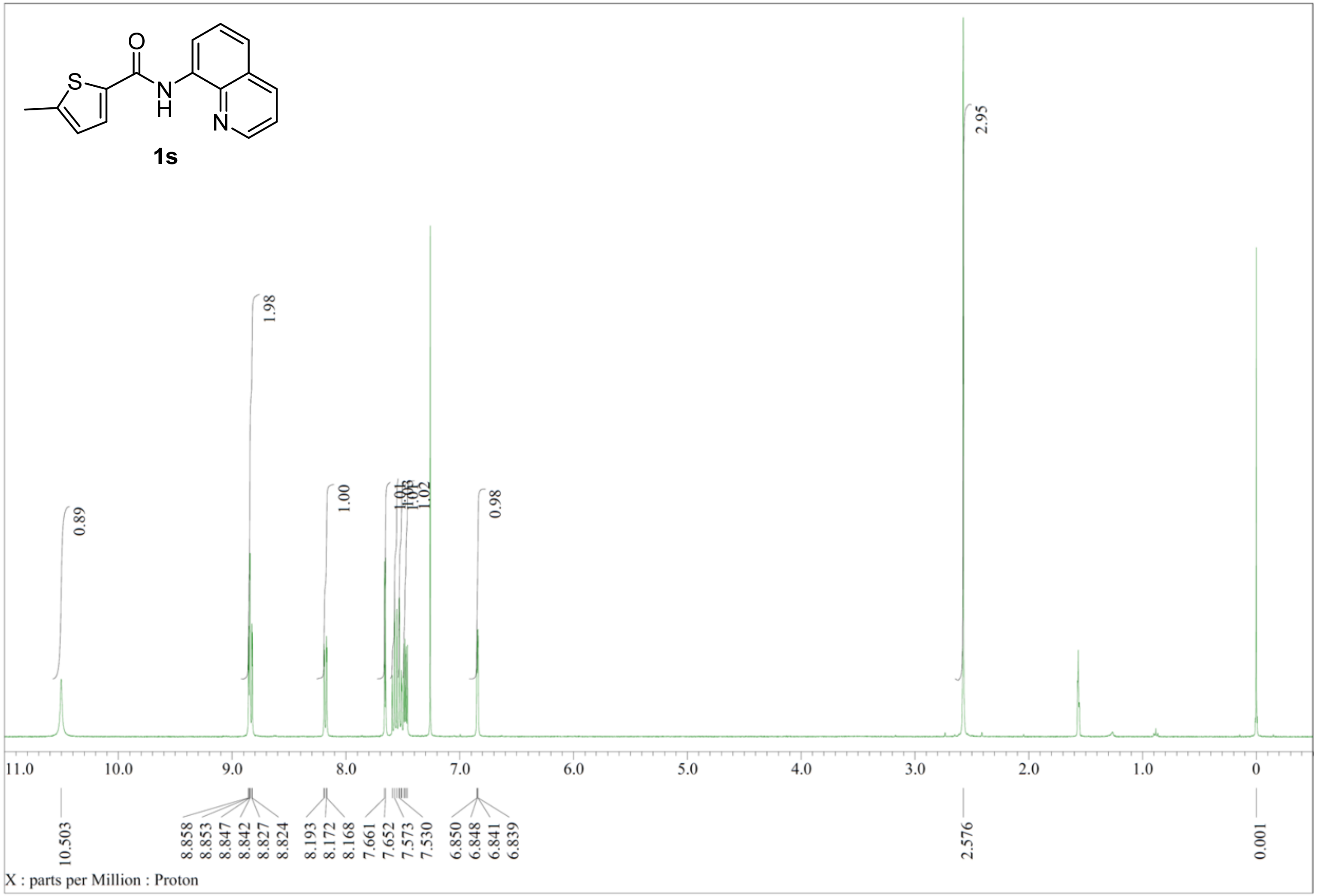

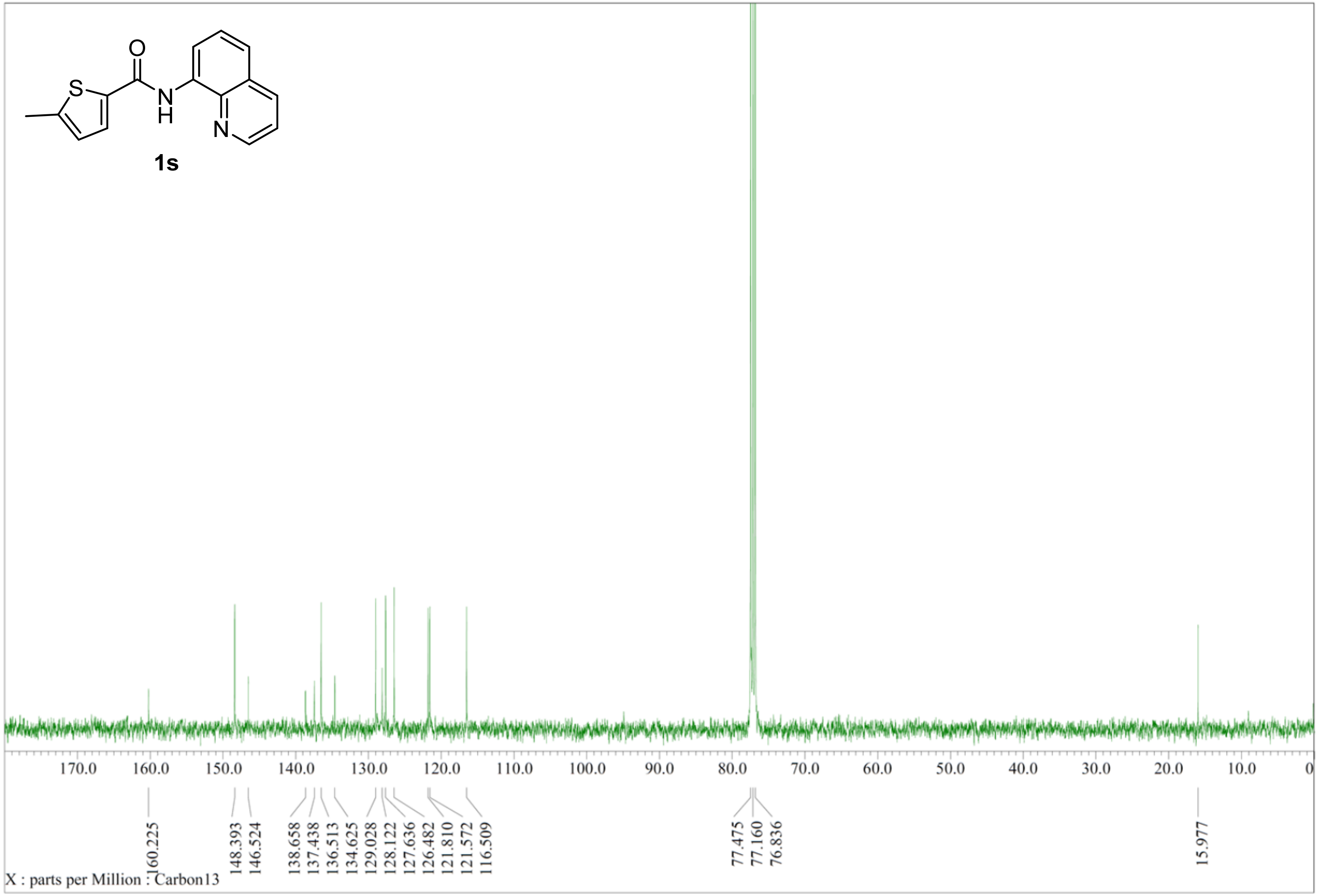
O
S
N
H
N
1s
170.0
160.0
150.0
140.0
130.0
120.0
110.0
100.0
90.0
80.0
70.0
60.0
50.0
40.0
30.0
20.0
10.0
0
160.225
148.393
146.524
138.658
137.438
136.513
134.625
129.028
128.122
127.636
126.482
121.810
121.572
116.509
77.475
77.160
76.836
15.977
X : parts per Million : Carbon13

Supplementary Figure 49: ^{1}H NMR spectra for 1,4-dihydro-1,4-methanonaphthalene-d_6

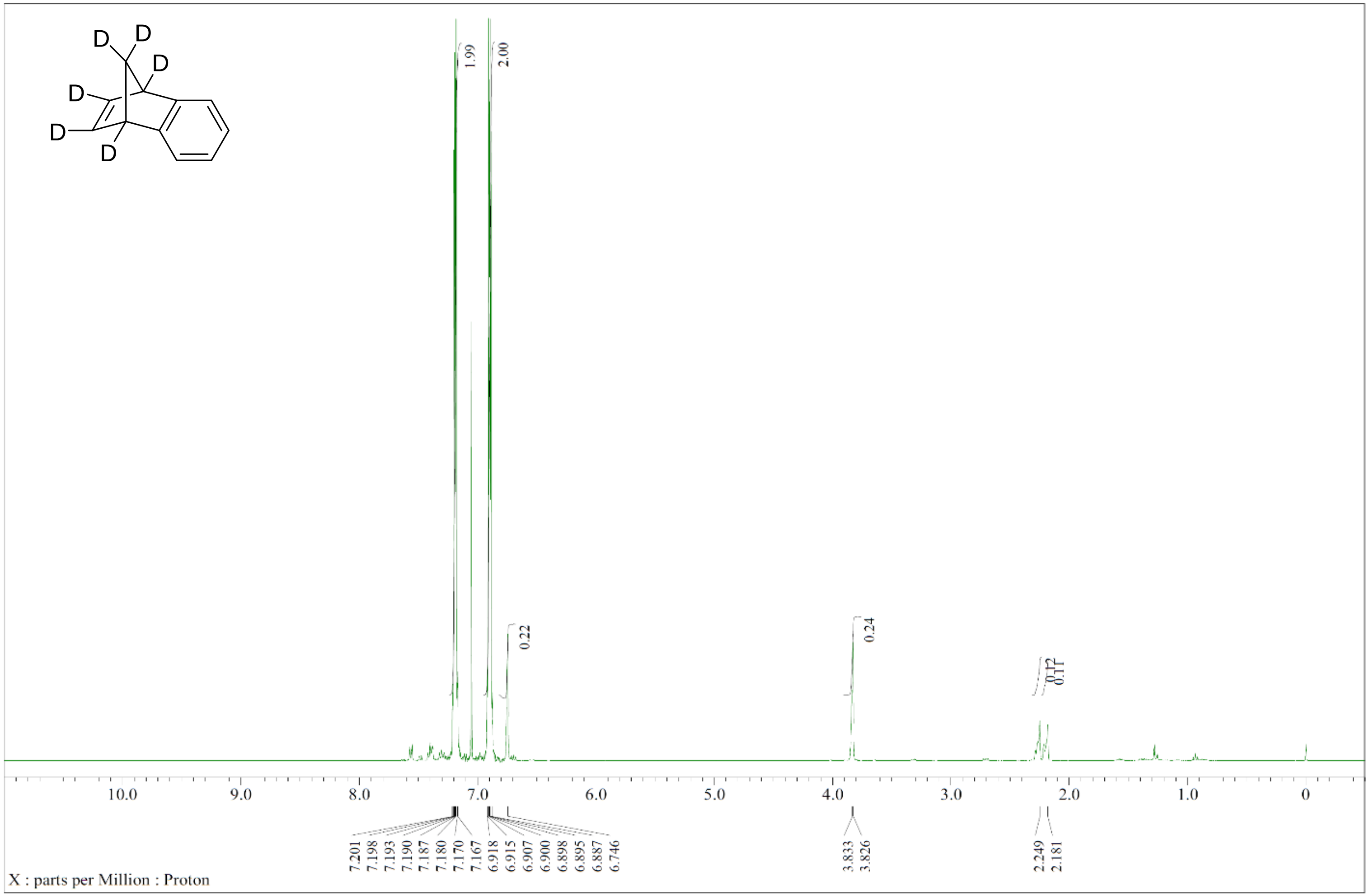

Supplementary References

[1] Chatt, J. & Venanzi, L. M. Olefin co-ordination compounds. Part VI. Diene complexes of rhodium(I) *J. Chem. Soc.* **0**, 4735-4741 (1957).

[2] Shibata, K. & Chatani, N. Rhodium-Catalyzed Alkylation of C–H Bonds in Aromatic Amides with α,β-Unsaturated Esters. *Org. Lett.* **16**, 5148-5151 (2014).

[3] Lambert, J. B. & Finzel, R. B. Competition between modes of solvolytic participation in 3-cyclopentenyl tosylate. *J. Am. Chem. Soc.* **105**, 1954-1958 (1983).

[4] Coe, J. W., Wirtz, M. C., Bashore, C. G. & Candler, J. Formation of 3-Halobenzyne: Solvent Effects and Cycloaddition Adducts. *Org. Lett.* **6**, 1589-1592 (2004).
